# Inferring decoding strategies for multiple correlated neural populations

**DOI:** 10.1371/journal.pcbi.1006371

**Published:** 2018-09-24

**Authors:** Kaushik J. Lakshminarasimhan, Alexandre Pouget, Gregory C. DeAngelis, Dora E. Angelaki, Xaq Pitkow

**Affiliations:** 1 Department of Neuroscience, Baylor College of Medicine, Houston, TX, United States of America; 2 Department of Basic Neuroscience, University of Geneva, Geneva, Switzerland; 3 Department of Brain and Cognitive Sciences, University of Rochester, Rochester, NY, United States of America; 4 Department of Electrical and Computer Engineering, Rice University, Houston, TX, United States of America; 5 Department of Mechanical and Aerospace Engineering, New York University, New York, United States of America; 6 Center for Neural Science, New York University, New York, United States of America; 7 Center for Neuroscience and Artificial Intelligence, Baylor College of Medicine, Houston, TX, United States of America; University of California at Berkeley, UNITED STATES

## Abstract

Studies of neuron-behaviour correlation and causal manipulation have long been used separately to understand the neural basis of perception. Yet these approaches sometimes lead to drastically conflicting conclusions about the functional role of brain areas. Theories that focus only on choice-related neuronal activity cannot reconcile those findings without additional experiments involving large-scale recordings to measure interneuronal correlations. By expanding current theories of neural coding and incorporating results from inactivation experiments, we demonstrate here that it is possible to infer decoding weights of different brain areas at a coarse scale without precise knowledge of the correlation structure. We apply this technique to neural data collected from two different cortical areas in macaque monkeys trained to perform a heading discrimination task. We identify two opposing decoding schemes, each consistent with data depending on the nature of correlated noise. Our theory makes specific testable predictions to distinguish these scenarios experimentally without requiring measurement of the underlying noise correlations.

## Author summary

The neocortex is structurally organized into distinct brain areas. The role of specific brain areas in sensory perception is typically studied using two kinds of laboratory experiments: those that measure correlations between neural activity and reported percepts, and those that inactivate a brain region and measure the resulting changes in percepts. The two types of experiments have generally been interpreted in isolation, in part because no theory has been able combine their outcomes. Here, we describe a mathematical framework that synthesizes both kinds of results, giving us a new way to assess how different brain areas contribute to perception. When we apply our framework to experiments on behaving monkeys, we discover two models that can explain the perplexing finding that one brain area can predict an animal’s reported percepts, even though the percepts are not affected when that brain area is inactivated. The two models ascribe dramatically different efficiencies to brain computation. We show that these two models could be distinguished by a proposed experiment that measures correlations while inactivating different brain areas.

## Introduction

Although much is known about how single neurons encode information about stimuli, how neurons contribute to reported percepts is less well understood[1]. The latter, called the “decoding problem”, seeks to identify how the brain uses the information contained in neuronal activity. Although some studies have sought to understand *principled* ways to decode population responses in the presence of correlated noise [2–12], the rules by which the brain *actually* integrates information across noisy neurons remain unclear.

Neuroscientists have traditionally investigated this question using two distinct approaches: causal or correlational. In causal approaches, experimenters selectively activate or inactivate brain regions of interest, and measure resulting perceptual or behavioural changes. In correlational approaches, experimenters measure correlations between behavioural choices and neuronal activity, typically quantified by ‘choice probability’ (reviewed in Ref. [13]) or, more straightforwardly, by ‘choice correlation’ (CC)[14,15]. If CCs reflect a functional link between neurons and behaviour, one would expect brain areas with greater CCs to contribute more strongly to behaviour. This naïve view is contradicted by recent results that reveal a striking dissociation between the magnitude of CCs and the effects of inactivation across brain systems in rodents[16,17] and primates[18,19]. In hindsight, this apparent disagreement is not all that surprising because the two techniques, on their own, yield results whose interpretation is fraught with major difficulties.

For instance, the CC of a neuron depends not only on its direct influence on behaviour but also on the influence of all the other neurons with which it is correlated. As an extreme example, a neuron that is not decoded at all could be correlated with one that is, and thus exhibit choice-related activity[9]. Recent theoretical results show that it is possible, in principle, to use knowledge of noise correlations to extract decoding weights from CCs[14]. However, directly measuring the correlational structures that matter for decoding may be extremely difficult[20]. This problem is compounded by the fact that behaviourally relevant information may be distributed across neurons in multiple brain areas, so neuronal CCs in one area may depend on activity in other areas. Moreover, in causal approaches, inactivation of one brain area could lead to a dynamic recalibration of decoding weights from other areas. Therefore, changes in behavioural thresholds following inactivation may not be commensurate with the contribution of the area.

When analysed in conjunction, however, results from correlational and causal studies may together provide constraints that can be used to precisely determine the relative contributions of the brain areas involved. In this work, we extend recent theories[14,15,20] and propose a general framework for inferring decoding weights of neurons across multiple brain areas using CCs and changes in behavioural threshold following inactivation. The two quantities together provide a direct estimate of the relative contributions of different areas without needing to precisely measure the correlation structure. This analysis is based on coarse-grained models of decoded neural noise that is correlated across populations. We demonstrate our technique by applying it to data from macaque monkeys trained to perform a heading discrimination task. In this task, there is a known discrepancy[18,21–23] between CCs and the effects of inactivating two brain areas: although neurons in the ventral intraparietal (VIP) area were found to be substantially better predictors of the animal’s choices than dorsal medial superior temporal (MSTd) neurons, performance is impaired by inactivating MSTd but not VIP. We use our framework to extract key properties of the decoder that can account for these counter-intuitive results. To our surprise, we find that, depending on the structure of correlated noise, experimental data are consistent with two opposing schemes that attribute either too much or too little weight to VIP. We use our theory to make specific testable predictions to distinguish these schemes using CCs measured during inactivation, again without measuring the detailed noise correlations.

## Results

Our framework for understanding neural decoding involves three main ingredients: an analysis of choice correlations and discrimination thresholds, two classes of models for noise correlations with different information content, and coarse-grained descriptions of those models for multiple populations. Our analysis proceeds as follows. We begin in section **Decoding framework** with some core definitions for neural population responses and estimation tasks based on decoding from multiple populations. Then, in the section **Analysis of choice correlations**, we describe the expected patterns of choice-related activity under the assumptions of optimal and suboptimal decoding. These patterns depend on the structure of neural noise, so in the section, **Models of neural variability**, we next describe two fundamentally different noise models, whose information content is extensive (*i*.*e*. growing with population size) or limited. We then refine these models for multiple populations in the section **Coarse-grained noise models for multiple populations**. Next we return to choice correlations to explore consequences of this coarse-grained description in the section **Coarse-grained choice correlations**. Our general theoretical analysis concludes in **Combining choice correlations and inactivation effects to infer decoding of distinct populations**. Finally, we specialize this theory to two populations as we apply it to experimental data.

Some readers wishing to skip some of the mathematical details may wish to read the sections **Decoding framework**, which sets out the basic concepts we invoke, and **Models of neural variability**, which describes the two main noise models we contrast, before jumping to **Application to neural data**.

### Decoding framework

We consider a linear feedforward network in which the firing rates **r** = [*r*_1_,…,*r*_*N*_] of the *N* neurons are tuned to the stimulus *s* as **f**(*s*) = 〈**r**|*s*〉, where the angle brackets denote an average over trials conditioned on the stimulus. The responses on a single trial differ from their averages by some noise with variance $\sigma_{k}^{2}$ for neuron *k*, and exhibit a covariance *Σ* = 〈**rr**^T^|*s*〉 − **f**(*s*)**f**(*s*)^T^ that we assume is stimulus-independent. These neural responses are combined linearly using weights **w** to yield a locally unbiased estimate $\hat{s}$ of the stimulus according to $\hat{s}=w^{T}(r-f(s_{0}))+s_{0}$. Here *local* means that the stimulus is near a reference *s*_0_, which we will now take to be 0 without loss of generality, and **f**(*s*_0_) is the mean population response to that reference. *Unbiased* estimation means that the estimate is accurate on average, so that $\langle \hat{s}|s\rangle =s$. In the experiments we model, the animals indeed are unbiased after training.

The performance of a decoder is often characterized by the variance *ε* of its estimate:

$$\begin{array}{c} \varepsilon =\langle {\hat{s}}^{2}\rangle -{\langle \hat{s}\rangle }^{2}=\langle {\left(w^{T}r\right)}^{2}\rangle -{\left(w^{T}f\right)}^{2}=w^{T}\Sigma w \end{array}$$ (1)

Other common measures of performance are the discrimination threshold *ϑ*, sensitivity index, *d*′, and Fisher information *J*. These measures are all closely related. We will often refer to the discrimination threshold *ϑ*, which is the stimulus difference, Δ*s*, required for reliable binary discrimination between two categories when discrimination is based on an estimator with finite variance. When 'reliable' is 68% correct, then this threshold is just the estimate's standard deviation, $\vartheta =\sqrt{\varepsilon }$. This definition coincides with the sensitivity index $d^{\prime }=\Delta \mu /\sigma_{\hat{s}}=1$, when the mean difference, Δ*μ*, between estimates for the two stimuli is the same size as the standard deviation, $\sigma_{\hat{s}}$, of those estimates. When the neural response mean **f**(*s*) is tuned to the stimulus, but other statistics do not provide additional information (*i*.*e*. for responses drawn from the exponential family), then the Fisher information, *J*, is exactly equal to the inverse variance of an unbiased, locally optimal linear estimator: *J* = 1/*ε* (also assuming differentiable tuning curves and non-singular noise covariance).

Many experiments assess performance using a two-alternative forced-choice experiment (2AFC). They quantify performance by the discrimination threshold, *ϑ*, which is the stimulus difference required for reliable binary discrimination (68% correct) (see **Methods**), and assess neural decoding based on choice probabilities[24]. However, theoretical results about decoding are much simpler when applied to continuous estimation (which we will consider to be a continuous ‘choice’). Conveniently, local continuous estimation and fine discrimination are closely related. For example, as mentioned above, the discrimination threshold *ϑ* is equal to the standard deviation of an unbiased local estimator, $\sigma_{\hat{s}}$, if the output variability is Gaussian. Under the same assumptions, choice correlation has a simple near-affine relation to choice probability (see **Methods**, [15]). We thus first describe the theory in terms of a local estimation task, and later apply the suitable transformations when we analyze data from binary discrimination tasks.

If the brain decodes signals linearly from multiple populations of neurons, its overall estimate $\hat{s}$ can always be expressed as a linear combination of unbiased estimates from each population separately:

$$\begin{array}{c} \hat{s}=a^{T}\hat{s} \end{array}$$ (2)

where $\hat{s}=[{\hat{s}}_{1},\ldots ,{\hat{s}}_{Z}]$ is a vector of separate estimates from each of *Z* populations, and ***a*** is a vector of *scaling factors* for each estimate to create one overall estimate. We call these ‘scaling factors’ to distinguish them from the weights given to individual neurons. Thus the problem of decoding multiple populations can be viewed as one of scaling and combining estimates from individual populations. Note that this is equivalent to a single linear decoder of all populations together using **w** = [*a*_1_**w**_1_ ⋯ *a*_*Z*_**w**_*Z*_].

For locally linear decoding, the assumption of no bias implies a normalization constraint on the weights and scaling factors. An unbiased estimate should match the stimulus, on average; and so a change in the estimate should match the change in the stimulus, on average: $\partial_{s}\langle \hat{s}|s\rangle =\partial_{s}w^{T}(f(s)-f(0))\approx w^{T}f^{\prime }(s)=\partial_{s}s=1$. Analogously, unbiased scaling factors of individually unbiased estimates ${\hat{s}}_{z}$ satisfy $a^{T}\partial_{s}\langle \hat{s}|s\rangle =a^{T}1=1$, where **1** is a vector of all ones and where each population estimate ${\hat{s}}_{x}=w_{x}^{T}(f_{x}(s)-f_{x}(0))$ obeys the normalization $w_{x}^{T}f_{x}^{\prime }(s)=1$.

**Fig 1:**
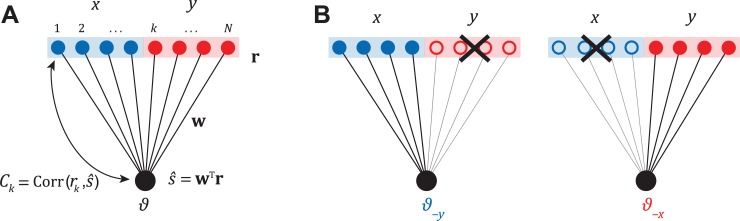
Experimental strategies. **(A)** An illustration of a feedforward network with linear readout. The decoder linearly combines the activity **r** of neurons in two populations *x* and *y* with weights **w**, to produce an estimate $\hat{s}$ of the stimulus. Activity of individual neurons *r*_*k*_ is correlated with $\hat{s}$ and is quantified by either the choice probability *CP*_*k*_, or the closely related choice correlation *C*_*k*_. In an optimal system, the weights **w** generate choice correlations that satisfy **Eq (4)**. **(B)** In inactivation experiments, the neurons from each population are inactivated and the resulting changes in behavioural threshold are recorded.

Using this decomposition into populations, we can dissociate how the weight *patterns* within each subpopulation (**w**_*x*_) and their *scaling factors* (*a*_*x*_) affect the output of the decoder. This mathematical separation is also appealing because it provides a common framework to synthesize results from experiments conducted at two fundamentally different levels of granularity. One class of experiments involves making fine measurements such as the correlation between trial-by-trial fluctuations in the activity *r*_*k*_ of an individual neuron *k* and the animal’s decision (**Fig 1A**). The second class of experiments studies causation by measuring behavioural effects of inactivating certain candidate brain areas. For perceptual discrimination tasks, this is done by comparing coarse measures such as the animal’s behavioural performance before (*ϑ*) and after (*ϑ*_−*x*_) inactivating population *x* (**Fig 1B**).

We would like to use these experimental measurements to identify the relative behavioural contributions of various brain areas. Therefore we will present a technique to infer neuronal readout weights in multiple brain areas, focusing primarily on how to extract the scaling factors, *a*_*x*_, of the brain areas rather than the fine structures, **w**_*x*_, of their decoding weights.

### Analysis of choice correlations

Choice correlation of a neuron *k* is defined as the correlation coefficient between its response *r*_*k*_ and the animal’s estimate of the stimulus $\hat{s},\;C_{k}=Corr(r_{k},\hat{s}|s)$, across repeated trials with the same stimulus *s*. Substituting the estimate into this correlation, we find:

$$\begin{array}{c} C_{k}=\frac{Cov(r_{k},r^{T}w|s)}{\sqrt{Var(r_{k}|s)Var(r^{T}w|s)}}=\frac{\langle r_{k}r^{T}w\rangle -\langle r_{k}\rangle \langle r^{T}w\rangle }{\sqrt{\sigma_{k}^{2}(\langle w^{T}rr^{T}w\rangle -\langle w^{T}r\rangle \langle r^{T}w\rangle )}}=\frac{{\left(\Sigma w\right)}_{k}}{\sqrt{\sigma_{k}^{2}w^{T}\Sigma w}} \end{array}$$ (3)

where the noise variance for neuron *k* is $Var(r_{k}s)=\sigma_{k}^{2}=\Sigma_{kk}$. All neurons' choice correlations can then be expressed together in vector form as $C=\frac{S^{-1}\Sigma w}{\sqrt{w^{T}\Sigma w}}$, where *S* is a diagonal matrix of the standard deviations.

These choice correlations follow a particularly simple pattern if readout weights are locally optimal [15] as obtained from linear regression as **w** ∝ *Σ*^−1^**f**′. If we substitute these optimal weights into **Eq (3)**, the inverse covariance from the weights cancels the covariance driving the choice correlations:

$$\begin{array}{l} C_{k,\mathrm{opt}}=\frac{{\left(\Sigma \Sigma^{-1}f^{\prime }\right)}_{k}}{\sqrt{{\left(\Sigma^{-1}f^{\prime }\right)}^{T}\Sigma (\Sigma^{-1}f^{\prime })\sigma_{k}^{2}}}\\ \;=\frac{f_{k}^{\prime }}{\sigma_{k}}\frac{1}{\sqrt{{f^{\prime }}^{T}\Sigma^{-1}f^{\prime }}}\\ \;=\frac{\vartheta }{\vartheta_{k}} \end{array}$$ (4)

where *C*_*k*,opt_ is the choice correlation of neuron *k* expected from optimal decoding, $\vartheta_{k}=f_{k}^{\prime }/\sigma_{k}$ is the discrimination threshold of neuron *k* (or, equivalently, the standard deviation of an unbiased estimator based only on that neuron’s response), and *ϑ* is the behavioural discrimination threshold. If decoding were optimal, then this behavioural threshold will match the standard deviation of a locally optimal unbiased estimator based on the whole population, *ϑ* = (**f**′^*T*^*Σ*^−1^**f**′)^−1/2^. By itself, such a match would be strong evidence for optimal decoding, but testing this would require recording from all relevant neurons in the brain. The relationship in **Eq (4)** is thus a far more practical test for optimal decoding.

If all neurons from multiple populations satisfy the above equation, this gives us strong evidence that the neuronal weights — and consequently also the relative scaling factors ***a*** of different populations — are optimal. As we will see later, the exact values of ***a*** can then be directly extracted from the behavioural thresholds following inactivation of those areas.

The pattern of choice correlations generated by any generic *sub*optimal decoder is more complicated, as it depends explicitly on the structure of noise covariance and the readout weights [14]. For a population of *N* neurons, the noise covariance *Σ* describes, for a fixed stimulus, the power along *N* orthogonal modes of variation. Each of these modes could contribute to the overall choice correlation, depending on how strongly that mode is decoded. We express the decoding weights of a suboptimal decoder in terms of the covariance, as **w** = (*Σ*^−1^**g**)/**f**′^T^*Σ*^−1^**g** where **g** could be any vector in $R^{N}$. The normalization ensures that this decoder is locally unbiased, satisfying **w**^*T*^**f**′ = 1.

$$\begin{array}{c} C=\frac{S^{-1}\Sigma w}{\sqrt{w^{T}\Sigma w}}=\frac{S^{-1}\Sigma w}{\vartheta }=\frac{S^{-1}}{\vartheta {f^{\prime }}^{T}\Sigma^{-1}g}g \end{array}$$ (5)

Note that this recovers the optimal expression given by equation (4) if **g** is replaced by **f**′. We now rewrite **g** in the basis of the eigenmodes **u**^*i*^ of the covariance *Σ*, using $g=\sum_{i=1}^{N}u^{i}{u^{i}}^{T}g$. By multiplying and dividing by ${u^{i}}^{T}f\prime$, we can decompose the choice correlations for a suboptimal decoder into a weighted combination of optimal choice correlations patterns $C_{opt}^{i}$ arising from each eigenmode:

$$\begin{array}{l} C=\frac{S^{-1}}{\vartheta {f^{\prime }}^{T}\Sigma^{-1}g}\sum_{i=1}^{N}u^{i}{u^{i}}^{T}g\\ \;=\frac{S^{-1}}{\vartheta {f^{\prime }}^{T}\Sigma^{-1}g}\sum_{i=1}^{N}u^{i}\left({u^{i}}^{T}f^{\prime }\right)\frac{\left({u^{i}}^{T}g\right)}{\left({u^{i}}^{T}f^{\prime }\right)}\\ \;=\sum_{i=1}^{N}\beta_{i}C_{\mathrm{opt}}^{i} \end{array}$$ (6)

where

$$\begin{array}{c} C_{opt}^{i}=\vartheta \;S^{-1}u^{i}({u^{i}}^{T}f^{\prime }) \end{array}$$ (7)

$C_{opt}^{i}$ is essentially the *i*'th noise mode **u**^*i*^ rescaled by the individual neural sensitivity, and $\beta_{i}=\frac{1}{\vartheta^{2}({f^{\prime }}^{T}\Sigma^{-1}g)}\frac{\left(g^{T}u^{i}\right)}{\left({f^{\prime }}^{T}u^{i}\right)}$. These multipliers *β*_*i*_ reflect the extent of suboptimality. When decoding weights are optimal, then the readout direction (again in units of the covariance) is **g** = **f**′, leading to *β*_*i*_ = 1 for all *i*. Thus, for optimal decoding the above equation reduces to **Eq (4)**.

In principle, elements of *β*_*i*_, and thus properties of the decoding weights, can be estimated by regressing measured choice correlations against individual columns of the matrix of choice correlations *C*_opt_ predicted by optimal decoding. In practice, it is very difficult to estimate all of the multipliers *β*_*i*_ because the components $C_{k,opt}^{i}$ depend on the individual noise modes of *Σ* (**Eq (7)**). Directly measuring *Σ* is a notoriously challenging task [20] that involves simultaneously recording the activity of a large population of neurons, and is nearly impossible for certain areas due to the geometry of the brain. Even if such recordings could be performed, it would be challenging to get an accurate assessment of the fine structure of the covariance with limited data, since the number of parameters to measure increases with population size faster than the number of measurements. Fortunately, since neuronal choice correlations are measurably large, it follows that one can infer the animal’s decoding weights with reasonable precision by estimating the few leading multipliers that depend only on the most dominant modes of covariance. This is because if the correlated noise modes with small variance were to dominate the decoder, then only a tiny fraction of each neuron’s variations would propagate to the decision, leading to immeasurably small choice correlations[15] (**S1 Fig**). It is possible to model properties of the leading modes of covariance without large-scale recordings, and we will consider two different noise models: *extensive information* and *limited information*.

### Models of neural variability

#### Extensive information model

A common way to measure important components of the covariance structure is through pairwise recordings. Noise covariance measured between pairs of neurons can be modeled as a function of their response properties, such as the difference in their preferred stimulus or the similarity of their tuning functions, to obtain empirical models of noise.

One such model is limited-range noise correlations[25–30], so called because they are proportional to signal correlation and thereby limited in range to pairs with similar tuning. We use this model to approximate a full noise covariance for all neurons in the population[31,32]. Specifically, we assume that the typical noise correlation coefficient ${\bar{R}}_{ij}$ between responses of two neurons *i* and *j* is given by

$$\begin{array}{c} {\bar{R}}_{ij}=(1-m)\delta_{ij}+mR_{ij}^{sig} \end{array}$$ (8)

where $R_{ij}^{sig}=Corr(f_{i},f_{j})$ is the signal correlation, i.e. the correlation coefficient between neurons' mean responses over a uniform distribution of stimuli *s* and the proportionality *m* between signal and noise correlations can be empirically determined (see **Methods**). To match Poisson-like properties of neural responses, model variances are set equal to the mean responses, and this scaling produces a covariance of $\Sigma_{ij}=R_{ij}\sqrt{f_{i}f_{j}}$. This has been a common noise model in the study of population codes[25–30]. Although the resulting covariance matrix is unlikely to capture fine details accurately, if the model is reasonable then most of the variance would be captured by the leading modes.

In an extensive information model, the amount of information encoded by the neural activity grows with population size [33–35], hence the name. If the brain extracts information by a decoder restricted only to the noisiest subspace given by these leading noise modes, this would recover just a tiny fraction of the total available information. Although this is radically suboptimal, this is the only way an extensive information model can explain the large magnitude of neuronal choice correlations[15].

#### Limited information model

Extensive information models are based on measurements of neural populations but, as we mentioned above, current recordings are not sufficient to measure or even infer the covariance matrix *in vivo*. It is therefore possible that information in cortex is not extensive. Indeed, the extensive information model conflicts with the fact that cortical neurons receive their inputs from a smaller population of neurons. The cortex must then inherit not only the input signal but also any noise in that input. This generates information-limiting correlations [15,20] in the cortex, a form of correlated noise that looks exactly like the signal and thus cannot be averaged away by adding more cortical neurons. Since inferring the brain’s decoding weights from choice-related activity depends on the noise covariance, we also consider the consequences of information-limiting correlations.

For fine discrimination between two neighboring stimuli *s* and *s* + *δs*, the signal is given by the change in mean population responses **f**(s + δs) − **f**(s) ≈ δs **f**′(*s*). Information-limiting correlations for this task thus fluctuate along the direction **f**′, generating a covariance containing differential correlations [20] — that is, a covariance component proportional to **f**′**f**′^T^. The constant of proportionality, which we denote as *ε*, represents the variance of information-limiting correlations. According to this model, the total noise covariance *Σ*_IL_ for the information-limiting model can be decomposed into a general noise covariance *Σ* (which we assume follows the extensive information model) and the information-limiting component:

$$\begin{array}{c} \Sigma_{IL}=\Sigma +\varepsilon f^{\prime }f^{\prime {}^{T}} \end{array}$$ (9)

The variance of a locally optimal linear estimator based on a neural population with this noise covariance is given by [20]:

$$\begin{array}{c} \langle {\delta \hat{s}}^{2}\rangle ={{[f}^{\prime T}\Sigma_{IL}^{-1}f^{\prime }]}^{-1}={\left(f^{\prime T}\Sigma^{-1}f^{\prime }\right)}^{-1}+\varepsilon \approx \varepsilon \end{array}$$ (10)

where we have used the Sherman-Morrison lemma to invert *Σ*_IL_. The estimator variance due to the extensive information term (**f**′^T^*Σ*^−1^**f**′)^−1^ shrinks with population size [20,33,34], and is eventually dominated by the information-limiting noise variance *ε*. With increasing population size, both the signal **f**′ and the information-limiting component *ε***f**′**f**′^T^ grow identically, eventually resulting in no further improvement in signal-to-noise ratio, and thus no improvement in discriminability. In general, *ε* could be very small, and hence information-limiting correlations may be very hard to detect with limited data as they are easily swamped by noise arising from other sources. Nevertheless, this noise has enormous implications for decoding large populations because it limits the total information to 1/*ε*.

### Coarse-grained noise models for multiple populations

In this section we describe these two noise correlation models coarsely, at the population level, so that we can use the shared fluctuations between populations to reveal the decoder's scaling factors. To attribute scaling factors to each of *Z* decoded populations, one must consider at least *Z* modes of the noise covariance, one per population. We will restrict our attention to decoders inhabiting only these leading modes. If there are *Z* dominant noise modes and they are correlated across populations, then we can approximate *Σ* with a rank-*Z* noise covariance matrix composed of both independent and correlated noise between the populations.

#### Multi-population limited information model

When dealing with multiple populations (e.g., in different brain areas), one has to keep in mind that although they may together receive limited information, they need not inherit it from exactly the same upstream neurons. Therefore, we construct a more general model allowing the different populations to receive both distinct and shared information. To describe this, we separate a low-rank information-limiting fluctuations from a general noise covariance ***Σ*** (which we assume follows the extensive information model),

$$\begin{array}{c} \Sigma_{IL}=\Sigma +FEF^{T} \end{array}$$ (11)

Here *F* is an *N*×*Z* block-diagonal matrix

$$\begin{array}{c} F=(\begin{array}{ccc} f_{1}^{\prime } & 0 & 0\\ 0 & \ddots & 0\\ 0 & 0 & f_{Z}^{\prime } \end{array}) \end{array}$$ (12)

and $f_{z}^{\prime }$ is a vector of stimulus sensitivities for all neurons in population *z*, with elements $f_{iz}^{\prime }$, and *E* is a *Z*×*Z* covariance for information-limiting noise in each population. The covariance between two neurons in this more general information-limiting model would still be proportional to the product of the derivative of their tuning curves. However, the constant of proportionality varies depending on whether the neurons are both from the same population *x* (*E*_*xx*_), both from *y* (*E*_*yy*_), or from different populations (*ε*_*xy*_):

$$\begin{array}{c} \Sigma_{IL}=\Sigma +(\begin{array}{ccc} \varepsilon_{xx}f_{x}^{\prime }{f_{x}^{\prime }}^{T} & \varepsilon_{xy}f_{x}^{\prime }{f_{y}^{\prime }}^{T} & \ldots \\ \varepsilon_{xy}f_{x}^{\prime }{f_{y}^{\prime }}^{T} & \varepsilon_{yy}f_{y}^{\prime }{f_{y}^{\prime }}^{T} & \ldots \\ \ldots & \ldots & \ddots \end{array}) \end{array}$$ (13)

Analogous to the information-limiting noise variance *ε* in the single population case (**Eq (10)**), elements of *E* once again determine the variance of the locally optimal linear estimators (and thus optimal discrimination thresholds) for individual populations, as well as for all populations together (**S2 Text**). We call the noise $\varepsilon_{xx}f_{x}^{\prime }{f_{x}^{\prime }}^{T}$ in each population *x* “*locally* information-limiting noise” because it is local to one population *x*. For large populations with this noise structure, the total information content within population *x* alone is limited to 1/*ε*_*xx*_.

By itself, this local noise does not guarantee that the complete population is globally information-limited: that depends on how the noise in different populations is correlated. For example, input from another brain area might add some locally information-limiting noise[36], which could in principle be removed again by appropriately decoding both brain areas together. Depending on the covariance between information-limiting noise across populations, *ε*_*xy*_, different populations may contain completely redundant, independent, or synergistic information [37,38]. However, the information in all populations together may be limited as well, ultimately by the ***f***′***f***′^*T*^ component of the covariance *Σ*. We call this component “globally information-limiting noise”.

Correlations that limit information also cause redundancy. As a consequence, many different decoding weights extract essentially the same information. The population is then robust to some amount of suboptimal decoding, which makes it easier to achieve near-optimal behavioural performance [15]. In the locally information-limited noise model for multiple populations described above, this robustness also holds within each population individually. In this case, a separate decoder for each population *x* produces an estimate ${\hat{s}}_{x}$ that is near-optimal for the corresponding areas. Importantly, however, these estimates may have different variances, and may even covary, so they need to be properly combined to produce a good single estimate according to **Eq (2)**. While information-limiting correlations within each area would make the system generally robust to the choice of weight patterns **w**_*x*_, suboptimality could yet arise from an incorrect scaling *a*_*x*_ of each individually near-optimal estimate. This is because after the dimensionality reduction from large redundant populations down to a single unbiased estimate per population, most of the redundancy has been squeezed out: just one degree of freedom remains for the decoder, so different ways of combining the estimates are not equivalent.

#### Multi-population extensive information model

For the extensive information model, we can also define a useful rank-*Z* approximation of the relevant components of the noise covariance ***Σ***. Let **u**_*x*_ denote the leading eigenvector of population *x*'s covariance ***Σ***_*xx*_, with corresponding eigenvalue *λ*_*x*_. Note that these are not the eigenvectors of the full covariance matrix, just of the covariances for each population separately. If, in the full covariance, the leading modes of different populations ***x*** and ***y*** interact to produce correlated noise with strength *λ*_*xy*_, then we approximate the full covariance by ***Σ*** = *ULU*^T^ where, analogously with Eq (12),

$$\begin{array}{c} U=(\begin{array}{ccc} u_{1} & 0 & 0\\ 0 & \ddots & 0\\ 0 & 0 & u_{Z} \end{array}) \end{array}$$ (14)

and the *Z*×*Z* matrix

$$\begin{array}{c} L=(\begin{array}{ccc} \lambda_{1} & \cdots & \lambda_{1Z}\\ \vdots & \ddots & \vdots \\ \lambda_{1Z} & \cdots & \lambda_{Z} \end{array}) \end{array}$$ (15)

In the extensive information model, an optimal decoder would largely avoid the largest noise modes. However, optimal decoding of the extensive model is thoroughly ruled out by experimental measurements described below (see section ‘Test for Optimality’). Thus, for our coarse-grained multi-population model, we assume the brain's decoder is limited to the noisiest mode for each population, while it has complete freedom to combine estimates derived thusly from each population. Future refinements of this coarse-grained framework could consider decoding other modes per population instead, or more modes.

Unlike elements of information-limiting noise *E* in **Eq (13)**, elements of *L* cannot be directly related to the variance of the output estimator $\hat{s}$ because the latter depends not only on the magnitude of noise (*λ*_*x*_) but also on the signal ($u_{x}^{T}f_{x}^{\prime }$). But we can rescale each element of *L* to obtain *E*, and express a low-rank approximation of the covariance *Σ* in terms of *E* as:

$$\begin{array}{c} \Sigma =U(U^{T}F)E(U^{T}F{)}^{T}U^{T} \end{array}$$ (16)

where *E* = (*U*^T^*F*)^−1^*L*(*U*^T^*F*)^−1^, so the elements of *E* are related to *L* as: $\varepsilon_{xx}=\frac{\lambda_{x}}{{\left(u_{x}^{T}f_{x}^{\prime }\right)}^{2}}$ and $\varepsilon_{xy}=\frac{\lambda_{xy}}{\left(u_{x}^{T}f_{x}^{\prime })(u_{y}^{T}f_{y}^{\prime }\right)}$. Just like the case of information-limiting noise, the elements of *E* again determine optimal thresholds according to **S2 Text** (Eqn (S2.1) – (S2.2)), but with one key distinction: whereas those thresholds correspond to the output of optimal decoding for each population in the case of information-limiting noise, these correspond to outputs of optimal decoding only within the subspace of the *Z* populations' leading modes in the case of extensive information model. Note that we can use the formulation in **Eq (16)** to derive information-limiting noise (**Eq (11)**) as a special case by using $u_{x}=f_{x}^{\prime }/\|f_{x}^{\prime }\|$ to recover *Σ* = *FEF*^T^.

### Coarse-grained choice correlations

These coarse-grained representations of population variability reflect the dominant decoded mode in each population. This level of description allows us to focus on how information is combined between populations. If the brain indeed combines activity from different areas suboptimally, then simplifying **Eq (6)** in the presence of information-limiting correlations gives choice correlations within each area that are not equal to the optimal choice correlations, but are still proportional to them.

$$\begin{array}{l} C=\frac{S^{-1}\Sigma w}{\sqrt{w^{T}\Sigma w}}\approx \frac{S^{-1}FEF^{T}w}{\sqrt{w^{T}\Sigma w}}\approx \frac{S^{-1}FEa}{\sqrt{a^{T}Ea}}\\ \;=\frac{Ea}{a^{T}Ea}\sqrt{a^{T}Ea}(S^{-1}F)=\frac{Ea}{a^{T}Ea}\frac{\vartheta }{\vartheta_{k}}\\ \;=\beta \frac{\vartheta }{\vartheta_{k}} \end{array}$$ (17)

where $\beta_{x}=\frac{{\left(Ea\right)}_{x}}{a^{T}Ea}$. Under conditions of suboptimality, choice correlations in different brain areas *x* may have different multipliers *β*_*x*_ which depend on the scaling of the brain areas and on the covariance between the estimates ${\hat{s}}_{x}$ that can be derived from them. These multipliers *β*_*x*_ can be directly identified by regressing measured choice correlations against *ϑ*/*ϑ*_*k*_, the choice correlations predicted for optimal decoding. **S4 Text** shows that a similar relation holds for the extensive information model when only the leading mode of each population is decoded (**S4 Text** – Eqn (S4.1)).

### Combining choice correlations and inactivation effects to infer decoding of distinct populations

In the previous section, we showed how to reduce the fine structure of choice correlations down to one number for each population, the slope *β*_*x*_ of its choice correlation. We will now show how these multipliers can be used, together with the behavioural thresholds *ϑ* following inactivations of different brain areas, to infer the relative scaling of their weights ***a***. First we describe the main approach in the general setting with multiple populations, and then we specialize to the particular case of two populations and apply it to our data.

Previous work has shown how one can combine knowledge of choice correlations and neural noise correlations to estimate the decoding weights of individual neurons[14]. If decoded neural responses in each population are dominated by a single mode, then we can extend this concept to the population level. The population-level analog of a neural response *r*_*k*_ is an estimate ${\hat{s}}_{x}$ derived from population *x*. The analog of choice correlations *C*_*k*_ are the slopes *β*_*x*_ that relate observed and optimal choice correlations, and the analog of noise covariance *Σ*_*ij*_ between neurons *i* and *j* is the covariance *ε*_*xy*_ (**Eqs (11)** & **(14)**) between estimates ${\hat{s}}_{x}$ and ${\hat{s}}_{y}$ derived from distinct populations.

Unlike neural noise correlations, we cannot directly measure the noise correlations *E* at the population level. Nonetheless, we can infer those population-level noise correlations indirectly from inactivation experiments, in which behavioral thresholds are measured after altering the decoder scaling afforded to different brain areas by a factor *ρ*_*xϕ*_ for inactivation experiment number *ϕ*. In our feedforward linear model, it is mathematically equivalent to reduce the activity by *ρ*_*xϕ*_, or to alter a decoder's scaling *a*_*x*_ by the same factor. Totally inactivating an area is equivalent to setting its scaling to zero, but here we permit partial inactivation of multiple brain areas. For now, we assume these inactivation factors are controlled by the experimenter, and thus known, although later we will incorporate some uncertainty about these inactivations.

Each such experiment provides one constraint on the unknown population properties, according to

$$\begin{array}{c} \theta_{\phi }^{2}\approx \frac{a_{\phi }\cdot E\cdot a_{\phi }}{{\left|a_{\phi }\right|}_{l_{1}}^{2}}=\frac{1}{{\left(\sum_{x}a_{x}\rho_{x\phi }\right)}^{2}}\sum_{xy}a_{x}\rho_{x\phi }E_{xy}\rho_{y\phi }a_{y} \end{array}$$ (18)

where *θ*_*ϕ*_ is the behavioural threshold during the *ϕ*’th inactivation experiment, ***a***_*ϕ*_ is the vector of decoder scaling factors for the different populations with components *a*_*xϕ*_ = *a*_*x*_*ρ*_*xϕ*_, and where the *l*_1_-normalization ${\left|a_{\phi }\right|}_{l_{1}}=\sum_{x}a_{x}\rho_{x\phi }$ ensures that the decoder remains unbiased after inactivation (as observed experimentally[18,22]). In such experiments one could also measure the slopes *β*_*xϕ*_ of the choice correlations for multiple different populations to provide additional measurement constraints

$$\begin{array}{c} \beta_{x\phi }\approx \frac{\delta_{x}\cdot E\cdot a_{\phi }}{{\left|a_{\phi }\right|}_{l_{1}}}=\frac{1}{\sum_{x}a_{x}\rho_{x\phi }}\sum_{y}E_{xy}\rho_{y\phi }a_{y} \end{array}$$ (19)

Notice that **Eqs (18)** and **(19)** can be written as multivariate polynomials up to cubic order jointly in the unknowns *E* and ***a***. Altogether there are *Z*(*Z*+1)/2 unknowns for the covariance matrix *E*, and another *Z* unknowns for the intact brain's decoder scaling factors ***a***. As long as the number of independent threshold and slope measurements is at least as large as the number of unknowns, then **Eq (19)** can be solved numerically (**S2 Fig**), revealing the correct decoder scaling for multiple populations. Slopes of choice correlations during inactivation experiments provides a larger number of data points from a given set of inactivation experiments than measuring the thresholds alone.

#### Two population solution

When only two populations of neurons, *x* and *y*, are relevant for a particular task, this general approach to identifying their relative scaling can be simplified. We next describe this simpler two-population theory, and then apply it to data from the vestibular system.

If we can completely inactivate one brain area, then from **Eq (1)**, the animal’s total estimate $\hat{s}$ would be equal to either ${\hat{s}}_{x}$ or ${\hat{s}}_{y}$, depending on which area is inactivated. The resultant behavioural threshold would simply reflect the variance of the remaining estimate, which is equal to the magnitude of dominant decoded noise within the active area, so $\vartheta_{-x}^{2}\approx \varepsilon_{yy}$ and $\vartheta_{-y}^{2}\approx \varepsilon_{xx}$. If populations *x* and *y* are uncorrelated (*ε*_*xy*_ = 0), then the ratio of weight scaling factors can be factorized into a product of ratios (**S5 Text**):

$$\begin{array}{c} \frac{a_{x}}{a_{y}}=\frac{\beta_{x}}{\beta_{y}}\frac{\varepsilon_{yy}}{\varepsilon_{xx}}\approx \frac{\beta_{x}}{\beta_{y}}\frac{\vartheta_{-x}^{2}}{\vartheta_{-y}^{2}} \end{array}$$ (20)

where the two independent factors represent outcomes of correlational and causal studies. If readout is optimal, then the multipliers *β*_*x*_ and *β*_*y*_ are both equal to one, so $a_{x}/a_{y}=\vartheta_{-x}^{2}/\vartheta_{-y}^{2}$. This is consistent with the general belief that the behavioural effects of inactivating a brain area must be commensurate with its contribution to the behaviour. A departure from optimality could break this relationship, so the effects of causal manipulation may not match the relative sensitivities of the brain areas (**S3 Fig**). Even in purely feedforward networks, the magnitude of neuronal choice correlations need not equal the effects of inactivation. Thus, disagreements between the two experimental outcomes should not be entirely surprising and do not undermine the functional significance of either.

In fact, **Eq (20)** revealed how one can combine choice correlations and behavioural thresholds to infer the contributions of two uncorrelated areas. But if the areas are correlated, one must explicitly account for the magnitude of correlation between areas *ε*_*xy*_ and the ratio of scales no longer factorizes:

$$\begin{array}{c} \frac{a_{x}}{a_{y}}\approx (\frac{\beta_{x}}{\beta_{y}}\frac{\vartheta_{-x}^{2}}{\vartheta_{-y}^{2}}-\gamma ){\left(1-\frac{\beta_{x}}{\beta_{y}}\gamma \right)}^{-1} \end{array}$$ (21)

where *γ* = *ε*_*xy*_ /*ε*_*xx*_ is the magnitude of correlated noise between the two populations’ estimates relative to the variance of estimates from *x* alone. Note that one can also use **Eqs (20)** and **(21)** to compute the optimal weight scaling factors simply by setting both *β*_*x*_ and *β*_*y*_ to 1. Therefore, we can use these equations not only to determine the relative weights of brain areas but to also to evaluate precisely how suboptimal those weights are.

### Application to data

**Fig 2:**
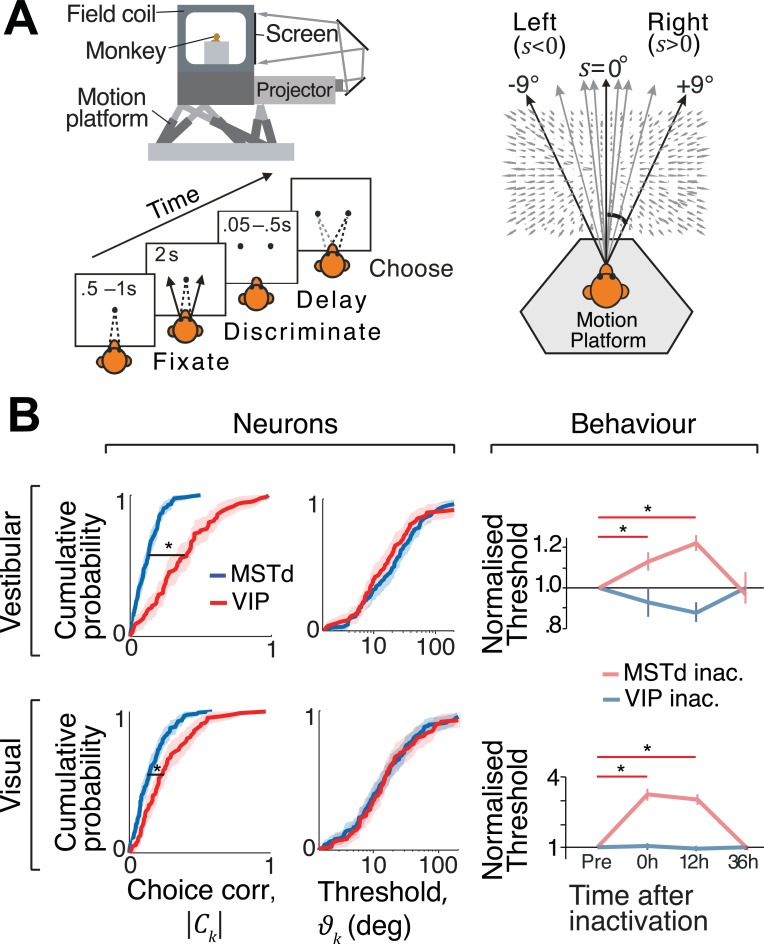
Choice-related activity and effects of inactivation. **(A)** Behavioural task: the monkey sits on a motion platform facing a screen. He fixates on a small target at the center of the screen, and then we induce a self-motion percept by moving the platform (vestibular condition) or by displaying an optic flow pattern on the screen (visual condition). The fixation target then disappears and the monkey reports his percept by making a saccade to one of two choice targets. **(B) Left**: Neurons in both MSTd (*n*=129) and VIP (*n*=88) exhibited significant choice correlations (CCs). The median CC of VIP neurons was significantly greater than that of MSTd neurons (*p<0.001, Wilcoxon rank-sum test) in both vestibular (top) and visual (bottom) conditions. **Middle**: Median neuronal thresholds were not significantly different between areas (vestibular: *p*=0.94, visual: *p*=0.86, Wilcoxon rank–sum test). **Right**: Average discrimination thresholds at different times relative to inactivation of VIP (unsaturated blue) and MSTd (unsaturated red). All threshold values were normalized by the corresponding baseline thresholds (“pre”). Shaded regions and error bars denote standard errors of the mean (SEM); asterisks indicate significant differences (*p<.05, *t*–test). Neural data re-analyzed from refs. [21,23]. Inactivation data reproduced from refs. [18,22].

We now use the techniques developed so far to infer the relative contributions of two brain areas in macaque monkeys to heading discrimination. Data were collected from monkeys trained to discriminate their direction of self-motion in the horizontal plane (**Fig 2A**) using vestibular (inertial motion) and/or visual (optic flow) cues (see **Methods**; see also refs. [21,23]). At the end of each trial, the animal reported whether their perceived heading $\hat{s}$ was leftward ($\hat{s}<0{}^{\circ}$) or rightward ($\hat{s}>0{}^{\circ}$) relative to straight ahead.

#### Discrepancy between correlation and causal studies

Responses of single neurons were recorded from either area MSTd (monkeys A and C; *n*=129) or area VIP (monkeys C and U; *n*=88) during the heading discrimination task (see Methods). Basic aspects of these responses were analyzed and reported in earlier work[21,23]. Briefly, it was found that neurons in VIP had substantially greater choice correlations (CC) than those in MSTd (Fig 2B – left) for both the vestibular and visual conditions. This difference in CC between areas could not be attributed to differences in neuronal thresholds *ϑ*_*k*_ (Fig 2B – middle), defined as the stimulus magnitude that can be discriminated correctly 68% of the time (*d*′=1) from neuron *k*’s response *r*_*k*_ (Methods; S3 Fig). Based on its greater CCs, one might expect that VIP plays a more important role in heading discrimination than MSTd. In striking contrast to this expectation, a recent study showed that there was no significant change in heading thresholds following VIP inactivation for either the visual or vestibular stimulus conditions[18] (Fig 2B – right (blue); monkeys B and J). On the other hand, inactivation of MSTd using a nearly identical experimental protocol led to substantial deficits in heading discrimination performance[22] (Fig 2B – right (red); monkeys C, J, and S). The neural and inactivation studies in VIP used non-overlapping subject pools, so the observed dissociation between CCs and inactivation effects could potentially reflect the idiosyncrasies of the subjects’ brains. To rule this out, we repeated the inactivation experiment by specifically targeting Muscimol injections to sites in area VIP that were previously found to contain neurons with high CCs in another monkey and obtained similar results (S5 Fig).

These findings reveal a striking dissociation between choice correlations and effects of causal manipulation: VIP has much greater CCs than MSTd yet inactivating VIP does not impair performance. One may be tempted to simply conclude that VIP does not contribute to heading perception. We will now show that this is not necessarily true. Depending on the structure of correlated noise and the decoding strategy, neurons in both areas may be read out in a manner that is entirely consistent with the observed effects of inactivation.

#### Test for optimality

We first asked if the above results can simply be explained if the brain allocated weights optimally to the two areas. To answer this, we tested if neuronal choice correlations satisfied Eq (4). Binary discrimination experiments typically do not measure choice correlations $C_{k}=Corr(r_{k},\hat{s}|s=s_{0})$ because they do not have direct access to the animal’s continuous stimulus estimate $\hat{s}$; they only track the animal’s binary choice. Instead they measure a related quantity known as choice probability defined as the probability that a rightward choice is associated with an increase in response of neuron *k* according to $CP_{k}=P(r_{k}^{+}>r_{k}^{-})$ where $r_{k}^{\pm }∼P(r_{k}|sgn(\hat{s})=\pm 1)$ is a response $r_{k}^{\pm }$ of neuron *k* when the animal chooses ±1. Therefore we first transformed the measured choice probabilities to choice correlations using a known relation[14] before further analyses (Methods). Equivalently, one could measure the correlation $Corr(r_{k},sgn(\hat{s})|s=s_{0})$ between the neural response and the binary choice, which [15] showed is ≈ 0.8*C*_*k*_. Note that the above definition gives choice correlations that are either positive or negative depending on whether a rightward choice is associated with an increase or decrease in neuronal response. Therefore, we adjusted Eq (4) to generate predictions for optimal CCs that accounted for our convention (see **Methods**).

**Fig 3:**
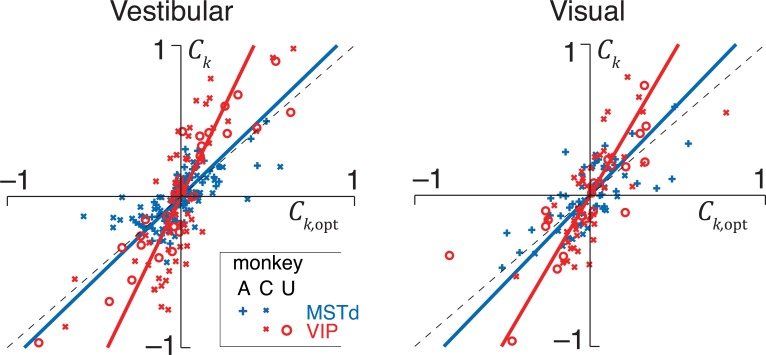
Readout is not optimal. Whereas the experimentally measured choice correlations (*C*_*k*_) of neurons in MSTd (blue) for both the vestibular (left) and the visual (right) condition are well described by the optimal predictions (*C*_*k*,opt_), those of VIP neurons are systematically greater (red). This observation was consistent across all monkeys (see **S5A Fig** for monkey *X*). Solid lines correspond to the best linear fit. Vestibular data replotted from Ref.[15] with different sign convention (see Methods).

**Fig 3**compares experimentally measured CCs against the CCs predicted by optimal decoding for all neurons recorded in the vestibular (left panel) and visual (right panel) conditions (see **S6 Fig** for data from individual animals). Our data are consistent with optimal decoding of MSTd, since the predicted and measured CCs are significantly correlated (vestibular: Pearson’s *r* =0.65, *p*<10^–3^; visual: *r* =0.70, *p*<10^–3^) with a slope not significantly different from 1 (vestibular: slope = 1.11, 95% confidence interval (CI) =[0.83 1.54]; visual: slope = 1.24, 95% CI =[0.94 1.78]). For VIP, although the predicted and measured CCs are again strongly correlated (vestibular: *r* = 0.80, *p*<10^–3^; visual: *r* = 0.75, *p*<10^–3^), the regression slope deviates substantially from unity (vestibular: slope=2.37, 95% CI =[1.97 3.08]; visual: slope=1.98, 95% CI =[1.41 2.74]), demonstrating that our data are inconsistent with optimal decoding. Note that, if VIP is decoded suboptimally, this implies that the overall decoding—one based on both VIP and MSTd—is suboptimal as well because the decoder failed to use all information available in the neurons across both populations. This leads to two questions: First, how much information is lost by suboptimal decoding? Second, how is this information lost? To get precise answers, we will now determine how the brain weights activity in MSTd and VIP to perform heading discrimination.

#### Inferring readout weights

Throughout this section, we use subscripts *M* and *V* to denote MSTd and VIP instead of the generic subscripts *x* and *y* used to describe the methods. For clarity, we will restrict our focus to the vestibular condition but results for the visual condition are presented in the supporting information. In order to determine decoding weights, we constructed two kinds of covariance structures that implied either extensive or limited information as explained earlier.

In the extensive information case, we modeled noise covariance using data from pairwise recordings within MSTd and VIP reported previously [21,29]. Those experiments established that noise correlation between neurons in these areas tends to increase linearly with the similarity of their tuning functions, or signal correlation (**Eq (8)**). This relationship between noise and signal correlations has a substantially steeper slope in VIP than in MSTd (MSTd: *m*_*M*_ = 0.19±0.08; VIP: *m*_*V*_ = 0.70±0.16, **S7 Fig**). We used these empirical relationships to extrapolate noise correlations between all pairs of independently recorded neurons within each of the two populations, using only their tuning curves, and assuming that any stimulus-dependent changes in correlation were negligible. Although the neural sensitivities were comparable in the two brain areas, the stronger correlations in VIP gave it higher information content than MSTd: since the dominant noise modes point away from the signal direction, greater correlations lead to less noise variance along the signal direction, and hence more information [35]. Since correlations between VIP and MSTd populations were not measured experimentally, we explored different correlation matrices (see **Methods**, **Eq (24)**).

In the limited information case, we added correlations that limited the total information content across the two populations (**Eq (13)**). For this latter case, we relied on behavioural thresholds before and after inactivation, and choice correlations, to determine the magnitudes of noise within (*ε*_*MM*_ and *ε*_*VV*_) and between (*ε*_*MV*_) areas (see **Methods**). In both cases, we constructed covariances for many different population sizes *N* by sampling equal numbers of neurons from both areas with replacement. The choice of distributing neurons equally among the two areas was made only for convenience and has no bearing on the result as explained later.

**Fig 4:**
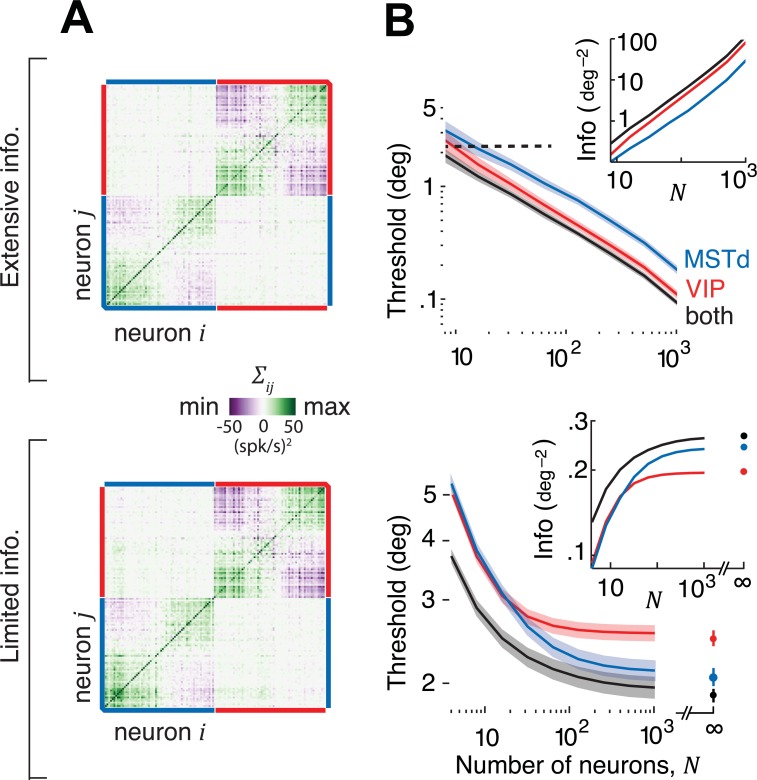
Covariance structure of extensive and limited information models. **(A)** Matrix of covariances *Σ*_*ij*_ among neurons in MSTd and VIP (*N*=128). Top: Extensive information model constructed by sampling according to the empirical relationship in **S7 Fig**, for the case when the two areas are uncorrelated on average. Bottom: Limited information model adds a small amount of information-limiting correlations with magnitudes (*ε*_*MM*_ = 4.2, *ε*_*VV*_ = 7, *ε*_*MV*_ = 0) chosen arbitrarily for illustration. **(B)** Inset shows the effect of population size on the information content implied by the two kinds of noise in MSTd (blue), VIP (red) and in both areas together (black). If decoded optimally, behavioural thresholds implied by the extensive information model would decrease with *N* resulting in performance levels that are vastly superior to those actually observed in monkeys (black dashed line). Information-limiting correlations cause information to saturate with *N*.

**Fig 4A** shows example covariance matrices for both extensive and limited information models for a population of 128 neurons. The two structures look visually similar because the additional fluctuations caused by information-limiting correlations are quite subtle. Nevertheless, there is a huge difference between the two models in terms of their information content (**Fig 4B**). The extensive model has information that grows linearly with *N*, implying that these brain areas have enough information to support behavioural thresholds that are orders of magnitude better than what is typically observed. However, when information-limiting correlations are added, information saturates rapidly suggesting that behavioural thresholds may not be much lower than population thresholds even if the decoding weights are fine-tuned for best performance. We will now infer scaling factors *a*_*M*_ and *a*_*V*_ of decoding weights using both noise models and examine their implications.

#### Extensive information model

**Fig 5:**
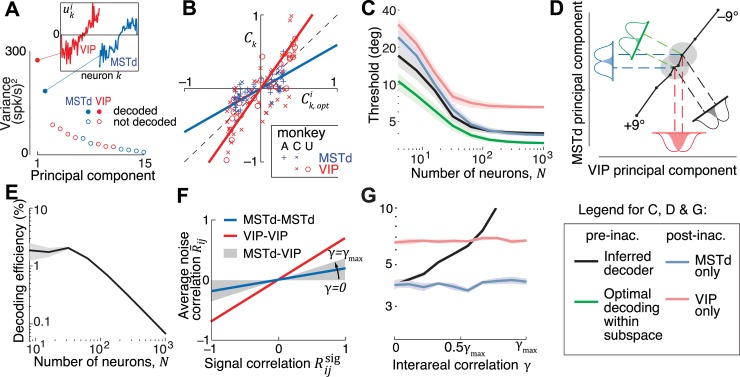
Decoder inferred using the extensive information model. **(A)** Decoding weights were inferred in the subspace of 2 leading principal components of noise covariance (solid circles). Inset: These components lie entirely within the space spanned by neurons in one of the two brain regions. Components are color coded according to the brain region that it inhabits (red=VIP; blue=MSTd). **(B)** Experimentally measured choice correlations (*C*_*k*_) of individual neurons in VIP (red) and MSTd (blue) are plotted against their respective components $C_{k,opt}^{1}$ and $C_{k,opt}^{2}$ of choice correlations generated from optimally decoding responses within the subspace of 2 leading principal components. **(C)** Unlike the optimal decoder in **Fig 4B**, the behavioural threshold predicted by the inferred weights (black) saturates at a population size of about 100 neurons. The green line indicates the performance of an optimal decoder within the two-dimensional subspace. Inactivating VIP is correctly predicted to have no effect on behavioural performance for large *N* (blue), while MSTd inactivation increases the threshold (red). **(D)** A schematic of the inferred decoding solution projected onto the first principal component of noise in VIP and MSTd. The solid colored lines correspond to the readout directions for the four cases shown in (c). The long diagonal black line is the projection of the mean population responses for headings from –9° to +9°, and the two gray ellipses correspond to the noise distribution at heading directions of ±2°. The colored gaussians correspond to the projections of this signal and noise onto each of the four readout directions, and the overlap between these gaussians corresponds to the probability of discrimination errors. **(E)** The percentage of available information read out by the inferred decoder (the decoding efficiency) decreases with population size, because the decoded information saturates while the total information is extensive. **(F)** Correlations between MSTd and VIP were not measured experimentally. We modeled these correlations according to the same linear trend that on average described correlations within each population, but with different slopes, yielding different interareal correlations parametrized by *γ* = *ε*_*MV*_/*ε*_*MM*_ (**Methods**). This slope reaches its maximum allowable value $\gamma_{max}=\sqrt{\varepsilon_{VV}/\varepsilon_{MM}}$, the geometric mean of the slopes for MSTd and VIP. **(G)** For each value of *γ*, we used the resultant covariance and CCs to infer the decoder, and plotted its behavioural thresholds. Thresholds are shown for a population of 256 neurons, by which point the performance had saturated to its asymptotic value for all *γ*. Shaded regions in (c), (e), and (g) represent ±1 SEM.

We’ve already seen that the pattern of choice correlations is not consistent with optimal decoding of MSTd and VIP. In fact, for the extensive information model, optimal decoding will lead to extremely small CCs by suppressing response components that lie along the leading noise modes as they have very little information (S8A Fig). Ironically, the magnitude of CCs found in our data could only have emerged if the response fluctuations along those leading modes substantially influenced animal’s choice (S8B Fig). This means that the decoder must be largely confined to the subspace spanned by those modes. We therefore restricted our focus to the two leading eigenvectors **u**^1^ and **u**^2^ of the covariance matrix. When the two populations are uncorrelated, these vectors lie exclusively within the one-dimensional subspaces spanned by neurons in MSTd and VIP respectively (Fig 5A). In our case, vectors **u**^1^ and **u**^2^ corresponded to **u**^*V*^ and **u**^*M*^. Although decoding only this subspace is not optimal with respect to the total information content in the two areas, a decoder could still be optimal within that subspace. To test this, we estimated the choice correlations $C_{k,opt}^{V}$ and $C_{k,opt}^{M}$ that would be expected from optimally weighting the two areas within this subspace (Eq (7)). The observed CCs were proportional (MSTd: Pearson’s *r* =0.55, *p*<10^–3^; VIP: *r* =0.76, *p*<10^–3^) to these optimal predictions implying that the leading noise modes of the extensive information model are able to capture the basic structure of choice-related activity in both areas (Fig 5B). However the slopes *β*_*M*_ and *β*_*V*_ were significantly different from 1 (*β*_*M*_ = 0.73, 95% CI =[0.63 0.84]; *β*_*V*_ = 2.38, 95% CI = [2.2 2.57]) implying that the weight scaling factors *a*_*M*_ and *a*_*V*_ must be suboptimal even within the two-dimensional subspace. Since we knew the magnitudes of *ε*_*MM*_ and *ε*_*VV*_ for this noise model from pairwise recordings (Table 1), we applied the exact rather than approximate form of Eq (20) and obtained a scaling ratio *a*_*M*_/*a*_*V*_ = 0.8 ± 0.1.

**Table 1:** Model parameters and predicted changes in CCs following inactivation for the two covariance models, shown as median ± central quartile range. (^**†**^Values correspond to when decoder is inferred using a rank-two approximation of the covariance.).

Model		Extensive information model^†^	Limited information model
**Model parameters**	Noise magnitudes	*ε*_*MM*_ = 15,*ε*_*VV*_ = 45,*ε*_*MV*_ = 0	*ε*_*MM*_ = 5,*ε*_*VV*_ = 38,*ε*_*MV*_ = 10
	Multiplicative scaling of CCs relative to optimal	*β*_*M*_ = 0.44,*β*_*V*_ = 1.4	*β*_*M*_ = 1.1,*β*_*V*_ = 2.4
	Optimal weights	|*a*_*M*_/*a*_*V*_| = 2.8 ± 0.5	|*a*_*M*_/*a*_*V*_| = 9 ± 4
	Inferred weights	|*a*_*M*_/*a*_*V*_| = 0.8 ± 0.1	|*a*_*M*_/*a*_*V*_| = 14 ± 7
**Model predictions**	Multiplicative change in CCs following inactivation	*ζ*_*M*_ = 2.2 ± 0.3	*ζ*_*M*_ = 0.9 ± 0.4
		*ζ*_*V*_ = 1.3 ± 0.1	*ζ*_*V*_ = 1.3 ± 0.4

To test whether the inferred scaling was meaningful, we compared behavioural thresholds implied by the resulting decoding scheme against experimental findings of inactivation. The threshold prior to inactivation is related to the variance of the estimator whose decoding weights **w** are along the direction specified by *a*_*M*_**u**^*M*^ + *a*_*V*_**u**^*V*^. Inactivating either area is equivalent to setting the corresponding scaling factors to zero, so post-inactivation thresholds are given by the variance along the leading noise mode specific to the active area (**u**^*M*^ or **u**^*V*^). We computed pre and post-inactivation thresholds and found they were qualitatively consistent with experimental results: for large populations, MSTd inactivation is predicted to produce a large increase in threshold (**Fig 5C**, red vs black) whereas VIP inactivation is predicted to have little or no effect (**Fig 5C**, blue vs black; see **S9 Fig** for visual condition). This correspondence to experimental inactivation results is remarkable because the procedure to deduce scaling factors *a*_*M*_ and *a*_*V*_ was not constrained in any way by behavioural data, but rather informed entirely by neuronal measurements. We also confirmed that the threshold expected from optimal scaling factors (**Table 1**) was smaller than that produced by inferred weights (**Fig 5C**, green vs black) implying that the brain indeed weighted the two areas suboptimally.

The above findings are explained graphically in **Fig 5D** by projecting the relevant quantities (tuning curves **f**(*s*), noise covariance *Σ*, decoding weights **w**) onto the subspace of the first two principal components (**u**^*M*^ and **u**^*V*^) of the noise covariance *Σ*. The colored lines indicate different readout directions, determined by the scaling (*a*_*M*_ and *a*_*V*_) of weights for the two populations. A ratio of |*a*_*M*_/*a*_*V*_| > 1 corresponds to greater weight on the estimate derived from MSTd activity, and the associated readout direction will be closer to the principal component of MSTd. The response distributions are depicted as gray ellipses (isoprobability contours) for the two stimuli to be discriminated. The discrimination threshold for different decoders can be obtained simply by projecting these ellipses onto the readout direction of the specified decoder and examining the overlap between the projections. Within this subspace, the ratio |*a*_*M*_/*a*_*V*_| of the decoder inferred from CCs was much smaller than the optimal ratio (**Table 1**), meaning that MSTd was given too little weight. Consequently, the response distributions have more overlap along the direction corresponding to the decoder inferred from neuronal CCs (black) than along the optimal direction in that subspace (green). This means that the outputs are less discriminable and thus that the decoding is suboptimal. VIP inactivation (*a*_*V*_ = 0) corresponds to decoding only from MSTd (blue). This happens to produce no deficit because the overlap of the response distributions is similar to that along the original decoder direction. On the other hand, inactivating MSTd (*a*_*M*_ = 0) corresponds to decoding only from VIP (red), where the two response distributions have greater overlap leading to a larger threshold.

It is important to keep in mind that decoding the noisiest two-dimensional subspace, which throws away all signal components in the remaining low-noise *N*–2 response dimensions, is a much more severe suboptimality than misweighting the two areas’ signals within that restricted subspace, which loses less than half the information (**Fig 5C**). As illustrated in **Fig 5E**, the efficiency — the fraction of available linear Fisher information recovered by this decoder (*η* = *J*_decoded_/*J*_opt_) — drops precipitously with the number of neurons (*η* ~ 2.5*N*^–1^). Moreover, for this model, a steeper relationship between signal and noise correlations leads to greater CCs. This is because the model is only consistent with suboptimal decoding that fails to remove the strong noise correlations; these noise correlations are decoded to drive the choice, and thus correlate neurons not only with each other but also with that choice. Thus, in the extensive information model, high CCs are a consequence of decoding a restricted subspace of neural activity, a radically suboptimal strategy for the brain.

Behavioural predictions of this model were robust to assumptions about the exact size of the decoded subspace (**S10 Fig**), but were found to depend on the magnitude of noise correlations between the VIP and MSTd populations. Since interareal correlations were not measured, we systematically varied the strength of these correlations by changing *γ* (**Fig 5F**), and used **Eq (21)** to infer scaling factors for each case. We used these scaling factors to generate behavioural predictions for different values of *γ*. Predictions for one example value of these correlations are shown in **S11 Fig**. Behavioural predictions progressively worsened as a function of the strength of noise correlations between MSTd and VIP: for this model, even weak but nonzero interareal correlations imply that inactivating area VIP should improve behavioural performance (**Fig 5G**).

#### Limited information model

In the presence of information-limiting correlations, choice correlations must be proportional to the ratio of behavioural to neuronal thresholds (Eq (17)). This was indeed the case both in MSTd and VIP as we showed already in Fig 3. Those slopes correspond to the multipliers *β*_*M*_ and *β*_*V*_ for this model, and were found to be different for the two areas (Table 1).

**Fig 6:**
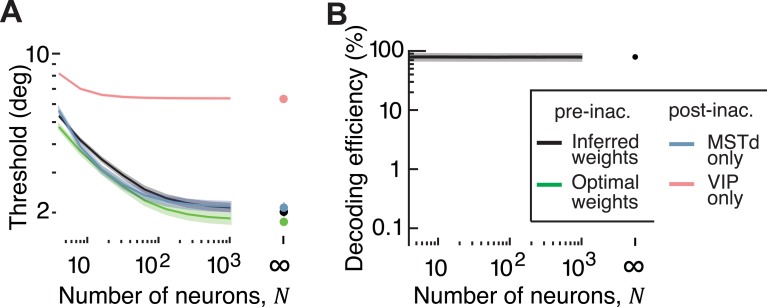
Decoder inferred using the limited information model. **(A)** Like decoding in the presence of extensive information, this decoder is suboptimal (black vs green), and can account for the behavioural effects of inactivation. **(B)** Unlike decoding in the extensive information model, the efficiency of this decoder (expressed in percentage) is high and insensitive to population size. Shaded areas represent ±1 SEM.

As we noted earlier, unlike the leading modes of noise in the extensive information model, the magnitudes of information-limiting correlations (*ε*_*MM*_, *ε*_*VV*_ and *ε*_*MV*_) are difficult to measure. Nevertheless, we can deduce them from behaviour because behavioural precision is ultimately limited by these correlations. Briefly, using behavioural thresholds *after* inactivation of each area, along with *β*_*M*_ and *β*_*V*_ derived from choice correlations as additional constraints, we can simultaneously infer the magnitude of information-limiting correlation within each area (*ε*_*MM*_ and *ε*_*VV*_), the correlated component of the noise (*ε*_*MV*_), and scaling factors (*a*_*M*_ and *a*_*V*_) (see **Methods**). A model based on these inferred parameters correctly predicted that the behavioural threshold *before* inactivation would not be significantly different from threshold following VIP inactivation (**Fig 6A;** see **S12 Fig** for visual condition). This was because the scaling of weights in MSTd was much larger than in VIP according to this model (*a*_*M*_ ≫ *a*_*V*_, **Table 1**), so inactivating VIP had little impact on the output of the decoder and left behaviour nearly unaffected. Unlike the decoder inferred for the extensive information model, the efficiency *η* of this decoder did not depend on the size of the population being decoded (**Fig 6B**, $\eta =J_{decoded}/J_{opt}=\theta_{opt}^{2}/\theta_{decoded}^{2}={\left(1.98\pm 0.06\right)}^{2}/{\left(2.2\pm 0.17\right)}^{2}=0.79\pm 0.13$) because neurons in this model carry a lot of redundant information.

#### Effect of temporal variability

**Fig 7:**
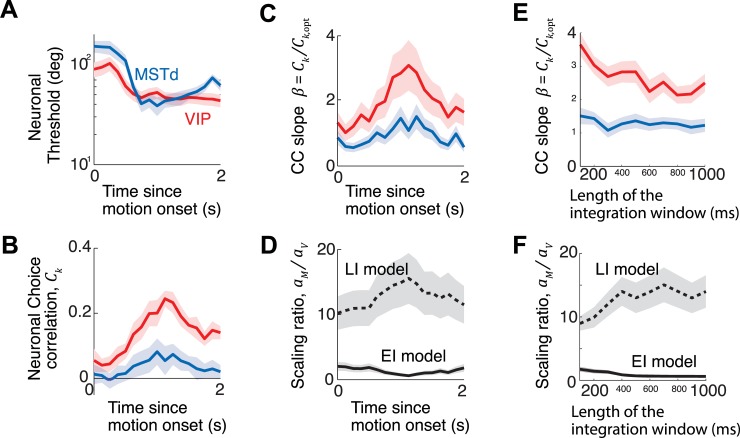
Readout weights do not vary drastically across time. Neuronal thresholds **(A)** and choice correlations **(B)** were computed for each neuron across the duration of the trial using a 250ms moving window and averaged across neurons. Note that these readouts predict the choice based only on a single time window per data point, and do not perform a weighted sum of responses in multiple windows. Neuronal thresholds in both brain areas were comparable at all times, yet the choice correlations (CCs) differed between brain areas VIP and MSTd in a consistent manner over time. Although CCs in both areas peaked around the middle of the trial, those in VIP were proportionally larger at almost all times. **(C)** Consequently the slopes, *β* = *C*_*k*_/*C*_*k*,opt_, that related observed and optimal choice correlations were generally greater in area VIP than in MSTd. **(D)** The readout weights inferred using the two models remain largely constant throughout the trial, and are qualitatively consistent with the conclusions drawn from our analyses presented in the main text: the extensive information model implies that area MSTd is underweighted, whereas the limited information model predicts the opposite. Symbols *a*_*M*_ and *a*_*V*_ denote scaling of readout weights of areas MSTd and VIP respectively. **(E)** Regression slopes are minimally affected by the length of the analysis window. Both observed neuronal choice correlations as well as those implied by optimal decoding of MSTd and VIP populations increased similarly with the length of the analysis window. This leaves the regression slopes *β* = *C*_*k*_/*C*_*k*,opt_ largely invariant with the window length for both VIP (red) and MSTd (blue). **(F)** The qualitative difference in the readout weights inferred using the two noise models are consistent across different lengths of analysis window. Error bars denote ±1 standard deviation. See **S13 Fig** for visual condition.

All analyses above were performed on neural data in the central 400ms of the trials following earlier work. This corresponds to an implicit assumption that monkeys made their decisions based solely on the information available during the period of the trial where the stimulus amplitude was highest (Gaussian stimulus profile). However, the experiments did not measure the monkeys’ psychophysical kernel, so we do not know if the above assumption is strictly valid. Moreover, both stimulus and choice-related activity typically vary across time in MSTd [23] and VIP [21], so it is unclear if our conclusions about the relative decoding weights hold outside of the time-window considered in the above analysis. To test this, we repeated our analysis using a sliding window to estimate decoding weights across time. As expected, both neuronal thresholds (Fig 7A) and choice correlations (Fig 7B) were variable across time. Transiently higher firing rates at stimulus onset provide more information early in a trial, but choice correlations peak in the middle of the stimulus. Consequently, the slopes relating observed and optimal choice correlations also varied over time in both areas (Fig 7C). Nevertheless, the time-course of the ratio of scaling factors was much less variable and the qualitative differences in the extensive and limited information models described above are still found to hold throughout the trial (Fig 7D). A full model of the time course of these signals will likely require recurrence for temporal integration (see Discussion). However, temporal integration of independent evidence would yield choice correlations that should grow monotonically with time, so the observed dynamics already indicate another form of suboptimality. Decoding weights may also depend on the length of the integration window and past studies have proposed ways to simultaneously infer the length of integration window and decoding weights from neural data [32]. Although we did not infer the size of the integration window, we found that the slopes of choice correlations in VIP were larger than MST for various choices of integration window, implying that our conclusions are robust to the duration of the analysis window (Fig 7E).

Likewise, the variance of the estimate also depends on the size of the neural recording. Although we extrapolated our data to larger populations by resampling from a set of about 100 neurons recorded from each area, our results are not attributable to the limited size of the recording (**S14 Fig**). We also extended our model to account for the fact that the two brain areas may have only been partially inactivated by Muscimol, and found that our conclusions hold under a wide range of partial inactivations (**S7 Text**; **S15 Fig**). Finally, we assumed that inactivation leaves responses in the un-inactivated area unaffected, as would be the case in a purely feedforward network model. While an exhaustive treatment of recurrent networks is beyond the scope of this work, we find that our conclusions can still hold at equilibrium if the above assumption is compromised by certain types of recurrent connections between MSTd and VIP (**S8 Text**; **S16 Fig**).

#### Comparison of the two decoding strategies

**Fig 8:**
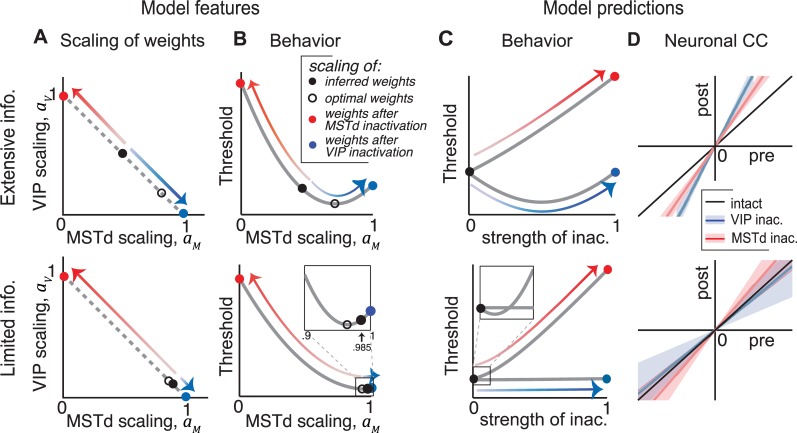
Decoding strategy and model predictions for the extensive information model and the limited information model. **(A)** Optimal (open black) and inferred (filled black) scaling of weights in MSTd (*a*_*M*_) and VIP (*a*_*V*_). Inactivation of either MSTd (red) or VIP (blue) confines the readout to the active area resulting in a scaling of 1. Red and blue arrows indicate the transformation resulting from inactivating MSTd and VIP respectively. The scaling factors always sum to 1. **(B)** Behavioural threshold *ϑ* as a function of *a*_*M*_. Whereas *ϑ* increases following MSTd inactivation for both models (red), it improves initially following partial VIP inactivation (blue) in the extensive information model (top) but remains unchanged in the limited information model (bottom). **(C)** The same curves can be replotted as a function of the strength of inactivation of MSTd (red) or VIP (blue) yielding behavioural predictions for partial inactivation of the areas. **(D)** Choice correlations (CC) of neurons in MSTd (blue) and VIP (red), before and after inactivation of VIP and MSTd respectively. Again the results following MSTd inactivation do not discriminate the two information models, but for VIP inactivation the predictions differ, showing increased CCs for the extensive information model and decreased CCs for the limited information model. Slopes of the lines correspond to *ζ*_*M*_ and *ζ*_*V*_ in **Eq (25)**, and shaded regions indicate ±1 s.d. of uncertainty.

We inferred decoding weights in the presence of two fundamentally different types of noise, the extensive information model and the limited information model. Both of these decoders could account for the behavioural effects of selectively inactivating either MSTd or VIP, albeit with very different readout schemes. For the extensive information model, neurons in area VIP were weighted more heavily than optimal, and vice-versa in the presence of information-limiting noise (Table 1, Fig 8A). Why do the two models have such different weightings? Both noise models have larger noise in VIP than MSTd, but differ in correlations between the two areas. In the extensive information model, the interareal correlations must be nearly zero to be consistent with behavioural data (Fig 5G), and the neuronal weights in VIP must be high to account for the high CCs. In the limited information model, the significant interareal correlations explain the large CCs in VIP, even with a readout mostly confined to MSTd.

How could such fundamentally different strategies lead to the same behavioural consequences? For a given noise model, an optimal decoder achieves the lowest possible behavioural threshold by scaling the weights of neurons in the two areas according to a particular optimal ratio *a*_*M*_/*a*_*V*_. Ratios that are either smaller or larger than this optimum will both result in an increase in the behavioural threshold due to suboptimality. This produces a *U-shaped* performance curve. Under certain precise conditions, complete inactivation of one of the areas will leave behavioural performance unchanged, exactly on the other side of the optimum. This is the case for VIP according to the extensive information model (**Fig 8B – top**). On the other hand, if the weight is already too small to influence behaviour then inactivation may not appreciably change performance, as demonstrated by the limited information model (**Fig 8B – bottom**).

#### Model predictions

According to the extensive information model, the brain loses almost all of its information by poorly weighting its available signals. Moreover, even beyond this poor overall decoding, the model brain gives VIP too much weight. As a consequence, this model makes a counterintuitive prediction that gradually inactivating VIP should *improve* behavioural performance! A hint of this might already be seen in Fig 2D and S5B Fig for the vestibular condition (both 0 and 12 h), although the difference was not statistically significant. Beyond a certain level of inactivation, as the weight decreases past the optimal scaling of the two areas, performance should worsen again (Fig 8C – top). According to the extensive information model, the brain just so happens to overweight VIP under normal conditions by about the same amount as it underweights VIP after inactivation. Suboptimal decoding in the limited information model has the opposite effect, giving too little weight to VIP, while overweighting MSTd. However, according to this model, the available information in VIP is small, because when MSTd is inactivated the behavioural thresholds are substantially worse (Fig 8C – bottom). Thus the suboptimality due to underweighting VIP is mild (around 80% in both visual and vestibular conditions, as described above), and the predicted improvement following partial MSTd inactivation is negligible as gradual inactivation quickly shoots past the optimum. Graded inactivation of brain areas can be accomplished by varying the concentration of muscimol, as well as the number of injections. In fact, we have previously reported that behavioural thresholds increase gradually depending on the extent of inactivation of area MSTd [22]. Unfortunately, those results do not distinguish the two models, as there is no qualitative difference between the model predictions for partial MSTd inactivation (Fig 8C, red). Future experiments involving graded inactivation of VIP should be able to distinguish between the models due to the stark difference in their behavioural predictions.

The decoding strategies implied by the two models also have different consequences for how CCs should change during inactivation experiments (**Methods, Eq (25)**). According to the extensive information model, VIP and MSTd are nearly independent, and both are decoded, so inactivating either area must scale up neuronal CCs in the other area (**Fig 8D – top**). In the limited information model, inactivating either area produces no significant changes in the other’s CCs (**Fig 8D – bottom**). This effect has different origins for MSTd and VIP. Although inactivating MSTd confines the readout to VIP, it also eliminates the high-variance noise components that VIP shared with MSTd: these two effects approximately cancel leaving CCs in VIP essentially unaffected. The results of VIP inactivation are simpler to understand: CCs in MSTd do not change much because VIP has little influence on behaviour to begin with.

## Discussion

Several recent experiments show that silencing brain areas with high decision-related activity does not necessarily affect decision-making[16–19]. To explain these puzzling results, we have developed a general, unified decoding framework to synthesize outcomes of experiments that measure decision-related activity in individual neurons and those that measure behavioural effects of inactivating entire brain areas. We know from the influential work of Haefner et al[14] how the behavioural impact (*readout weights*) of single neurons relates to their decision-related activity (*choice correlations*) in a standard feedforward network. We built on this theoretical foundation by adding three new elements that helped us relate the influence of multiple brain areas to both the magnitude of choice correlations, and the behavioural effects of inactivating those areas.

First, we have generalised their readout scheme to include multiple correlated brain areas by formulating the output of the decoder as a weighted sum of estimates derived from decoding responses of individual areas. In this scheme, the weight scales of individual estimates can be readily identified as the scaling of neuronal weights in the corresponding areas, providing a way to quantify the relative contribution of different brain areas. Second, we *postulated* that readout weights are mostly confined to a low-dimensional subspace of neural response that carries the highest response covariance, in both the extensive and limited information models. This postulate was instrumental to developing a theory of decoding that focused on the relationship between the overall scales of choice-related activity and neuronal weights, in lieu of their fine structures. Besides its mathematical simplicity, the resulting coarse-grained formulation confers an important practical advantage in that we can apply it without precisely knowing the fine structure of response covariance. Third, we used a straight-forward relation between behavioural threshold and the variance of the decoder to explicitly link the relative scaling of weights across areas to the behavioural effects of inactivating them.

Our theoretical result linking the behavioural influence of brain areas to their CCs and inactivation effects (**Eqs (20)** and **(21)**) is applicable only when neuronal weights within each area are mostly confined to the leading dimension of their response covariance. Although this requirement looks stringent, it is needed to explain the high CCs seen in experiments[15]. This claim might appear to be at odds with the fact that some earlier studies successfully predicted CCs that plateaued close to experimental levels using pooling models that did not explicitly take care of the above confinement[6,9]. However, a closer examination revealed that these studies used a scheme in which each decision was based on the average response of neuronal pools that were all uniformly correlated, a combination of model assumptions that in fact satisfies our requirement. Similar explanations apply to other simulation studies that used support-vector machines or alternative schemes that inadvertently restricted decoding weights to low-frequency modes of population response where shared variability was highest[12,30]. Thus our postulate is fully compatible with earlier work and in fact points to a more general class of models that can be used to describe the magnitude of CCs in those data.

Recent experiments show that reversibly inactivating area VIP in macaque monkeys does not impair animals’ heading perception, despite the fact that responses of VIP neurons are strongly predictive of perceptual decisions[18,21]. In contrast, inactivating MSTd does adversely affect behaviour even though MSTd neurons exhibit much weaker correlations with choice[22,23]. Assuming that both areas contribute to decisions, we used our framework to infer decoding strategies that could account for these experimental results. Surprisingly, the data were consistent with two different schemes – *overweighting* or *underweighting* of VIP – depending on whether information was *extensive* or *limited*. A major implication of the finding from the extensive information model is that if a causal test of function (e.g., inactivation) reveals no impairments, it does not disprove that a brain area contributes to a task. The limited information model on the other hand suggests that area VIP is indeed of very little use to heading perception. In spite of this difference, both models share a basic attribute, namely, that decoding is suboptimal (although to very different extents, as discussed in the next section). Therefore, our analysis reveals that the observed discrepancy between decision-related activity and effects of inactivation is not peculiar, and is actually expected from systems that integrate information across brain areas in a suboptimal fashion. The nature of this suboptimality can be understood intuitively by drawing an analogy to cue combination. Imagine there are two cues *x* and *y*, and you use a suboptimal strategy in which a larger weight is allocated to the less reliable cue *y*. If *y* is removed thereby forcing you to rely completely on *x*, then your behavioural precision might not change very much if the reduction in information from losing *y* is offset by the gain in information from *x*. On the other hand, if you mostly ignored *y* to begin with, then once again you will be unaffected by its removal. Either “too much” or “too little” weighting of a brain area can lead to suboptimal performance, both in a way that leaves the behavioural threshold largely unaltered following complete inactivation of that area.

### Decoding is suboptimal, but just how bad?

Although both models were suboptimal to some degree, the overwhelming distinction between them is the efficiency they imply for neural computation, where efficiency is the ratio of decoded information to available information. The efficiency of the limited information model is around 80%, independent of population size *N*. In contrast, the extensive information model encodes information that grows with *N*, while decoding is restricted to the least informative dimensions of neural responses. These decoders extract only a tiny fraction of the available information, resulting in an efficiency that falls inversely with *N*. For a modest-sized population of 1000 neurons, the efficiency is already less than 1%. Thus, the conventional model of correlated noise (with extensive information) is radically suboptimal, whereas the limited information model extracts an impressive fraction of what is possible, limited largely by noise.

It has previously been argued that the key factor that limits behavioural performance in complex tasks is suboptimal processing, not noise[39]. However, in simple tasks involving binary choices, and in areas in which most of the available information can be linearly decoded, it is unclear why the behaviour of highly trained animals should be so severely undermined by suboptimality. Moreover, radical suboptimality of the kind described here for the extensive information model implies tremendous potential for learning, as the neural circuits can continually optimize the computation by tuning the readout to more informative dimensions. This is hard to reconcile with the observation that behavioural thresholds in a variety of perceptual tasks typically saturate within a few weeks of training in both humans and monkeys[29,40–42]. In the presence of information-limiting noise, however, learning can only do so much, and performance must saturate at or below the ideal performance. Therefore, we regard the limited information model as a much more likely explanation of our data, for otherwise one would need to posit that cortical computations discard the vast majority of available information. Note that suboptimal cortical computation might still account for information loss in the limited information model, as opposed to neural noise[39], but this information loss is now much more modest, probably around 20%.

A direct way to tell the two models apart would be to measure the structure of noise correlations. Unfortunately, this is not straightforward, because the differences between noise models giving extensive or limited information can be quite subtle[20]. In fact, there can be a whole spectrum of subtly different noise models with different information contents, lying between the two models that we have considered here. Therefore, a more accurate technique to determine the information content (which, after all, is a major reason why we care about noise correlations) is simply to record from hundreds of neurons simultaneously, and then decode the stimulus. This will provide a lower bound on the information available in the neural population. One can then compare the resultant population thresholds with the behavioural threshold to determine how suboptimal the decoding needs to be to account for behaviour. Eventually, we expect this strategy will be successful, but it will require advances in recording technology to be viable in the target brain areas. Meanwhile, by examining the key properties of the decoding strategy implied by the two models, we identified distinct predictions that are testable without large-scale simultaneous recordings. Specifically, they involve fairly simple experiments such as graded inactivation of VIP, and measurement of CCs in either VIP or MSTd while the other area is inactivated (**Fig 8**). Future experiments will test each of these predictions to provide novel evidence about the information content and decoding strategy used by the brain.

### Limitations of the framework and possible extensions

Similar efforts to deal with outcomes of correlational and causal studies using a coherent framework are rarely undertaken, despite their significance. To our knowledge, there is only one instance where this has been attempted before[43]. In that work, the authors used a recurrent network model with mutual inhibition between populations[44,45] to reconcile choice-related activity and the effect of silencing neurons. Although their study was similar to ours in spirit, their goal was different. They showed that inactivation just before a decision, when activity was highly correlated with the choice, had less impact on the behaviour than inactivation near the stimulus onset. This addresses a *temporal*, as opposed to a *spatial*, dissociation between correlation and causation, so a model with recurrent connectivity was essential to explain their findings. In contrast, we wanted to account for the discrepancies between measures of correlation and causation across brain areas. This latter phenomenon is entirely within the realm of standard feedforward network models in which both populations causally contribute, rather than compete to drive behaviour, and differ only in terms of the relative strength of their contributions.

Time-varying weights have been shown to better predict animals’ choice in certain tasks[46], and psychophysical kernels are sometimes skewed towards one end of the trial[47,48], suggesting that decoding could also be suboptimal in time. Consistent with suboptimal integration, choice correlations in our task peak before the end of the trial, even though new evidence is still available (**Fig 7B**). Such temporal weighting of information would naturally arise from recurrent connectivity, which is beyond the scope of this work. But it can also originate in feedforward networks, possibly through a gating mechanism that blocks the integration of neural responses beyond a certain time.[32]

Other studies have considered that choice-related activity might arise from decision feedback[47,49,50]. Indeed, pure decision feedback to an area would create apparent sensitivity to sensory signals, even in the absence of direct feedforward input to the target neurons[47,49,50]. In such a case, neural sensitivity to the stimulus would then be precisely equal to the animal’s sensitivity. In the absence of other sources of variability, response fluctuations would be perfectly correlated with fluctuations in the fed-back choice, producing choice correlations of 1. Of course there would be additional variability in the neural responses, and this would dilute both the choice correlations and neural tuning by equal amounts, giving rise to measured CCs that should match the optimal CCs (**Eq (4)**). Even if there are other feedforward sensory components to the neural responses, direct decision feedback will pull the choice correlations toward this optimal prediction. Thus, simple decision feedback cannot account for the pattern of CCs observed in our VIP data, which are two to three times larger than predicted from optimal inference or direct decision feedback (**Fig 3**). Conversely, as we demonstrated through supplementary modeling, adding feedback or recurrent connections may not affect the suboptimal readout weights inferred using our scheme, even when those connections modulate responses along the decoded dimensions (**S16 Fig**). Nevertheless, future expansions of our work should account for more general recurrent connectivity to study how neural circuits simultaneously integrate information across space and time. In particular, recurrent networks also include decision feedback as a special case, and might help test alternative theories on the origins of choice correlations[1,47].

Finally, while VIP inactivation did not impair heading discrimination, MSTd inactivation partially impaired the animal’s ability to perform the task. The fact that MSTd inactivation did not completely abolish performance cannot be accounted for by our two-population models unless the inactivation was only partial and/or VIP is read out to some degree. Additionally, we cannot exclude the possibility that VIP is merely correlated with behaviour and that a third brain area besides MSTd contributes some task-relevant information. In fact, both of our models actually predict a somewhat bigger deficit following MSTd inactivation (**Figs 5C and 6A**) than is observed experimentally (**Fig 1B**). This highlights the importance of ultimately extending coding models to include more than two brain areas.

As neuroscience moves towards ‘big data’, there is a greater need for theoretical frameworks that can help discern simple rules from complex multi-neuronal activity[51]. We believe our work responds to this challenge and, despite its limitations, takes us closer to bridging the brain-behaviour gap for binary-decision tasks.

## Methods

### Ethics statement

All surgical and experimental procedures were approved by the Institutional Animal Care and Use Committees at Washington University and Baylor College of Medicine, and were performed in accordance with institutional and National Institutes of Health (NIH) guidelines.

### Relation between behavioural threshold and weight scaling factors

Behavioural threshold *ϑ* is proportional to the square root of the decoder variance (with proportionality of 1 for threshold of 68% correct), so *ϑ*^2^ = **w**^T^*Σ***w**. If decoding is confined to the subspace of leading eigenmodes **u**^*x*^ of *Σ* spanned by neurons within each population *x*, then $w_{x}=u^{x}/({f_{x}^{\prime }}^{T}u^{x})$ where the constant of proportionality ensures unbiased decoding from that population. In this case, the behavioural threshold can be expressed purely in terms of weight scaling factors and the variance originating from noise within the noise modes as (**S3 Text**):

$$\begin{array}{c} \vartheta^{2}=a^{T}Ea=a_{x}^{2}\varepsilon_{xx}+a_{y}^{2}\varepsilon_{yy}+2a_{x}a_{y}\varepsilon_{xy} \end{array}$$ (22)

where *E* = *ε*_*xy*_ is the covariance matrix of the noise decoded from populations *x* and *y*. Thresholds following inactivation can be determined by setting the weight scaling factor for the inactivated areas to zero. In the case of two populations, this yields $\vartheta_{-x}^{2}=\varepsilon_{yy}$ and $\vartheta_{-y}^{2}=\varepsilon_{xx}$.

### Subjects and behavioural task

Six adult rhesus monkeys (A, B, C, J, S, U, and X) took part in various aspects of the experiments. Three animals were employed in each of the MSTd (C, J and S) and VIP (X, B and J) inactivation experiments. Two animals provided the neural data from each brain area (A and C for MSTd; C and U for VIP). All animals were trained to perform a heading discrimination task around psychophysical threshold. In each trial, the subject experienced a real or simulated forward motion with a small leftward or rightward component (angle *s*, **Fig 1A**). Subjects were required to maintain fixation within a 2x2˚ electronic window around a head-fixed visual target located at the center of the display screen. At the end of each 2-s trial, the fixation spot disappeared, two choice targets appeared and the subject made a saccade to one of the targets to report his perceived heading relative to straight ahead. Nine logarithmically spaced heading angles were tested (0˚, ±0.5˚, ±1.3˚, ±3.5˚, and ±9˚ for monkeys A and J, 0˚, ±1˚, ±2.5˚, ±6.4˚, and ±16˚ for monkeys B, C, S and U), including the ambiguous case of straight ahead motion (*s* = 0˚). These values were chosen to obtain near-maximal psychophysical performance while allowing neuronal sensitivity to be estimated reliably for most neurons[21,23]. Subjects received a juice reward for indicating the correct choice. For trials in which the ambiguous heading was presented, rewards were delivered randomly on half of the trials. The experiment consisted of three randomly-interleaved stimulus conditions (vestibular, visual, and combined). In the vestibular condition, the monkey was translated by a motion platform while fixating a head-fixed target on a blank screen. In the visual condition, the motion platform remained stationary while optic flow simulated the same range of headings. Under the combined condition, both inertial motion and optic flow were provided. Each of the 27 unique stimulus conditions (9 heading directions × 3 cue conditions) was repeated at least 20 times, for a total of 540 discrimination trials per recording session. Identical stimuli and trial structure were employed during both neural recordings and inactivation experiments.

### Neural recordings

Activity of single neurons in areas MSTd and VIP was recorded extracellularly using epoxy-coated tungsten microelectrodes (impedance of 1–2 MΩ). Area MSTd was located using a combination of magnetic resonance imaging (MRI) scans, stereotaxic coordinates (~15 mm lateral and ~3–6 mm posterior to AP-0), white/gray matter transitions, and physiological response properties. In some penetrations, electrodes were further advanced into the retinotopically organized area MT[23]. Most recordings concentrated on the posterior/medial portions of MSTd, corresponding to more eccentric, lower hemifield receptive fields in the underlying area MT. To localize area VIP, we first identified the medial tip of the intraparietal sulcus and then moved laterally until there was no longer directionally selective visual response in the multiunit activity, as described in detail previously[21].

### Estimation of behavioural and neuronal thresholds

Behavioural performance was quantified by plotting the proportion of 'rightward' choices as a function of heading (the azimuth angle of translation relative to straight ahead). Psychometric data were fit with a cumulative Gaussian function with mean *μ* and standard deviation *ϑ*, and this standard deviation defined the psychophysical threshold, corresponding to 68% correct performance (*d*′ = 1, assuming no bias, i.e. *μ* = 0).

For the analysis of neuronal responses, we used the linear Fisher information *J* which is simply a measure of the signal-to-noise ratio: signal power divided by noise power. The linear Fisher Information captures all of the Fisher information in responses generated from the exponential family with linear sufficient statistics. Its inverse is exactly equal to the variance of an unbiased, locally optimal linear estimator (for differentiable tuning curves and nonsingular noise covariance). We defined the square root of this variance (i.e. the standard deviation of the estimator) to be the neuronal discrimination threshold, which corresponds to 68% accuracy in binary discrimination. This threshold can be obtained directly from the neuron’s tuning curve and noise variance as follows:

$$\begin{array}{c} \vartheta_{k}=\frac{1}{\sqrt{J_{k}}}=\frac{\sigma_{k}}{f_{k}^{\prime }} \end{array}$$ (23)

where *ϑ*_*k*_ and *J*_*k*_ are the threshold and linear Fisher information[52] for neuron *k*, $f_{k}^{\prime }$ is the derivative of the neuron’s tuning curve at the reference stimulus (0˚), and $\sigma_{k}^{2}$ is the variance of the neuronal response for that stimulus. Neuronal thresholds computed using the above definition were very similar to those computed using a traditional approach based on neurometric functions constructed from the responses of the recorded neuron and a presumed 'antineuron' with opposite tuning[53] (**S4 Fig**).

### Estimation of choice correlation

To quantify the relationship between neural responses and the monkey’s perceptual decisions, we first computed choice probabilities (CP) using ROC analysis[54]. For each heading, neural responses were sorted into two groups based on the choice that the animal made at the end of each trial. In previous studies, the two choice groups were typically related to the preferred and non-preferred stimuli for a given neuron[21,23]. In this study, in order to appropriately compare different neurons in a population code, the two choice groups were simply rightward and leftward choices; hence, CPs may be greater than or less than 1/2. ROC values were calculated from these response distributions, yielding a CP for each heading, as long as the monkey made at least 3 choices in favor of each direction. To combine across different headings, we computed a grand CP for each neuron by balanced *z*-scoring of responses in different conditions, which combines *z*-scored response distributions in an unbiased manner across conditions, and then performed ROC analysis on that combined distribution[55]. The CPs were then converted to choice correlations according to $C_{k}\approx \frac{\pi }{\sqrt{2}}({CP}_{k}-\frac{1}{2})$ (refs. [14,15]) where *CP*_*k*_ and *C*_*k*_ are the choice probability and choice correlation of neuron *k* respectively (**S1 Text**). Due to the convention we chose for computing CPs, the resulting choice correlation could be positive or negative depending whether a neuron predicted *rightward* choices by increasing or decreasing its response relative to reference stimulus. For an optimal decoder, the sign of a neuron’s choice correlation should match the sign of the derivative of its tuning curve, so we modified the definition of ref.[15] (**Eq (4)**) to accommodate our sign convention, yielding $C_{k,opt}=sgn(f_{k}^{\prime })\vartheta /\vartheta_{k}$ where sgn denotes the signum function.

There were neurons in both MSTd and VIP whose choice-related activity during the visual condition is anticorrelated with their signal-related activity[21,23]. Further analysis showed that heading preferences of these neurons during visual and vestibular conditions differed. Therefore the analysis of data collected during the visual condition presented in the supporting material included only the subset of recorded neurons that had similar heading preferences as in the vestibular condition[23] (MSTd: 66/129 neurons; VIP: 63/88 neurons).

### Noise covariance of extensive information model

Pairwise neuronal recordings carried out separately in areas VIP and MSTd were used to estimate noise correlations between pairs of neurons, *R*_*ij*_ = Corr(*r*_*i*_,*r*_*j*_|*s* = 0), where *r*_*i*_ and *r*_*j*_ are the responses of neurons *i* and *j*, and correlation coefficients were computed by averaging over trials with headings near 0°. The same recordings were used to compute signal correlations, $R_{ij}^{sig}=Corr(f_{i},f_{j})$, where *f*_*i*_ and *f*_*j*_ are the tuning curves of neurons *i* and *j*, and the correlation coefficients were computed by averaging over a uniform distribution of headings in the horizontal plane. The typical noise correlations, $\bar{R}$, were then modeled as linearly proportional to the signal correlations (**Eq (8)**). The slope of the relation was much steeper in VIP than MSTd[21]. For the vestibular condition, slopes were found to be *m*_*M*_ = 0.19±0.08 and *m*_*V*_ = 0.70±0.16 within MSTd and VIP respectively, and for the visual condition they were *m*_*M*_ = 0.12±0.09 and *m*_*V*_ = 0.50±0.14. The above fits determined the average relationship between noise and signal correlations, but there was considerable diversity around this trend. To emulate this diversity, we used a technique similar to the one proposed in ref. [31]. Specifically, we sampled correlation coefficient matrices *R* from a Wishart distribution with a mean matrix $\bar{R}$ given by **Eq (8)** and the fitted slope *m*, and rescaled them to ensure *R*_*ii*_ = 1. The number of degrees of freedom for the Wishart distribution was adjusted so sampled matrices had the same uncertainty in slope *m* as the data when subjected to the same fitting procedure. Covariance matrices were generated by scaling the correlation coefficients by the standard deviations for each neuron. Model variances were set equal to the mean responses, so the standard deviation of neuron *i* is *f*_*i*_^1/2^. Thus the covariance *Σ* is related to correlation coefficients *R* by $\Sigma_{ij}=R_{ij}\sqrt{f_{i}f_{j}}$. Correlations between responses of MSTd and VIP neurons were not measured experimentally, so the slope *m*_*MV*_ of any linear trend relating noise and signal correlations between the two areas was not known. We explored different possibilities by varying *m*_*MV*_ according to:

$$\begin{array}{c} m_{MV}=k\sqrt{m_{M}m_{V}} \end{array}$$ (24)

where *k* ∈ [0,1). Each value of *k* produced correlation between areas with magnitude *ε*_*MV*_ which was expressed as *ε*_*MV*_ = *γε*_*MM*_.

### Noise covariance of limited information model

If the information reaching MSTd (*M*) and VIP (*V*) is not perfectly redundant across the populations, then the resulting covariance matrix will be of the form given by **Eq (13)** where *M* and *V* take the places of *x* and *y*. The resultant covariances *ε*_*MM*_, *ε*_*VV*_, and *ε*_*MV*_ are difficult to determine even with large-scale recordings since their magnitudes may be very small compared to the magnitude of noise in *Σ*. Nevertheless, we know that for large populations, the behavioural threshold will be dominated by the magnitude of information-limiting correlations. Specifically, they are related through the relative scaling of decoding weights in **Eq (22)**. Consequently, we can determine *ε*_*MM*_ and *ε*_*VV*_ from behavioural thresholds following inactivation using $\varepsilon_{MM}=\vartheta_{-V}^{2}$ and $\varepsilon_{VV}=\vartheta_{-M}^{2}$. We can then use **Eq (22)** in conjunction with **Eq (21)** to determine both the ratio *a*_*M*_/*a*_*V*_ of scaling factors and the magnitude of correlation between populations *ε*_*MV*_ = *γε*_*MM*_.

### Effects of inactivation on choice correlations

Complete inactivation of one of the areas will affect neuronal choice correlations in the non-inactivated area. If **C**_*x*_ and ${\tilde{C}}_{x}$ denote the choice correlations of neurons in area *x* before and after inactivation of *y*, then it can be shown that ${\tilde{C}}_{x}=\zeta_{x}C_{x}$ and similarly ${\tilde{C}}_{y}=\zeta_{y}C_{y}$ where scalars *ζ*_*y*_ and *ζ*_*y*_ are (**S9 Text**):

$$\begin{array}{c} \zeta_{x}=\frac{1}{\beta_{x}}\frac{\vartheta_{-y}}{\vartheta };\;\zeta_{y}=\frac{1}{\beta_{y}}\frac{\vartheta_{-x}}{\vartheta } \end{array}$$ (25)

where *β*_*x*_ and *β*_*y*_ are the multipliers that relate the observed and optimal patterns of neuronal choice correlations in areas *x* and *y*. The above equation implies that choice correlations in the active area will increase by a factor proportional to the behavioural effect of inactivating the other area. Intuitively, this is because inactivating an area that was very important for behaviour will dramatically increase the burden on the active area, leading to an increase in the magnitude of choice-related activity.

## Supporting information

S1 FigChoice correlations decrease with the number of decoded modes.**(A)** Tuning functions *f*_*i*_(*s*) (left) and covariance matrix Σ (right) of a subset of model neurons used in this simulation. The stimulus *s* ∈ (−*π*,+*π*] was a circular variable and tuning followed a von Mises function: $f_{i}(s)=b_{i}+h_{i}e^{\kappa_{i}\cos\left(s-s_{i}\right)}$ where baseline and height *b*_*i*_ and *h*_*i*_ were drawn from Poisson distributions $b_{i}∼Poiss(\bar{b})$ and $h_{i}∼Poiss(\bar{h})$ with means $\bar{b}$ = 5 spikes/sec and $\bar{h}$ = 15 spikes/sec, tuning peakiness *κ*_*i*_ was sampled from the rectified normal distribution $\kappa_{i}∼|N(1,0.25)|$, and preferred stimulus *s*_*i*_ was drawn from a uniform distribution. Covariance Σ_*ij*_ between neurons *i* and *j* was $\Sigma_{ij}=R_{ij}\sqrt{f_{i}f_{j}}$ where noise correlation coefficient *R*_*ij*_ was proportional to signal correlation (**Eq (8)**) with a proportionality of 0.2. **(B)** Neurons were linearly decoded by confining readout weights to the leading *p* eigenmodes of the covariance. Weights were always chosen to be optimal within the decoded subspace, and *p* was varied from 1 to *N* where *N* = 512 denotes the population size. The root-mean-squared choice correlation *C*_RMS_ over all neurons decreases with *p*: for this model population, it drops by an order of magnitude already for *p* = 2. Inset shows *C*_*k*_ of each neuron for two example cases. **(C)** Choice correlations tend to decrease with population size when all modes are decoded optimally (gray: *p* = *N*), but remain insensitive to population size when only the leading mode is decoded (black: *p* = 1).(PDF)

S2 Fig Recovering the true values of the decoder scaling factors in simulated neural populations. In this demonstration, 6 populations with information-limiting noise are each manipulated by a random multiplicative inactivation factor. We successfully recover decoder scalings (left) and population noise covariance (right) using behavioural thresholds and choice correlation slopes during these inactivation experiments by numerically solving **Eqs (18)** and **(19)**.
(PDF)

S3 FigInactivation effects may not reflect relative influence of brain areas on behaviour.Consider two populations *x* and *y* with relative scaling of neuronal weights *a*_*x*_ and *a*_*y*_. These scalings depend not only on the post-inactivation thresholds (*ϑ*_−*x*_ and *ϑ*_−*y*_) but also on the magnitude of their choice correlations (*β*_*x*_ and *β*_*y*_) according to **Eqs (20)** and **(21)**. The two panels illustrate the relative choice correlation magnitudes (*β*_*x*_/*β*_*y*_, color) for uncorrelated populations (**Eq (20)**) and correlated populations (**Eq (21)**), as a function of the scaling ratio *a*_*x*_/*a*_*y*_ and the inactivation ratio ($\vartheta_{-x}^{2}/\vartheta_{-y}^{2}$). For simplicity, here we assume that *β*_*y*_ = 1, so *β*_*x*_/*β*_*y*_ = 1 corresponds to optimal decoding. (**A**) For systems in which the two populations are uncorrelated (*ε*_*xy*_ = 0), the scaling ratio *a*_*x*_/*a*_*y*_ is directly proportional to inactivation ratio $\vartheta_{-x}^{2}/\vartheta_{-y}^{2}$. Nonetheless the slope of this relationship depends on the ratio of choice correlation magnitudes *β*_*x*_/*β*_*y*_ (isochromatic contours), so a population with a larger weight could produce a smaller deficit upon inactivation, or vice-versa (black asterisks). Inactivation effects exactly match the ratio of scalings (e.g. black open circle on the main diagonal) only if decoding is optimal (black dashed line). (**B**) When the populations are correlated, the scaling ratio is no longer proportional to the inactivation ratio. Instead, their relationship is nonlinear (black dashed line), and the two ratios may not match even if decoding happens to be optimal (e.g black open circle). In other words, the change in behavioural threshold does not match how much each area is decoded. Here cross-population correlation *ε*_*xy*_ is $\sqrt{\varepsilon_{xx}\varepsilon_{yy}}/2$ for illustration.(PDF)

S4 FigDirect and conventional methods yield similar neuronal thresholds.Each neuron’s threshold was estimated in two ways—directly as the inverse square-root of its Fisher information at *s* = 0 (Methods – **Eq (23)**), or using a traditional approach by constructing a neurometric function. The latter approach used ROC analysis to compute the ability of an ideal observer to discriminate between two oppositely-directed headings (e.g., –6.4° vs. +6.4°) based solely on the firing rate of the recorded neuron and a presumed 'antineuron' with opposite tuning[1]. ROC values were plotted as a function of heading, resulting in neurometric functions that were fit with a cumulative Gaussian function. Neuronal threshold was then defined as the standard deviation of the fitted Gaussian, but increased by a factor of $\sqrt{2}$ to adjust for the extra information from the antineuron. This $\sqrt{2}$ adjustment arises because a decision based on a neuron-antineuron pair has twice the signal amplitude but also twice the noise variance, compared to a single neuron and a fixed, noiseless 0° reference. Note that this factor of $\sqrt{2}$ differs from past studies[2] that assumed a noisy 0° reference heading and thus corrected by a factor of 2. (**A**) The two methods yielded very similar estimates for vestibular thresholds across neurons in both MSTd (blue, Pearson’s correlation *r* = 0.55, *p* = 4 × 10^−11^) and VIP (red, *r* = 0.31, *p* = 5 × 10^−3^). (**B**) Similar results were found for visual thresholds: MSTd (blue, *r* = 0.65, *p* = 3 × 10^−9^) and VIP (red, *r* = 0.87, *p* = 1 × 10^−20^). For these comparisons, we omitted a small subset of insensitive neurons (Vestibular: 4/129 MSTd neurons and 7/88 VIP neurons, Visual: 1/129 MSTd neurons and 5/88 VIP neurons) with extremely large thresholds (>300°).(PDF)

S5 Fig **(A) Choice correlations of VIP neurons.** Neural recordings were carried out in a separate monkey X prior to inactivation of area VIP, while he performed a heading discrimination task whose structure was identical to that described in Methods in all regards, except each trial lasted only 1s instead of 2s. Similar to those in monkeys C and U, neuronal choice correlations in area VIP are proportional to but greater than those expected from optimal decoding of these neurons during both vestibular (top) and visual (bottom) heading discrimination tasks. The 95% CI of slopes *β*_*V*_ were found to be [1.9 2.9] and [1.2 1.8] for the vestibular and visual conditions respectively. **(B) Behavioural effects of VIP inactivation.** *Left*: Discrimination thresholds at different times (different shades of blue) following inactivation of VIP, for all seven experiments conducted on monkey X. Thresholds obtained in a single experimental session are connected by a line. Across experiments, inactivating area VIP failed to elicit significant changes in either the vestibular or visual conditions. The behaviour of this monkey was tested 36 hours following inactivation in only 3 of the 7 experiments. *Right*: Psychometric functions at different times during inactivation of area VIP, averaged across experiments, for the vestibular (top) and visual (bottom) conditions. Behavioural thresholds computed from the psychometric functions at different times are shown in the bottom panels. None of the comparisons were significant (Wilcoxon rank-sum test, significance-level of *p* = 0.05). Error bars indicate standard error of the mean.
(PDF)

S6 Fig Pattern of choice correlations in individual animals. Experimentally measured choice correlations (*C*_*k*_) of neurons in MSTd (blue) for both the vestibular (top) and the visual (bottom) condition are close to optimal predictions (*C*_*k*,opt_), those of VIP neurons are systematically greater (red). This observation holds individually in each monkey. Solid black lines correspond to the best linear fit. Vestibular data in monkeys C and U are replotted from Ref.[15] with different sign convention (see **Methods**). Monkey A was used only for MSTd recordings, and monkeys U and X only for VIP.
(PDF)

S7 FigNoise and signal correlations.Pairs of neurons within MSTd (blue; *n*=127 pairs) and VIP (red; *n*=139 pairs) were recorded when the animal experienced self-motion in various directions based on either vestibular (left) or visual (right) cues. For each pair of neurons *i* and *j*, correlated variability in the firing rates across trials (*noise correlation* $R_{ij}^{noise}$) is plotted against correlated variability in the average firing rates across stimuli (*signal correlation* $R_{ij}^{sig}$). The relationship between signal and noise correlation was fit to a linear model (**Eq (8)**) separately for each area, represented here using straight lines. Shaded areas correspond to 95% confidence intervals of the resulting fits.(PDF)

S8 FigNoise along the leading modes of covariance substantially influences choice.Any readout weight **w** can be expressed as a linear combination of the eigenvectors **u**_*p*_ of the response covariance as **w** ∝ ∑_*p*_*a*_*p*_**u**_*p*_ where the constant of proportionality is chosen to ensure unbiased decoding. Coefficients magnitudes |*a*_*p*_| indicate how much the different eigenmodes *p* contribute to behavioural choice. To assess the specific contribution of the leading mode from MSTd and VIP, we considered three different cases: optimal decoding of response along all available modes, a decoder confined to the leading eigenmode in each area, and a spectrum of decoders in between the two extremes. We decoded MSTd & VIP responses separately in all cases using covariance Σ specified by the extensive information model (**Fig 4A** – *left*), and examined the average magnitude of choice correlations across all neurons in each case. **(A) Optimal decoding of all modes.** The pattern of coefficients *a*_*p*_ of the optimal decoder of MSTd (blue) and VIP (red) responses. For clarity, only the coefficients corresponding to the leading 40 modes are shown. Evidently, the leading mode has little influence on the decoder output as seen from the magnitude of coefficient *a*_1_. **(B)** The average choice correlation, quantified as the root-mean squared (RMS) choice correlations of the set of all neurons, decreases to ~0.01 even for the modest population size of *N* = 1000 neurons. **(C,D) Decoding leading mode only.** Plotted as for A,B, but restricting the readout to one leading eigenmode. We forced the coefficients *a*_*p*_ to zero for all *p* ≠ 1, yielding **w** ∝ **u**_1_. Choice correlations implied by this decoder asymptote to about 0.2 and 0.4 for MSTd and VIP, values that are of the same order of magnitude as seen in the experiments. **(E) Varying weight on leading mode.** We tested whether the leading mode must contribute substantially to choice, in order to generate high choice correlations. To test this, we first parametrised the contribution of the leading mode as the fraction *Φ* of weight power it contributes to decoding, according to $\Phi =a_{1}^{2}/\sum_{p=1}^{N}a_{p}^{2}$. To control *Φ*, we simply manipulated the coefficients *a*_*p*_ of the optimal decoder, first by setting the leading coefficient ${\tilde{a}}_{1}$ to $\sqrt{\Phi }$ and then rescaling all the remaining coefficients together so ${\tilde{a}}_{p}=a_{p}\sqrt{(1-\Phi )/\sum_{p=2}^{N}a_{p}^{2}}$. Weights obtained by this procedure resemble the optimal weight pattern except for the differences arising from the leading mode. We then systematically varied *Φ* from 1/*N* to 1 where the number of neurons *N* was fixed to 1024 in this simulation. Choice correlations increase slowly with *Φ*, and reach half-max at about *Φ* = 0.25 (dashed vertical line). **(F)** Influence of the leading mode on noise in the output increases much more rapidly with *Φ* than choice correlations do. For each value of *Φ*, we computed the fraction *ξ* of total noise variance that comes from the leading mode as $\xi ={\tilde{a}}_{1}^{2}\lambda_{1}/\sum_{p=1}^{N}{\tilde{a}}_{p}^{2}\lambda_{p}$ where *λ*_*p*_ denotes the eigenvalue of the *p*^th^ mode. At *Φ* = 0.25, more than 95% of noise propagated to the output is inherited exclusively from this mode (dashed vertical line).(PDF)

S9 FigDecoder inferred using the extensive information model – visual condition (compare to Fig 5B and 5C).**(A)** Experimentally measured choice correlations (*C*_*k*_) of individual neurons in MSTd (blue) and VIP (red) are plotted against the *i*^th^ component $C_{k,opt}^{i}$ of choice correlations generated from optimally decoding the responses within the subspace of two leading principal components of noise covariance. When two populations are not correlated with each other, the two leading components of the global noise covariance correspond to the largest noise modes in each population separately. Consequently $C_{k,opt}^{1}$ and $C_{k,opt}^{2}$ correspond to optimal choice correlations in VIP and MSTd, respectively. **(B)** Performance (threshold) of a decoder with weights inferred from the subspace of two leading principal components of the noise covariance. The black and green lines indicate the performance of the inferred and optimal decoders within this subspace. Inactivating VIP is correctly predicted to have no effect on behavioural performance (blue), while MSTd inactivation increases the threshold (red). Shaded region indicates ±1 SEM.(PDF)

S10 Fig Effect of the decoded subspace dimensionality on performance of the decoder inferred from choice correlations using the extensive information model. Since decoding performance was nearly saturated at 256 neurons (**Fig 5C**), we fixed the size of the neural population at *N* = 256, and examined the behavioural threshold when varying the dimensionality of the decoded subspace. Decoding weights were inferred in the subspace spanned by a total of *p* eigenvectors of the covariance matrix, using *p*/2 eigenvectors in both MSTd and VIP. The decoder continued to correctly predict the qualitative effects of inactivating MSTd and VIP beyond the 2-dimensional subspace considered in **Fig 5**, roughly until about *p* = 22 (vertical dashed line). Note that the threshold predicted by the optimal decoder within the restricted subspace (green) improves as more (informative) dimensions are included, while that of the inferred decoder worsens. Therefore, readout weights extract more noise than signal from these additional dimensions. This makes sense because if it the weights were instead tuned to decrease the variance in the estimate as more dimensions are added, they would no longer explain the large measured choice correlations. One reason why the experimental predictions of this model break down for large *p* is that the predictions are only reliable in the regime of small *p* where the effect of measurement noise is low. This is because the reliability of inferred decoding weights (and consequently also its predictions) is inversely related to the eigenvalue of the decoded mode, so reliability of the predictions worsens as *p* increases (**S6 Text**).
(PDF)

S11 FigEffect of interareal correlations on decoder inferred from choice correlations using the extensive information model.*Left*: A representative covariance matrix when neurons in MSTd and VIP are mildly correlated through the leading noise modes ($\varepsilon_{xy}\approx 0.2\sqrt{\varepsilon_{xx}\varepsilon_{yy}}$). *Right*: In contrast to the observed effects of inactivation, the decoder inferred using the covariance on the left incorrectly predicted that inactivating VIP should reduce the behavioural threshold. This was unlike the decoder shown in **Fig 5C** that correctly predicted the effects of VIP inactivation when correlations between the two areas were zero on average.(PDF)

S12 Fig Decoder inferred using the limited information model: visual condition. **(A)** Like decoding in the presence of extensive information, this decoder is suboptimal (black vs green), and can account for the behavioural effects of inactivation. **(B)** Unlike decoding in the extensive information model, the efficiency of this decoder is quite high and insensitive to population size. Shaded areas represent ±1 SEM.
(PDF)

S13 Fig Readout weights for the visual condition do not vary drastically across time. Neuronal thresholds **(A)** and choice correlations **(B)** were computed for each neuron across the duration of the trial using a 250ms moving window and averaged across neurons. Note that these readouts predict the choice based only on a single time window per data point, and do not perform a weighted sum of responses in multiple windows. Neuronal thresholds in both brain areas were comparable at all times, yet the choice correlations (CCs) differed between brain areas VIP and MSTd in a consistent manner over time. Although CCs in both areas peaked around the middle of the trial, those in VIP were proportionally larger at almost all times. **(C)** Consequently the slopes, *β* = *C*_*k*_/*C*_*k*,opt_, that related observed and optimal choice correlations were generally greater in area VIP than in MSTd. **(D)** The readout weights inferred using the two models remain largely constant throughout the trial, and are qualitatively consistent with the conclusions drawn from our analyses presented in the main text: the extensive information model implies that area MSTd is underweighted, whereas the limited information model predicts the opposite. Symbols *a*_*M*_ and *a*_*V*_ denote scaling of readout weights of areas MSTd and VIP respectively. **(E)** Regression slopes are minimally affected by the length of the analysis window. Both observed neuronal choice correlations as well as those implied by optimal decoding of MSTd and VIP populations increased similarly with the length of the analysis window, leaving the regression slopes *β* = *C*_*k*_/*C*_*k*,opt_ largely invariant with the window length for both VIP (red) and MSTd (blue). **(F)** The qualitative difference in the readout weights inferred using the two noise models are consistent across different lengths of analysis window. Error bars denote ±1 standard deviation.
(PDF)

S14 Fig Threshold saturation effects are not influenced by size of the dataset. In the main text, we presented thresholds predicted by decoders inferred using the Extensive information (EI) (**Fig 5C**) and Limited information (LI) (**Fig 6B**) models. These thresholds were generated by extrapolating a limited dataset containing 129 and 88 neurons from MSTd and VIP respectively. However, those thresholds approached saturation only around 60-70 raising the possibility that those results might be sensitive to the exact number of neurons that were used for extrapolation. To test whether this was the case, we repeated all our analyses by considering only a fraction of the recorded neurons for extrapolation. **(A)** *Left*: Thresholds implied by the EI model obtained by extrapolating 50% of the neurons in our dataset (*n*=65/129 and 44/88 neurons in MSTd and VIP). Thresholds were found to asymptote to nearly the same value obtained by extrapolating the full dataset (compare with **Fig 5C**). *Right*: We repeated this procedure for different percentages (10%–100%) and found that our results can be reproduced with as little as 30% of the dataset. The asymptotic thresholds (evaluated at a population size of *N* = 1024 neurons) do not change much beyond this point (shaded region). **(B)** Thresholds implied by the LI model obtained by extrapolating 50% of the dataset. Once again, this was similar to results obtained using the full dataset (**Fig 6B)**.
(PDF)

S15 Fig Inferred readout strategy is robust to the degree of inactivation. We extended our model to include two additional parameters *ρ*_*x*_ and *ρ*_*y*_ that denote fractions of neurons inactivated in populations *x* and *y*, and derived theoretical results that account for partial inactivation of the two populations (**S7 Text**). We used those results to model partial inactivation of the MSTd and VIP in our dataset, and computed parameter ranges in the (*ρ*_*M*_,*ρ*_*V*_) parameter space (shaded areas) that are consistent with 95% confidence intervals around experimental data. (**A**) *Extensive information model*. Since an empirical trend between neural tuning and noise covariance was used to determine the structure of noise correlations, the readout weights could be uniquely determined from the observed pattern of choice correlations (CCs) independent of the extent of inactivation. Therefore the inferred readout weights remained the same as for the model that assumed complete inactivation (inferred MSTd weight scaling *a*_*M*_ = 0.44; optimal MSTd weight scaling *a*_*M*_ = 0.74). Nonetheless, the predictions for behavioural thresholds following inactivation of MSTd or VIP (shown in **Fig 5B**) are quantitatively consistent with the experimental observations (**Fig 2B**) only for a specific range of inactivation fractions (grey region). Specifically, the inferred readout weights predict that the thresholds should increase by a factor of 1.6 if MSTd was fully removed, yet the observed increase was only 1.2±0.1. This suggests that MSTd could neither have been completely inactivated nor remained completely intact, leading to the exclusion of the regions close to the left and right boundaries. For the EI model, therefore, partial inactivation of MSTd was a better match to the behavioural data. Similarly, inactivating about half of VIP is predicted to significantly reduce the threshold (**Fig 8C** – top panel). Since this was not observed experimentally, the inactivation parameters within the central horizontal band around 0.5 are excluded from the grey region that is consistent with data. Even with partial inactivation, therefore, the extensive information model implies that the brain underweights MSTd compared to optimal, just as reported in the main text where we assumed complete inactivation. (**B**) *Limited information model*. Noise correlations in the limited information model, unlike the extensive information model, were not known *a priori*, but were instead fit to explicitly account for the behavioural effects of inactivation. Consequently, both the readout weights and the inactivation fractions are jointly constrained by the behavioural thresholds observed after inactivating these brain areas. Thus the set of inactivation fractions consistent with data co-varied with readout weights. Shaded regions represent fraction of cortex inactivated for MSTd and VIP that were consistent with observed behavioural thresholds following inactivation (within 95% confidence intervals) assuming three different values of the scaling of MSTd readout weights (*a*_*M*_ = 0.95, 0.85, and 0.75, shown in red, green, and blue). The solution space that was consistent with our data (shaded areas) contracted as the scaling of MSTd weights decreased, with no solutions for *a*_*M*_ < 0.74. In contrast to the extensive information model, the limited information model attributes experimental results to overweighting MSTd compared to optimal decoding in all cases (which would have *a*_*M*_ within the intervals [0.87 0.93], [0.75 0.81], and [0.6 0.64] respectively, again to remain consistent with 95% confidence intervals of behavioural thresholds), just as we reported in the main text assuming complete inactivation. Thus the qualitative behaviour of the limited information model was robust to incomplete inactivation by Muscimol.
(PDF)

S16 Fig Recurrent neural network. We extended our model to incorporate recurrent connections and derived theoretical results relating the connectivity matrix to the behavioural and neuronal effects of inactivation in steady-state (**S8.1 Text**). Recall that decoding weights were inferred in the subspace of the leading eigenmodes of the response covariance. Therefore, it is clear that our main results will not be affected by recurrent weights that do not significantly alter neural response along the principal components of covariance in MSTd (*M*) and VIP (*V*). Instead, we constructed a specific recurrent scheme that would couple responses along the leading modes (**S8.2 Text**), and used our theoretical results to test whether there exist connection strengths (*c*) that leave our main conclusions unaltered. **(A)** Schematic of a recurrent neural network comprising the two brain areas – MSTd (*M*) and VIP (*V*). **(B)** Recurrent connectivity matrices for the extensive (EI) and limited information (LI) models. **(C)** Unlike the purely feedforward model, slopes of the tuning curves of individual neurons in this recurrent network are altered when one of the two brain areas is inactivated. **(D)** Ratio of thresholds after inactivating one of the areas to the behavioural threshold observed in the intact brain, as a function of the overall connection strength (*c*) between the areas. For appropriate choice of connection strengths (dotted line), the behavioural effects of inactivation are consistent with the experimentally observed outcomes, and nearly identical to the feedforward network for both limited and extensive information models.
(PDF)

S1 Table Model parameters and predictions for visual condition. Model parameters and predicted changes in CCs following inactivation for the two covariance models, shown as median ± central quartile range. (^**†**^Values correspond to when decoder is inferred using a rank-two approximation of the covariance.). See **Table 1**in main text for vestibular condition.
(PDF)

S1 Text Choice probability and choice correlation. (PDF)

S2 Text Optimal thresholds and coarse-grained covariance. (PDF)

S3 Text Effects of suboptimal decoding on behavioural threshold. (PDF)

S4 Text Effect of suboptimal decoding on choice correlations. (PDF)

S5 Text Combining choice correlations and inactivation effects. (PDF)

S6 Text Effect of measurement uncertainty. (PDF)

S7 Text Modeling partial inactivation. (PDF)

S8 Text Recurrent network model. (PDF)

S9 Text Effect of selective inactivation on choice correlations in the non-inactivated area. (PDF)
