# Supplementary figures and images for: Inferring decoding strategies for multiple correlated neural populations
Source: PLoS Comput Biol. 2018 Sep 24;14(9):e1006371. doi: 10.1371/journal.pcbi.1006371 (PMC6188888; doi:10.1371/journal.pcbi.1006371)

**A**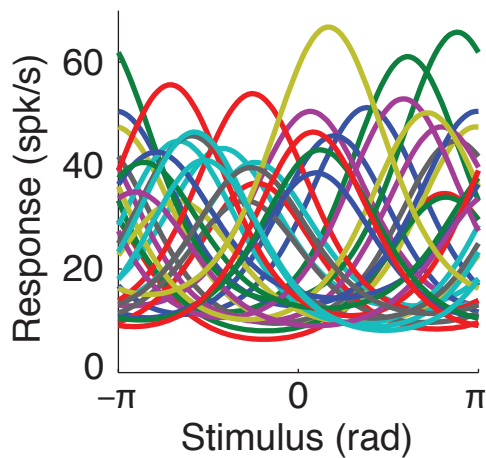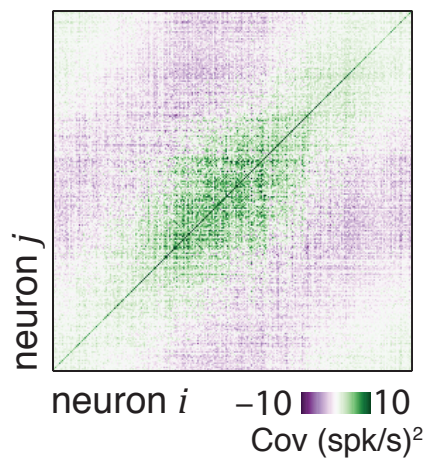**B**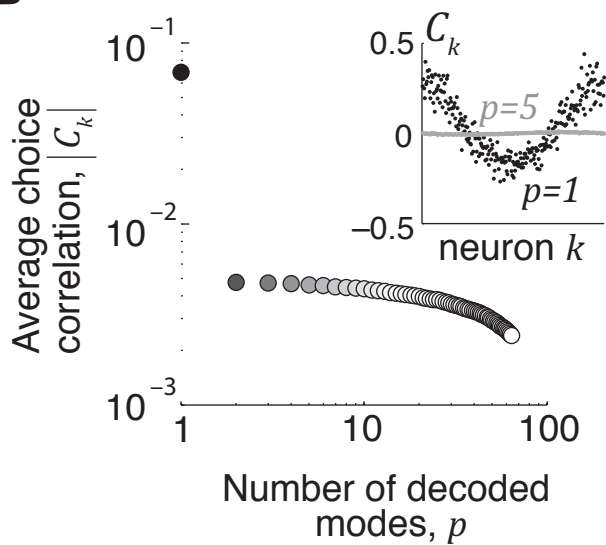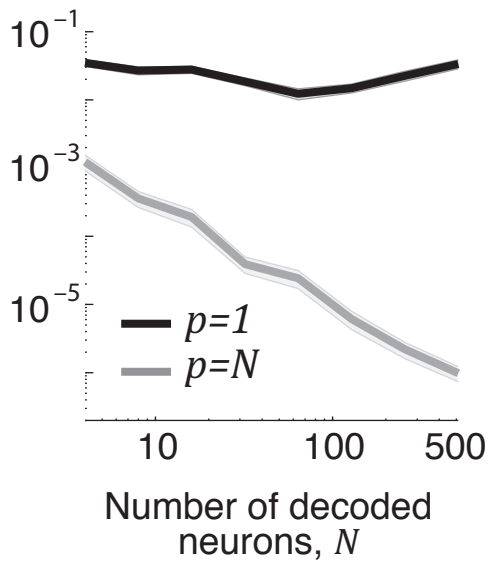

Supplement: S1 Fig — (A) Tuning functions fi(s) (left) and covariance matrix Σ (right) of a subset of model neurons used in this simulation. The stimulus s ∈ (−π,+π] was a circular variable and tuning followed a von Mises function: fi(s)=bi+hieκicos(s−si) where baseline and height bi and hi were drawn from Poisson distributions bi∼Poiss(b¯) and hi∼Poiss(h¯) with means b¯ = 5 spikes/sec and h¯ = 15 spikes/sec, tuning peakiness κi was sampled from the rectified normal distribution κi∼|N(1,0.25)|, and preferred stimulus si was drawn from a uniform distribution. Covariance Σij between neurons i and j was Σij=Rijfifj where noise correlation coefficient Rij was proportional to signal correlation (Eq (8)) with a proportionality of 0.2. (B) Neurons were linearly decoded by confining readout weights to the leading p eigenmodes of the covariance. Weights were always chosen to be optimal within the decoded subspace, and p was varied from 1 to N where N = 512 denotes the population size. The root-mean-squared choice correlation CRMS over all neurons decreases with p: for this model population, it drops by an order of magnitude already for p = 2. Inset shows Ck of each neuron for two example cases. (C) Choice correlations tend to decrease with population size when all modes are decoded optimally (gray: p = N), but remain insensitive to population size when only the leading mode is decoded (black: p = 1). (PDF) [file pcbi.1006371.s001.pdf]

decoder scaling

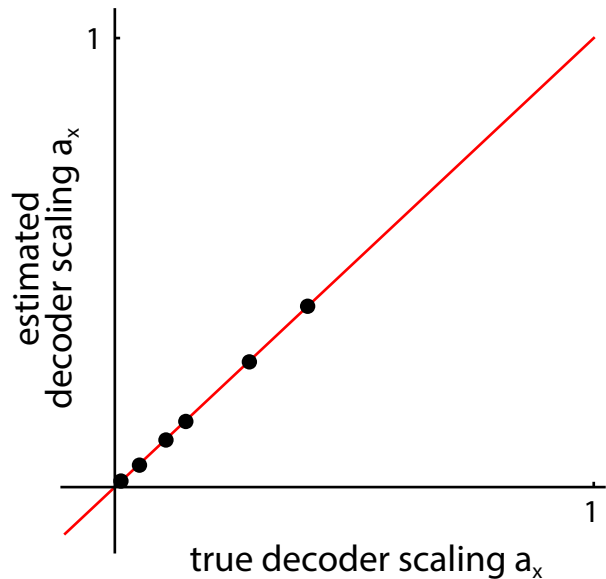

population-level noise

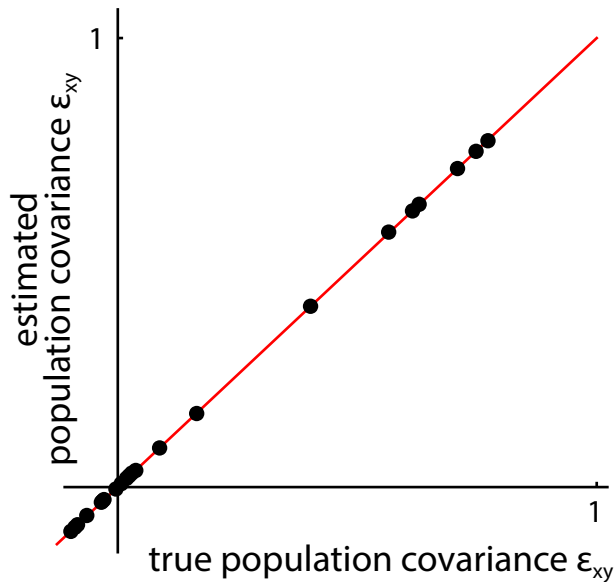

Supplement: S2 Fig — In this demonstration, 6 populations with information-limiting noise are each manipulated by a random multiplicative inactivation factor. We successfully recover decoder scalings (left) and population noise covariance (right) using behavioural thresholds and choice correlation slopes during these inactivation experiments by numerically solving Eqs (18) and (19). (PDF) [file pcbi.1006371.s002.pdf]

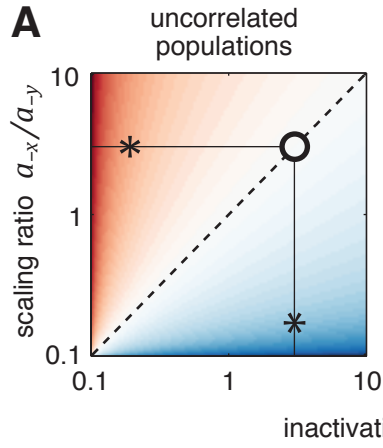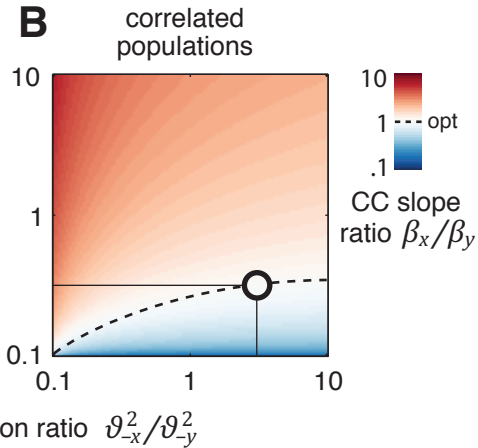

Supplement: S3 Fig — Consider two populations x and y with relative scaling of neuronal weights ax and ay. These scalings depend not only on the post-inactivation thresholds (ϑ−x and ϑ−y) but also on the magnitude of their choice correlations (βx and βy) according to Eqs (20) and (21). The two panels illustrate the relative choice correlation magnitudes (βx/βy, color) for uncorrelated populations (Eq (20)) and correlated populations (Eq (21)), as a function of the scaling ratio ax/ay and the inactivation ratio (ϑ−x2/ϑ−y2). For simplicity, here we assume that βy = 1, so βx/βy = 1 corresponds to optimal decoding. (A) For systems in which the two populations are uncorrelated (εxy = 0), the scaling ratio ax/ay is directly proportional to inactivation ratio ϑ−x2/ϑ−y2. Nonetheless the slope of this relationship depends on the ratio of choice correlation magnitudes βx/βy (isochromatic contours), so a population with a larger weight could produce a smaller deficit upon inactivation, or vice-versa (black asterisks). Inactivation effects exactly match the ratio of scalings (e.g. black open circle on the main diagonal) only if decoding is optimal (black dashed line). (B) When the populations are correlated, the scaling ratio is no longer proportional to the inactivation ratio. Instead, their relationship is nonlinear (black dashed line), and the two ratios may not match even if decoding happens to be optimal (e.g black open circle). In other words, the change in behavioural threshold does not match how much each area is decoded. Here cross-population correlation εxy is εxxεyy/2 for illustration. (PDF) [file pcbi.1006371.s003.pdf]

**A**

Vestibular

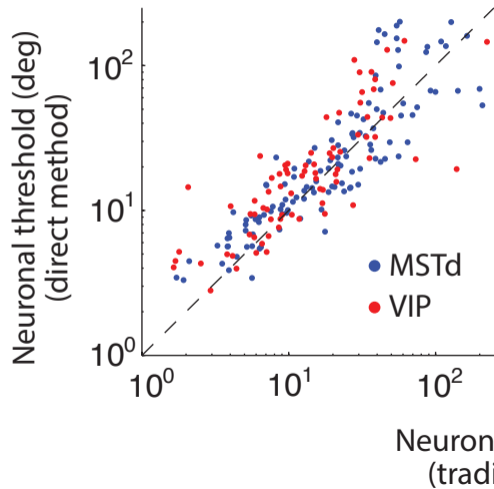**B**

Visual

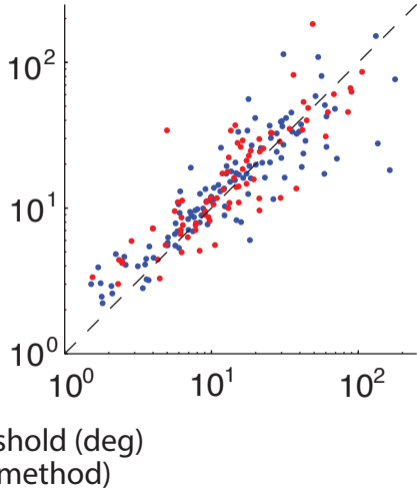

Supplement: S4 Fig — Each neuron’s threshold was estimated in two ways—directly as the inverse square-root of its Fisher information at s = 0 (Methods – Eq (23)), or using a traditional approach by constructing a neurometric function. The latter approach used ROC analysis to compute the ability of an ideal observer to discriminate between two oppositely-directed headings (e.g., –6.4° vs. +6.4°) based solely on the firing rate of the recorded neuron and a presumed 'antineuron' with opposite tuning[1]. ROC values were plotted as a function of heading, resulting in neurometric functions that were fit with a cumulative Gaussian function. Neuronal threshold was then defined as the standard deviation of the fitted Gaussian, but increased by a factor of 2 to adjust for the extra information from the antineuron. This 2 adjustment arises because a decision based on a neuron-antineuron pair has twice the signal amplitude but also twice the noise variance, compared to a single neuron and a fixed, noiseless 0° reference. Note that this factor of 2 differs from past studies[2] that assumed a noisy 0° reference heading and thus corrected by a factor of 2. (A) The two methods yielded very similar estimates for vestibular thresholds across neurons in both MSTd (blue, Pearson’s correlation r = 0.55, p = 4 × 10^−11^) and VIP (red, r = 0.31, p = 5 × 10^−3^). (B) Similar results were found for visual thresholds: MSTd (blue, r = 0.65, p = 3 × 10^−9^) and VIP (red, r = 0.87, p = 1 × 10^−20^). For these comparisons, we omitted a small subset of insensitive neurons (Vestibular: 4/129 MSTd neurons and 7/88 VIP neurons, Visual: 1/129 MSTd neurons and 5/88 VIP neurons) with extremely large thresholds (>300°). (PDF) [file pcbi.1006371.s004.pdf]

Vestibular

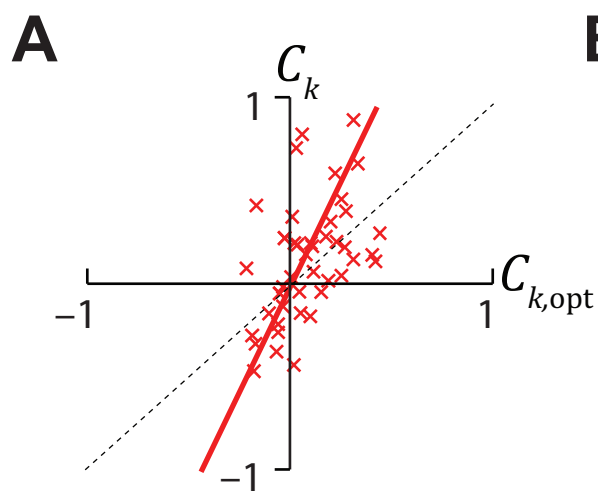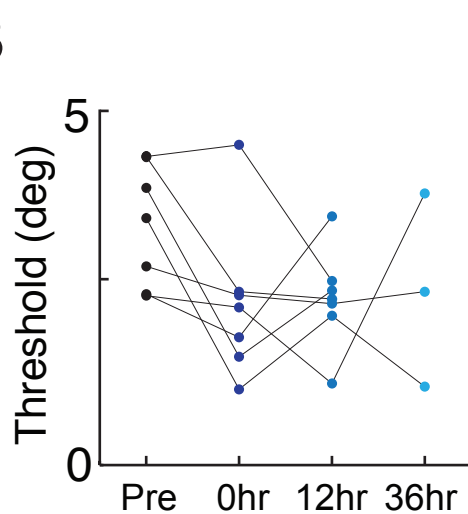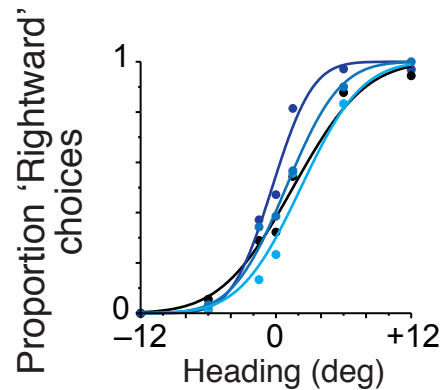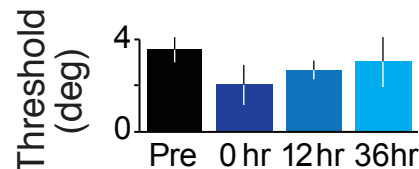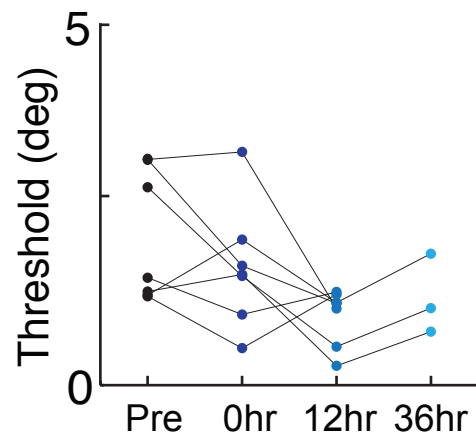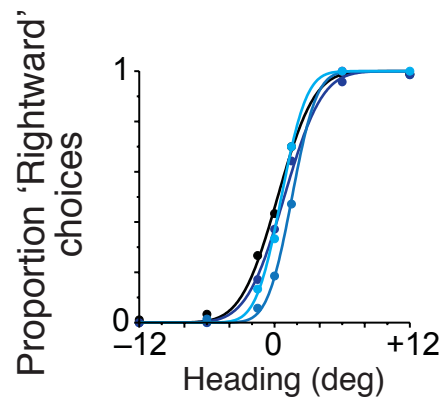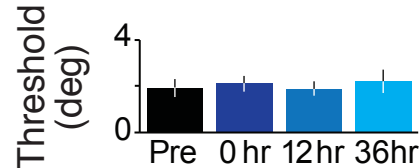

Visual

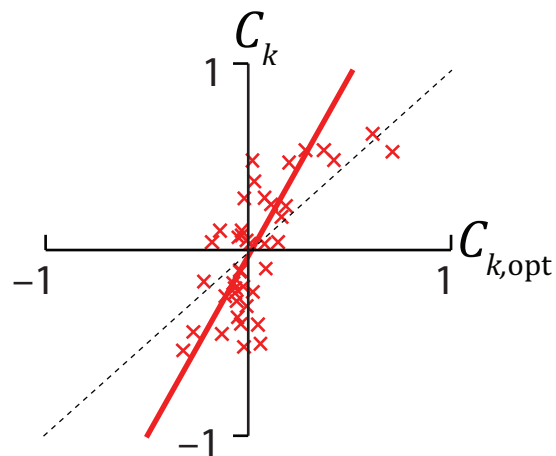

Supplement: S5 Fig — (A) Choice correlations of VIP neurons. Neural recordings were carried out in a separate monkey X prior to inactivation of area VIP, while he performed a heading discrimination task whose structure was identical to that described in Methods in all regards, except each trial lasted only 1s instead of 2s. Similar to those in monkeys C and U, neuronal choice correlations in area VIP are proportional to but greater than those expected from optimal decoding of these neurons during both vestibular (top) and visual (bottom) heading discrimination tasks. The 95% CI of slopes βV were found to be [1.9 2.9] and [1.2 1.8] for the vestibular and visual conditions respectively. (B) Behavioural effects of VIP inactivation. Left: Discrimination thresholds at different times (different shades of blue) following inactivation of VIP, for all seven experiments conducted on monkey X. Thresholds obtained in a single experimental session are connected by a line. Across experiments, inactivating area VIP failed to elicit significant changes in either the vestibular or visual conditions. The behaviour of this monkey was tested 36 hours following inactivation in only 3 of the 7 experiments. Right: Psychometric functions at different times during inactivation of area VIP, averaged across experiments, for the vestibular (top) and visual (bottom) conditions. Behavioural thresholds computed from the psychometric functions at different times are shown in the bottom panels. None of the comparisons were significant (Wilcoxon rank-sum test, significance-level of p = 0.05). Error bars indicate standard error of the mean. (PDF) [file pcbi.1006371.s005.pdf]

Monkey A

Monkey C

Monkey U

Monkey X

Vestibular

VIP

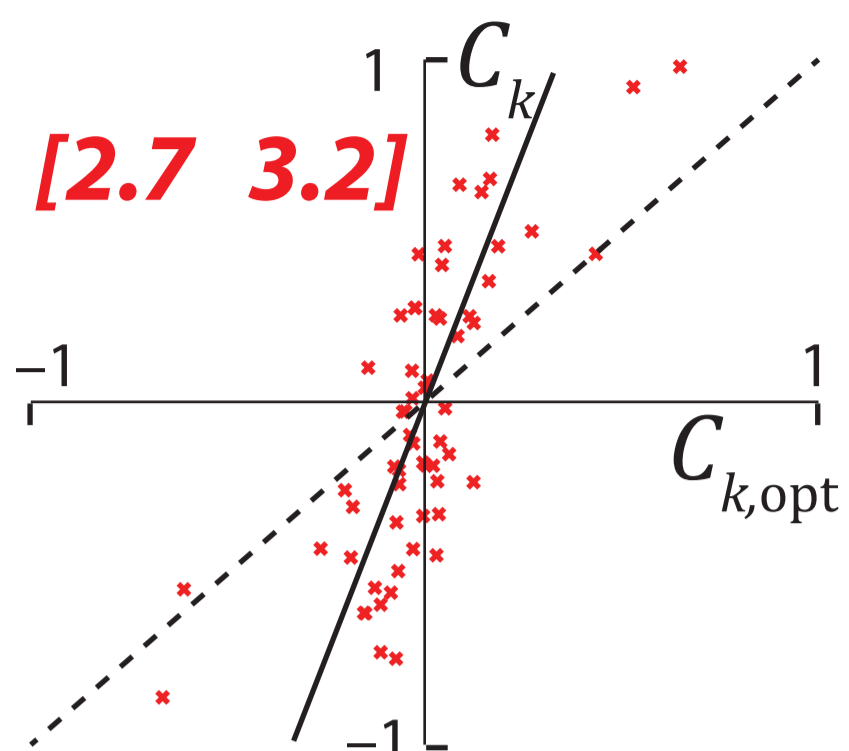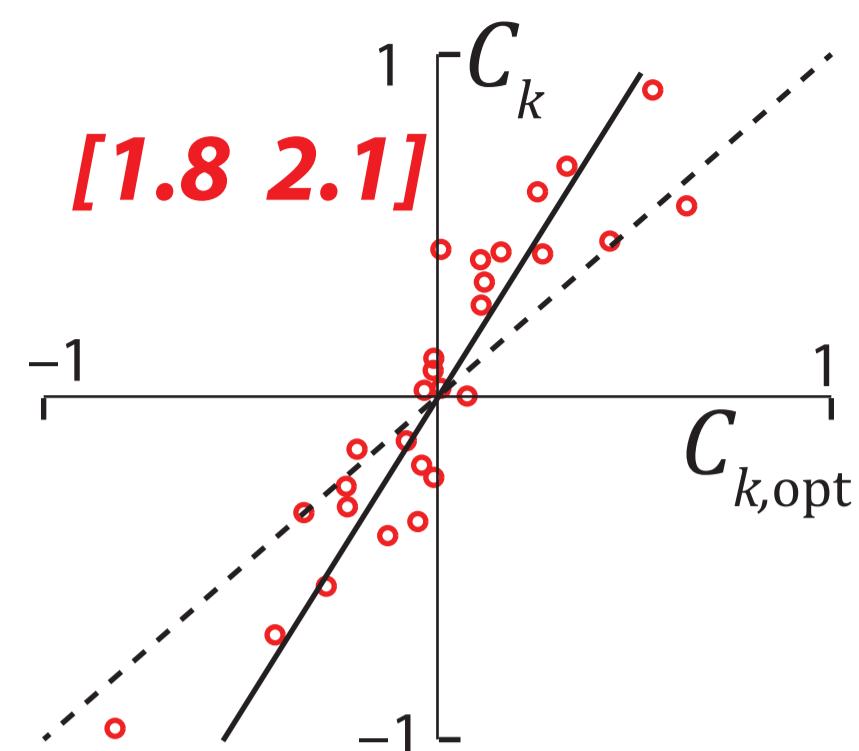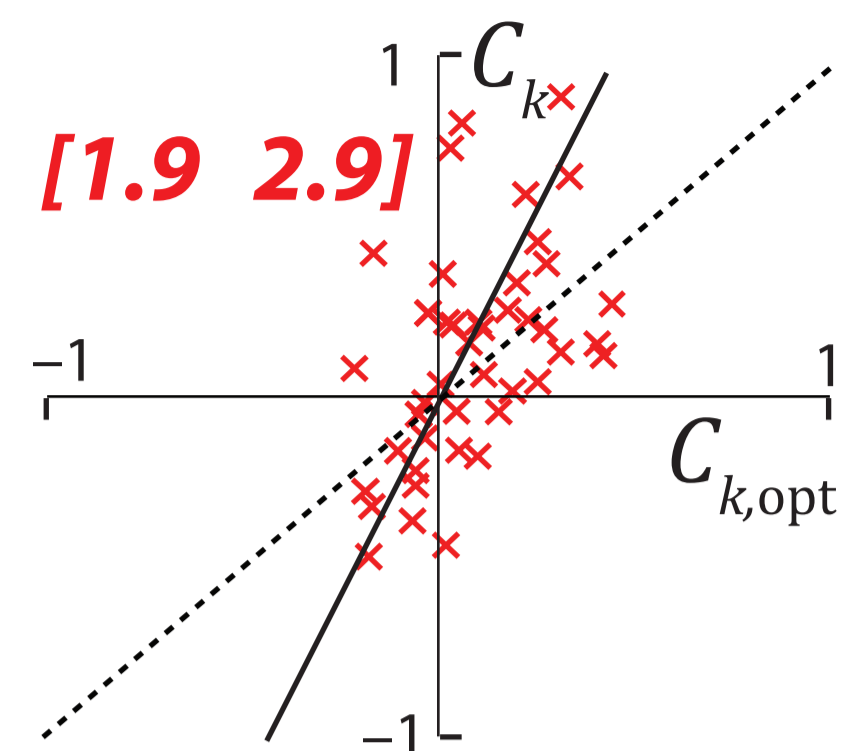

MSTd

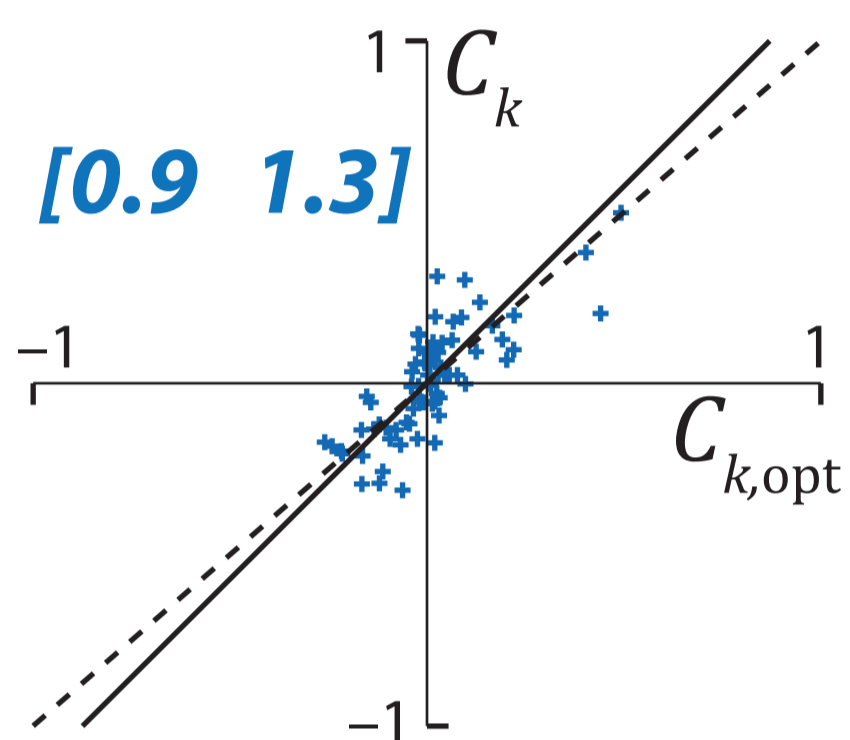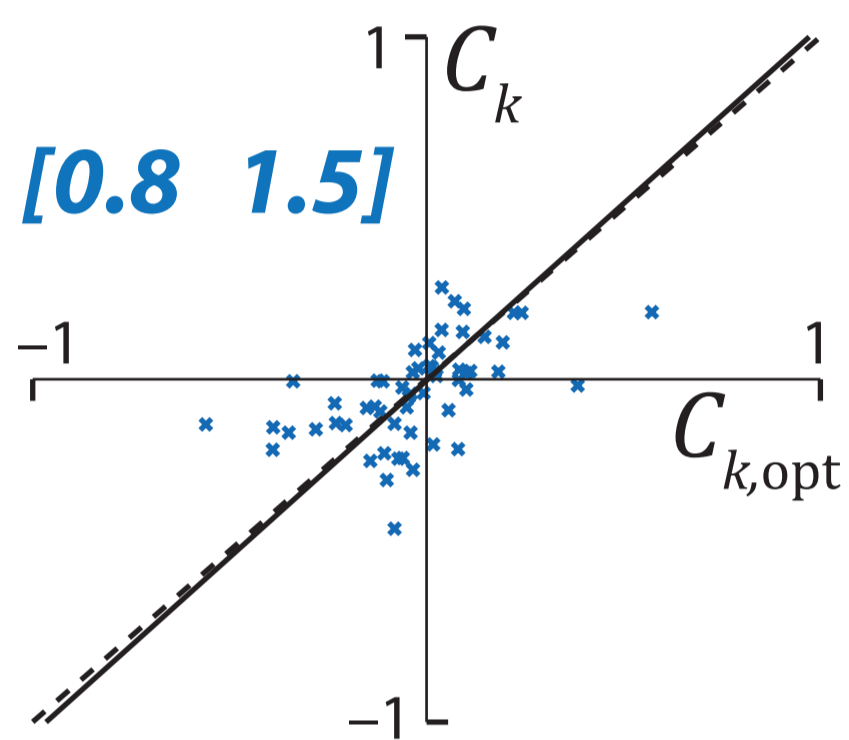

Visual

VIP

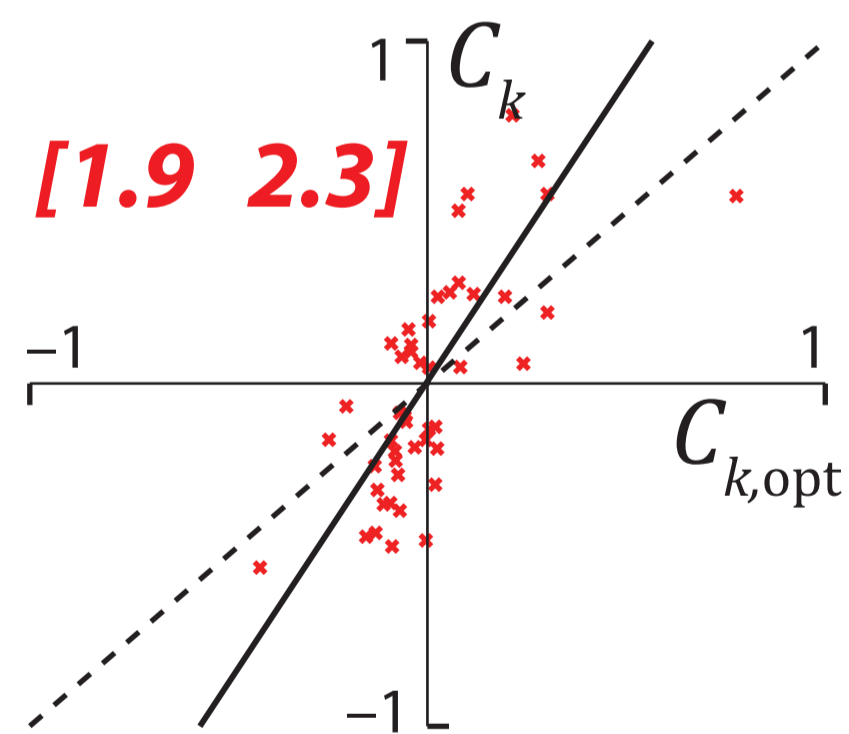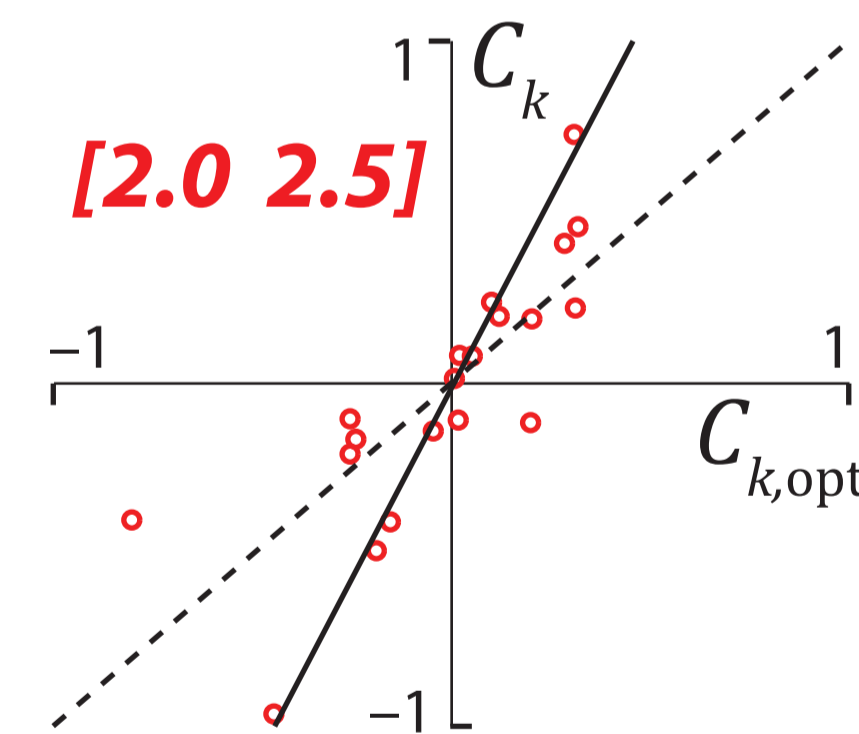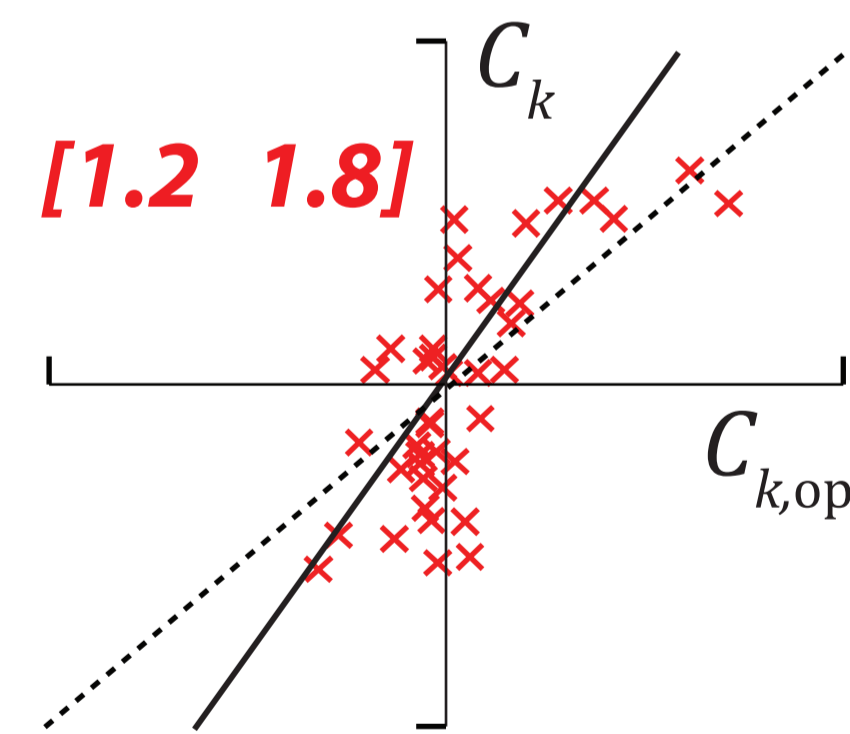

MSTd

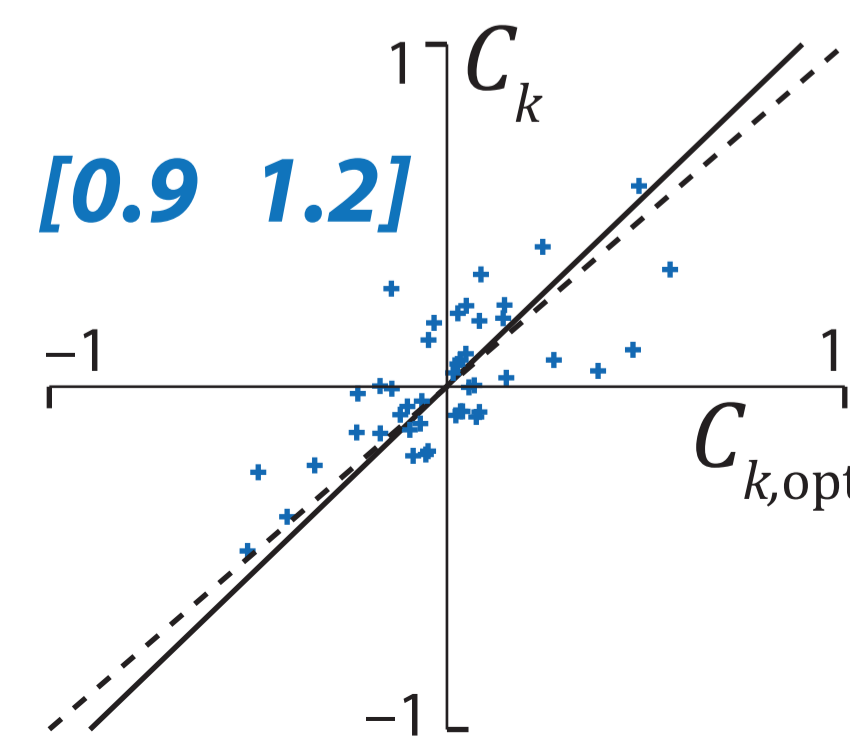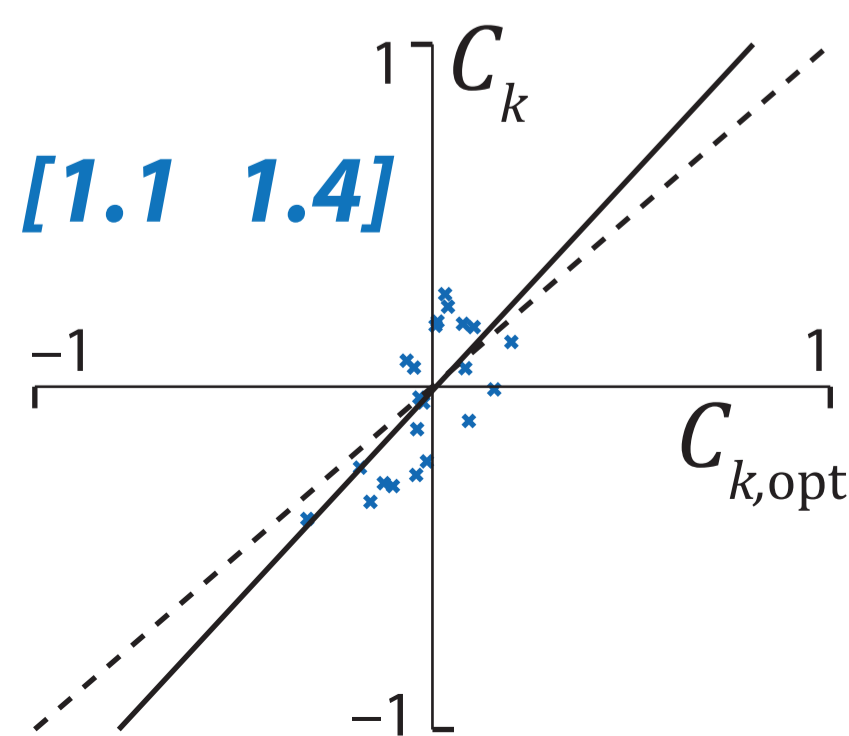

Supplement: S6 Fig — Experimentally measured choice correlations (Ck) of neurons in MSTd (blue) for both the vestibular (top) and the visual (bottom) condition are close to optimal predictions (Ck,opt), those of VIP neurons are systematically greater (red). This observation holds individually in each monkey. Solid black lines correspond to the best linear fit. Vestibular data in monkeys C and U are replotted from Ref.[15] with different sign convention (see Methods). Monkey A was used only for MSTd recordings, and monkeys U and X only for VIP. (PDF) [file pcbi.1006371.s006.pdf]

Vestibular

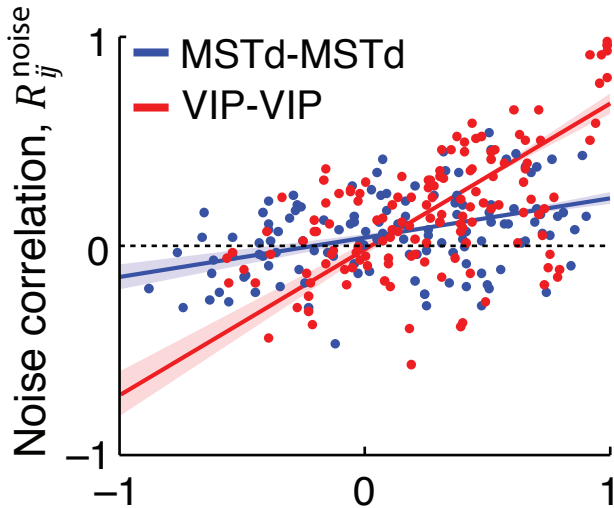

Visual

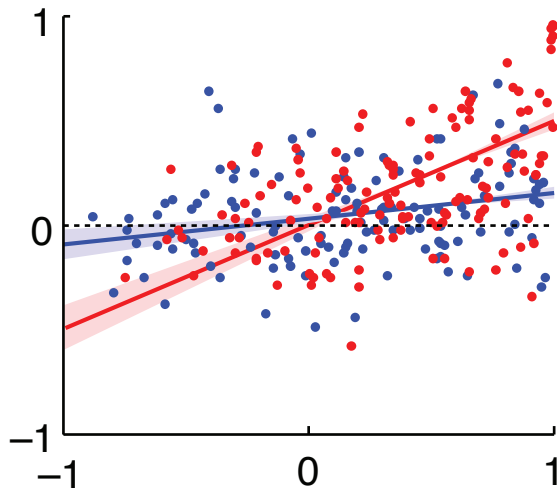Signal correlation,  $R_{ij}^{\text{sig}}$

Supplement: S7 Fig — Pairs of neurons within MSTd (blue; n=127 pairs) and VIP (red; n=139 pairs) were recorded when the animal experienced self-motion in various directions based on either vestibular (left) or visual (right) cues. For each pair of neurons i and j, correlated variability in the firing rates across trials (noise correlation Rijnoise) is plotted against correlated variability in the average firing rates across stimuli (signal correlation Rijsig). The relationship between signal and noise correlation was fit to a linear model (Eq (8)) separately for each area, represented here using straight lines. Shaded areas correspond to 95% confidence intervals of the resulting fits. (PDF) [file pcbi.1006371.s007.pdf]

**A**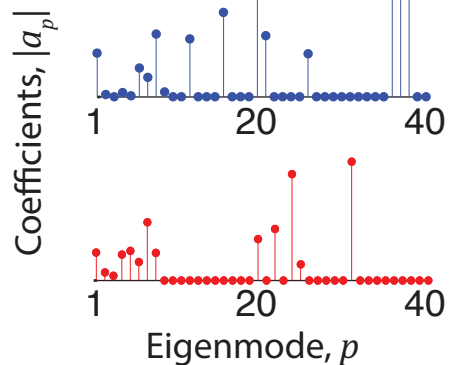**B**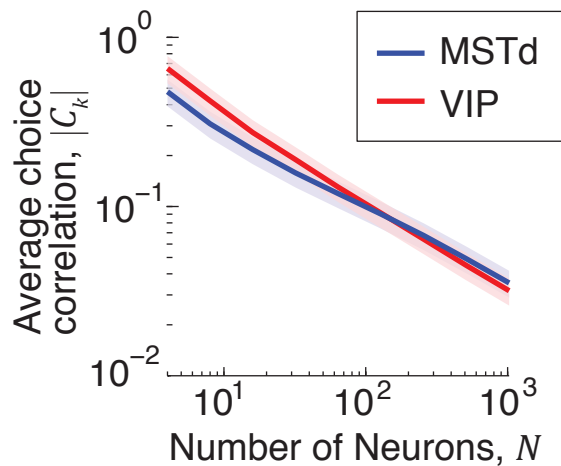**C**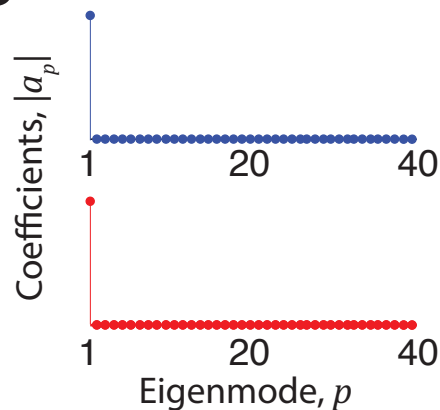**D**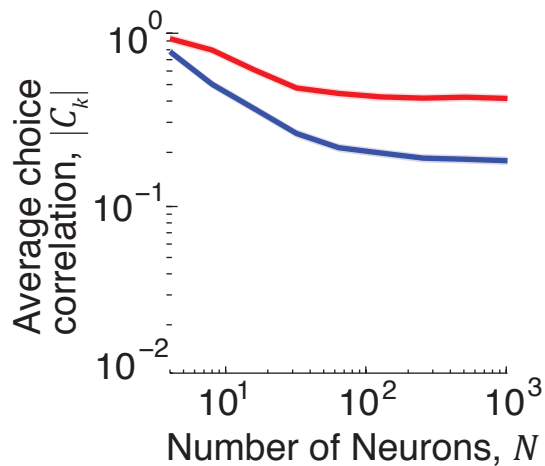**E**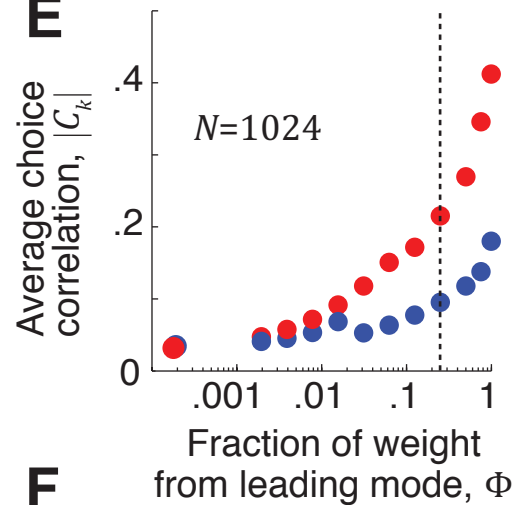**F**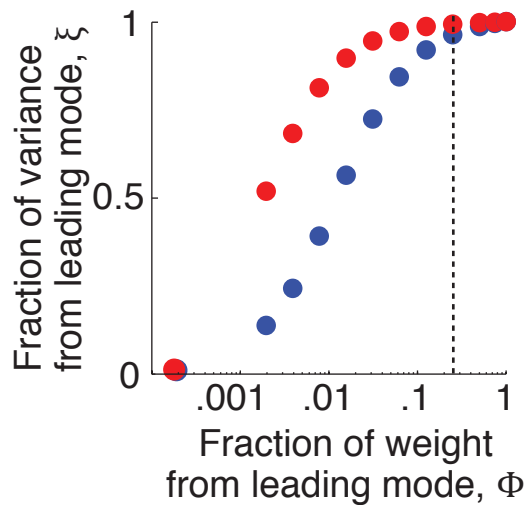

Supplement: S8 Fig — Any readout weight w can be expressed as a linear combination of the eigenvectors up of the response covariance as w ∝ ∑papup where the constant of proportionality is chosen to ensure unbiased decoding. Coefficients magnitudes |ap| indicate how much the different eigenmodes p contribute to behavioural choice. To assess the specific contribution of the leading mode from MSTd and VIP, we considered three different cases: optimal decoding of response along all available modes, a decoder confined to the leading eigenmode in each area, and a spectrum of decoders in between the two extremes. We decoded MSTd & VIP responses separately in all cases using covariance Σ specified by the extensive information model (Fig 4A – left), and examined the average magnitude of choice correlations across all neurons in each case. (A) Optimal decoding of all modes. The pattern of coefficients ap of the optimal decoder of MSTd (blue) and VIP (red) responses. For clarity, only the coefficients corresponding to the leading 40 modes are shown. Evidently, the leading mode has little influence on the decoder output as seen from the magnitude of coefficient a1. (B) The average choice correlation, quantified as the root-mean squared (RMS) choice correlations of the set of all neurons, decreases to ~0.01 even for the modest population size of N = 1000 neurons. (C,D) Decoding leading mode only. Plotted as for A,B, but restricting the readout to one leading eigenmode. We forced the coefficients ap to zero for all p ≠ 1, yielding w ∝ u1. Choice correlations implied by this decoder asymptote to about 0.2 and 0.4 for MSTd and VIP, values that are of the same order of magnitude as seen in the experiments. (E) Varying weight on leading mode. We tested whether the leading mode must contribute substantially to choice, in order to generate high choice correlations. To test this, we first parametrised the contribution of the leading mode as the fraction Φ of weight power it contributes to decoding, accordin [file pcbi.1006371.s008.pdf]

**A**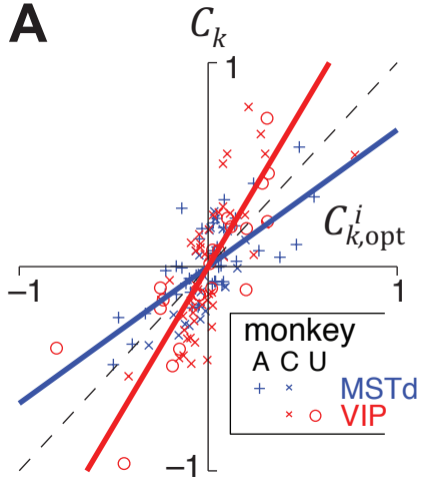**B**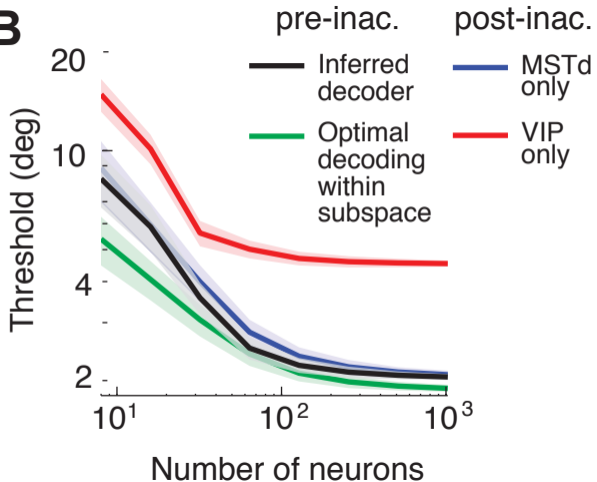

Supplement: S9 Fig — (A) Experimentally measured choice correlations (Ck) of individual neurons in MSTd (blue) and VIP (red) are plotted against the ith component Ck,opti of choice correlations generated from optimally decoding the responses within the subspace of two leading principal components of noise covariance. When two populations are not correlated with each other, the two leading components of the global noise covariance correspond to the largest noise modes in each population separately. Consequently Ck,opt1 and Ck,opt2 correspond to optimal choice correlations in VIP and MSTd, respectively. (B) Performance (threshold) of a decoder with weights inferred from the subspace of two leading principal components of the noise covariance. The black and green lines indicate the performance of the inferred and optimal decoders within this subspace. Inactivating VIP is correctly predicted to have no effect on behavioural performance (blue), while MSTd inactivation increases the threshold (red). Shaded region indicates ±1 SEM. (PDF) [file pcbi.1006371.s009.pdf]

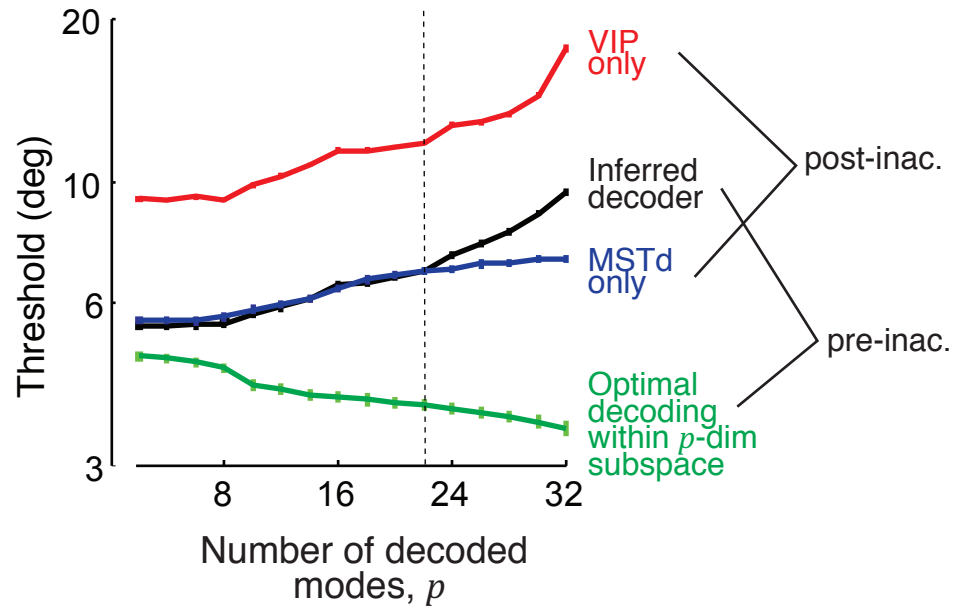

Supplement: S10 Fig — Since decoding performance was nearly saturated at 256 neurons (Fig 5C), we fixed the size of the neural population at N = 256, and examined the behavioural threshold when varying the dimensionality of the decoded subspace. Decoding weights were inferred in the subspace spanned by a total of p eigenvectors of the covariance matrix, using p/2 eigenvectors in both MSTd and VIP. The decoder continued to correctly predict the qualitative effects of inactivating MSTd and VIP beyond the 2-dimensional subspace considered in Fig 5, roughly until about p = 22 (vertical dashed line). Note that the threshold predicted by the optimal decoder within the restricted subspace (green) improves as more (informative) dimensions are included, while that of the inferred decoder worsens. Therefore, readout weights extract more noise than signal from these additional dimensions. This makes sense because if it the weights were instead tuned to decrease the variance in the estimate as more dimensions are added, they would no longer explain the large measured choice correlations. One reason why the experimental predictions of this model break down for large p is that the predictions are only reliable in the regime of small p where the effect of measurement noise is low. This is because the reliability of inferred decoding weights (and consequently also its predictions) is inversely related to the eigenvalue of the decoded mode, so reliability of the predictions worsens as p increases (S6 Text). (PDF) [file pcbi.1006371.s010.pdf]

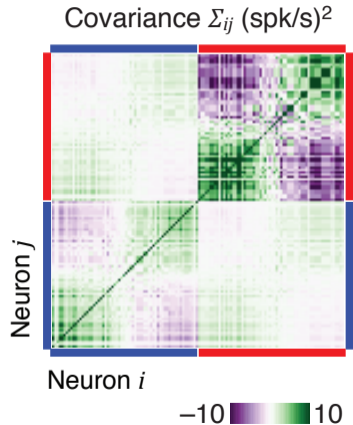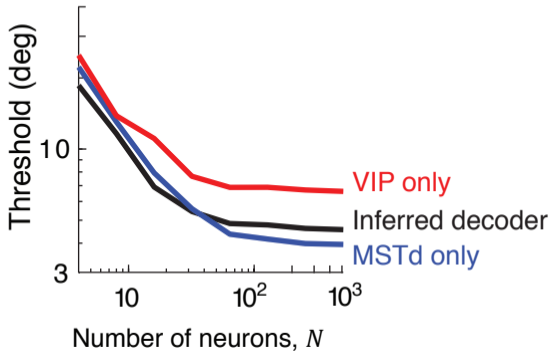

Supplement: S11 Fig — Left: A representative covariance matrix when neurons in MSTd and VIP are mildly correlated through the leading noise modes (εxy≈0.2εxxεyy). Right: In contrast to the observed effects of inactivation, the decoder inferred using the covariance on the left incorrectly predicted that inactivating VIP should reduce the behavioural threshold. This was unlike the decoder shown in Fig 5C that correctly predicted the effects of VIP inactivation when correlations between the two areas were zero on average. (PDF) [file pcbi.1006371.s011.pdf]

**A**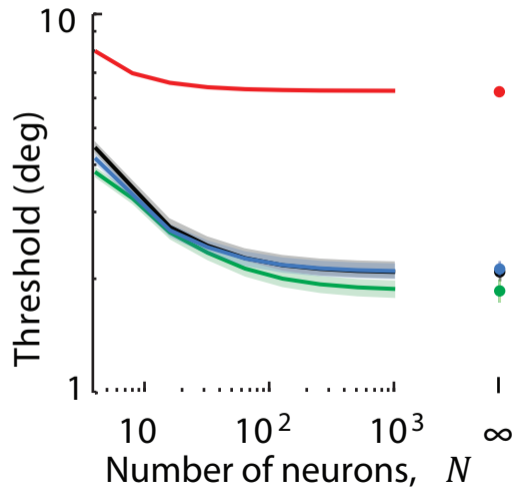**B**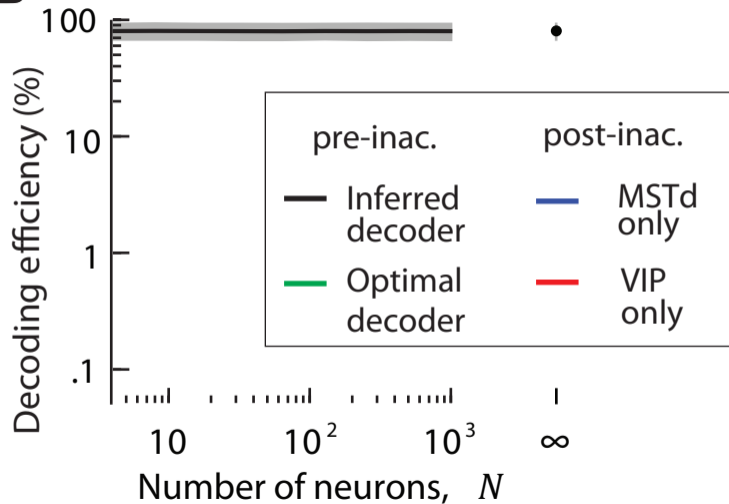

Supplement: S12 Fig — (A) Like decoding in the presence of extensive information, this decoder is suboptimal (black vs green), and can account for the behavioural effects of inactivation. (B) Unlike decoding in the extensive information model, the efficiency of this decoder is quite high and insensitive to population size. Shaded areas represent ±1 SEM. (PDF) [file pcbi.1006371.s012.pdf]

**A**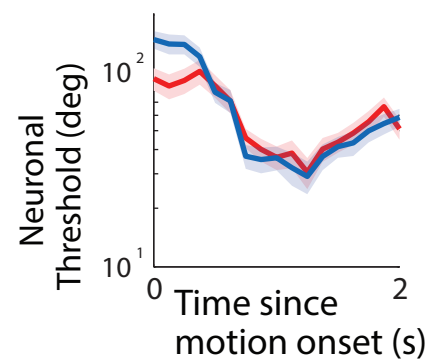**C**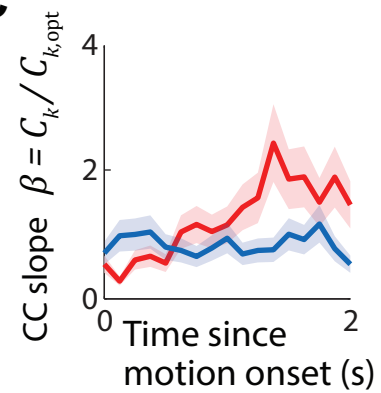**E**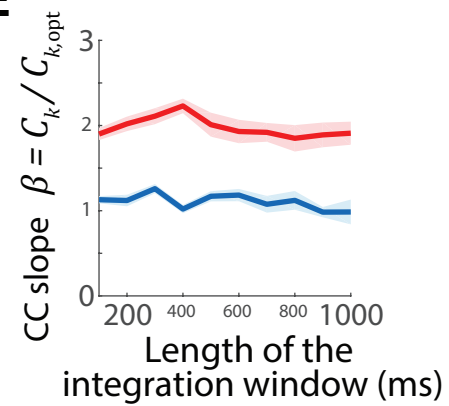**B**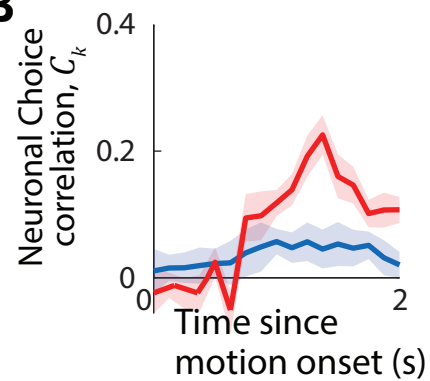**D**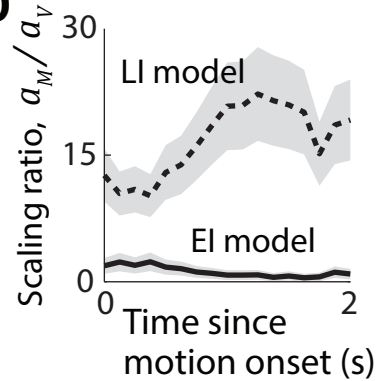**F**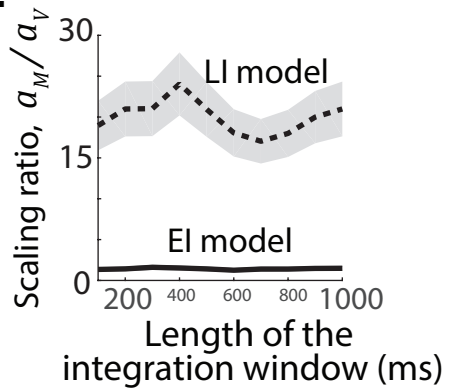

Supplement: S13 Fig — Neuronal thresholds (A) and choice correlations (B) were computed for each neuron across the duration of the trial using a 250ms moving window and averaged across neurons. Note that these readouts predict the choice based only on a single time window per data point, and do not perform a weighted sum of responses in multiple windows. Neuronal thresholds in both brain areas were comparable at all times, yet the choice correlations (CCs) differed between brain areas VIP and MSTd in a consistent manner over time. Although CCs in both areas peaked around the middle of the trial, those in VIP were proportionally larger at almost all times. (C) Consequently the slopes, β = Ck/Ck,opt, that related observed and optimal choice correlations were generally greater in area VIP than in MSTd. (D) The readout weights inferred using the two models remain largely constant throughout the trial, and are qualitatively consistent with the conclusions drawn from our analyses presented in the main text: the extensive information model implies that area MSTd is underweighted, whereas the limited information model predicts the opposite. Symbols aM and aV denote scaling of readout weights of areas MSTd and VIP respectively. (E) Regression slopes are minimally affected by the length of the analysis window. Both observed neuronal choice correlations as well as those implied by optimal decoding of MSTd and VIP populations increased similarly with the length of the analysis window, leaving the regression slopes β = Ck/Ck,opt largely invariant with the window length for both VIP (red) and MSTd (blue). (F) The qualitative difference in the readout weights inferred using the two noise models are consistent across different lengths of analysis window. Error bars denote ±1 standard deviation. (PDF) [file pcbi.1006371.s013.pdf]

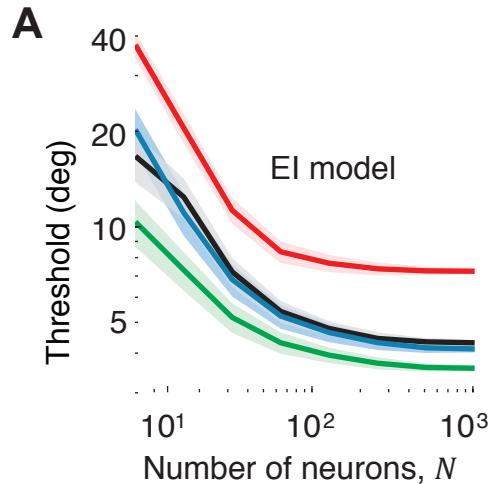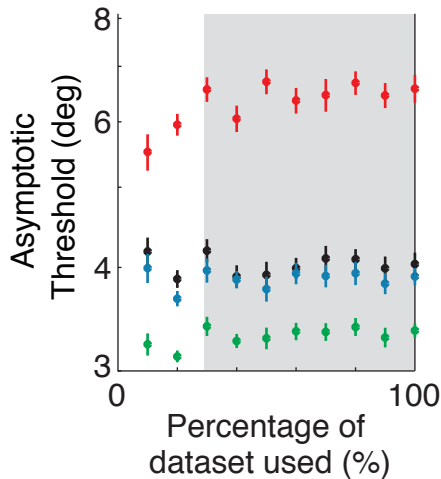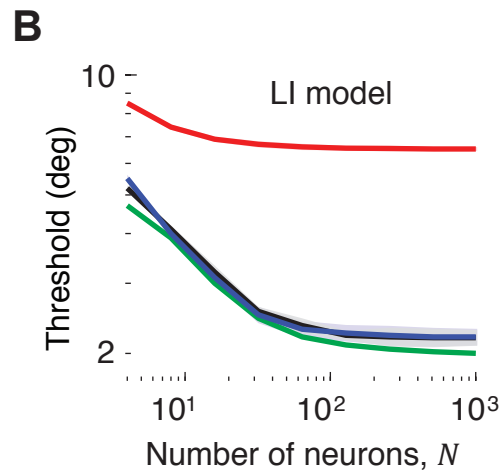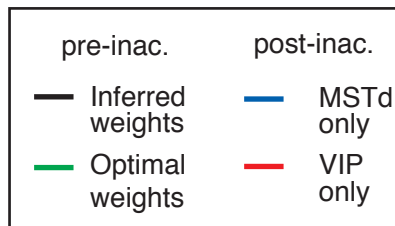

Supplement: S14 Fig — In the main text, we presented thresholds predicted by decoders inferred using the Extensive information (EI) (Fig 5C) and Limited information (LI) (Fig 6B) models. These thresholds were generated by extrapolating a limited dataset containing 129 and 88 neurons from MSTd and VIP respectively. However, those thresholds approached saturation only around 60-70 raising the possibility that those results might be sensitive to the exact number of neurons that were used for extrapolation. To test whether this was the case, we repeated all our analyses by considering only a fraction of the recorded neurons for extrapolation. (A) Left: Thresholds implied by the EI model obtained by extrapolating 50% of the neurons in our dataset (n=65/129 and 44/88 neurons in MSTd and VIP). Thresholds were found to asymptote to nearly the same value obtained by extrapolating the full dataset (compare with Fig 5C). Right: We repeated this procedure for different percentages (10%–100%) and found that our results can be reproduced with as little as 30% of the dataset. The asymptotic thresholds (evaluated at a population size of N = 1024 neurons) do not change much beyond this point (shaded region). (B) Thresholds implied by the LI model obtained by extrapolating 50% of the dataset. Once again, this was similar to results obtained using the full dataset (Fig 6B). (PDF) [file pcbi.1006371.s014.pdf]

**A**

Extensive information (EI) model

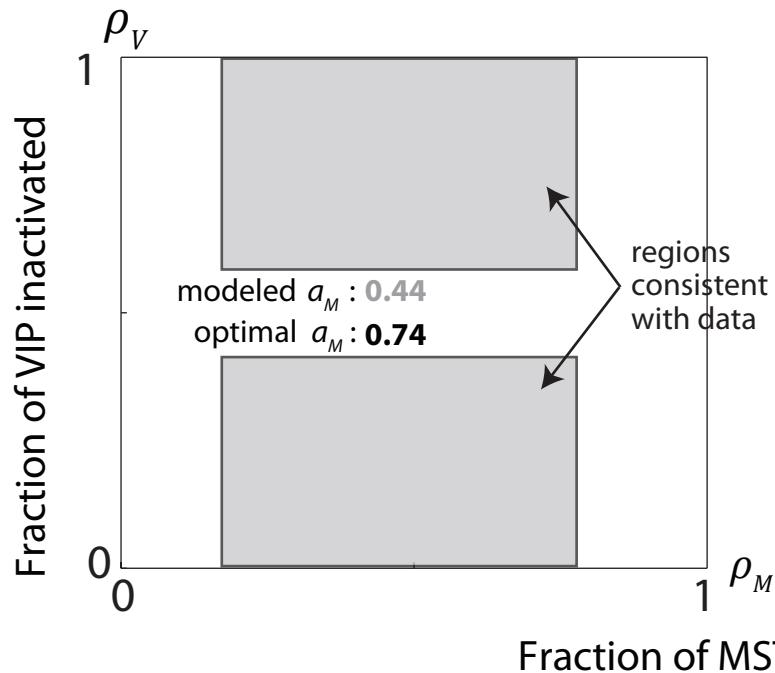**B**

Limited information (LI) model

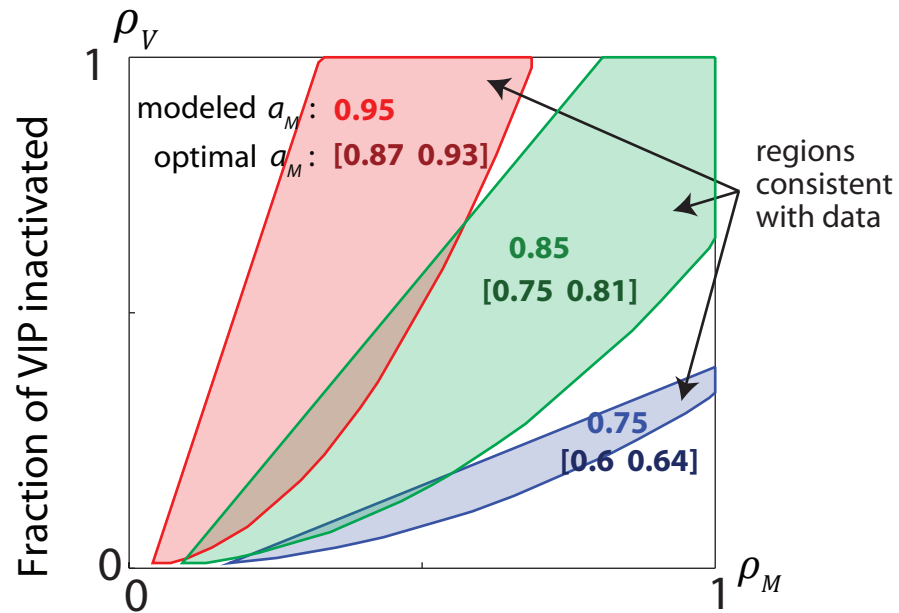

Supplement: S15 Fig — We extended our model to include two additional parameters ρx and ρy that denote fractions of neurons inactivated in populations x and y, and derived theoretical results that account for partial inactivation of the two populations (S7 Text). We used those results to model partial inactivation of the MSTd and VIP in our dataset, and computed parameter ranges in the (ρM,ρV) parameter space (shaded areas) that are consistent with 95% confidence intervals around experimental data. (A) Extensive information model. Since an empirical trend between neural tuning and noise covariance was used to determine the structure of noise correlations, the readout weights could be uniquely determined from the observed pattern of choice correlations (CCs) independent of the extent of inactivation. Therefore the inferred readout weights remained the same as for the model that assumed complete inactivation (inferred MSTd weight scaling aM = 0.44; optimal MSTd weight scaling aM = 0.74). Nonetheless, the predictions for behavioural thresholds following inactivation of MSTd or VIP (shown in Fig 5B) are quantitatively consistent with the experimental observations (Fig 2B) only for a specific range of inactivation fractions (grey region). Specifically, the inferred readout weights predict that the thresholds should increase by a factor of 1.6 if MSTd was fully removed, yet the observed increase was only 1.2±0.1. This suggests that MSTd could neither have been completely inactivated nor remained completely intact, leading to the exclusion of the regions close to the left and right boundaries. For the EI model, therefore, partial inactivation of MSTd was a better match to the behavioural data. Similarly, inactivating about half of VIP is predicted to significantly reduce the threshold (Fig 8C – top panel). Since this was not observed experimentally, the inactivation parameters within the central horizontal band around 0.5 are excluded from the grey region that is consistent with data. Even with [file pcbi.1006371.s015.pdf]

**A**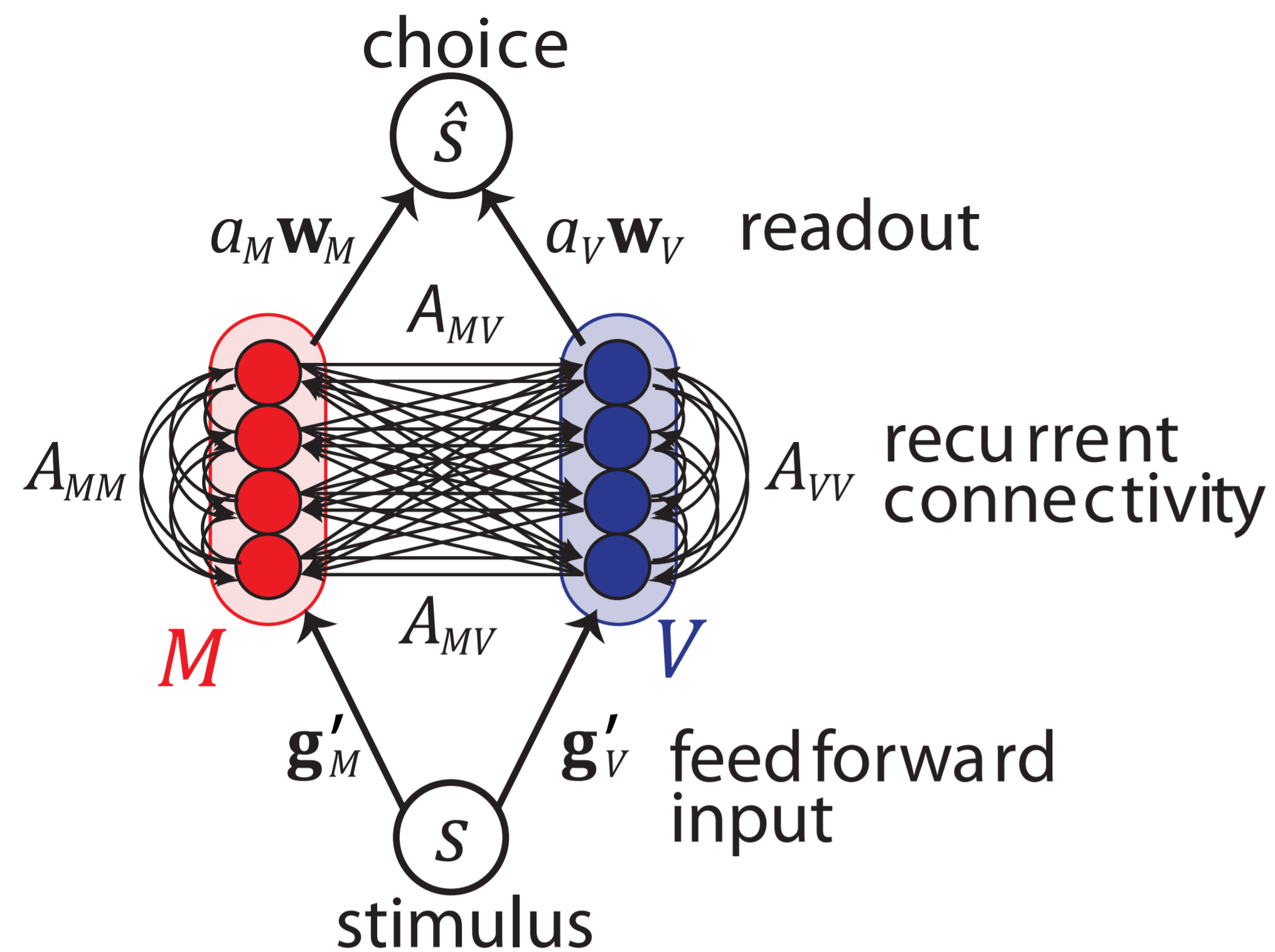**B**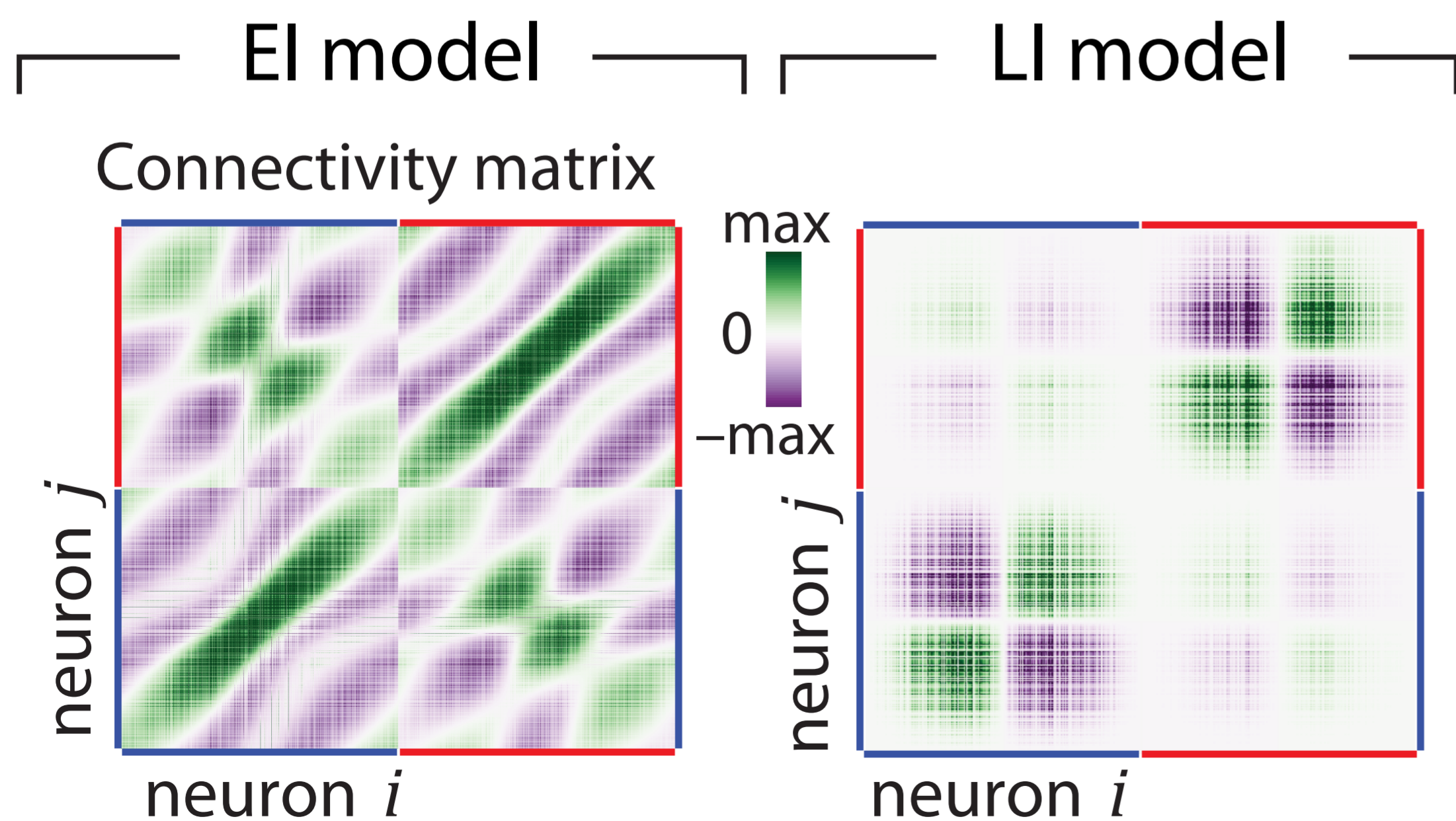**C**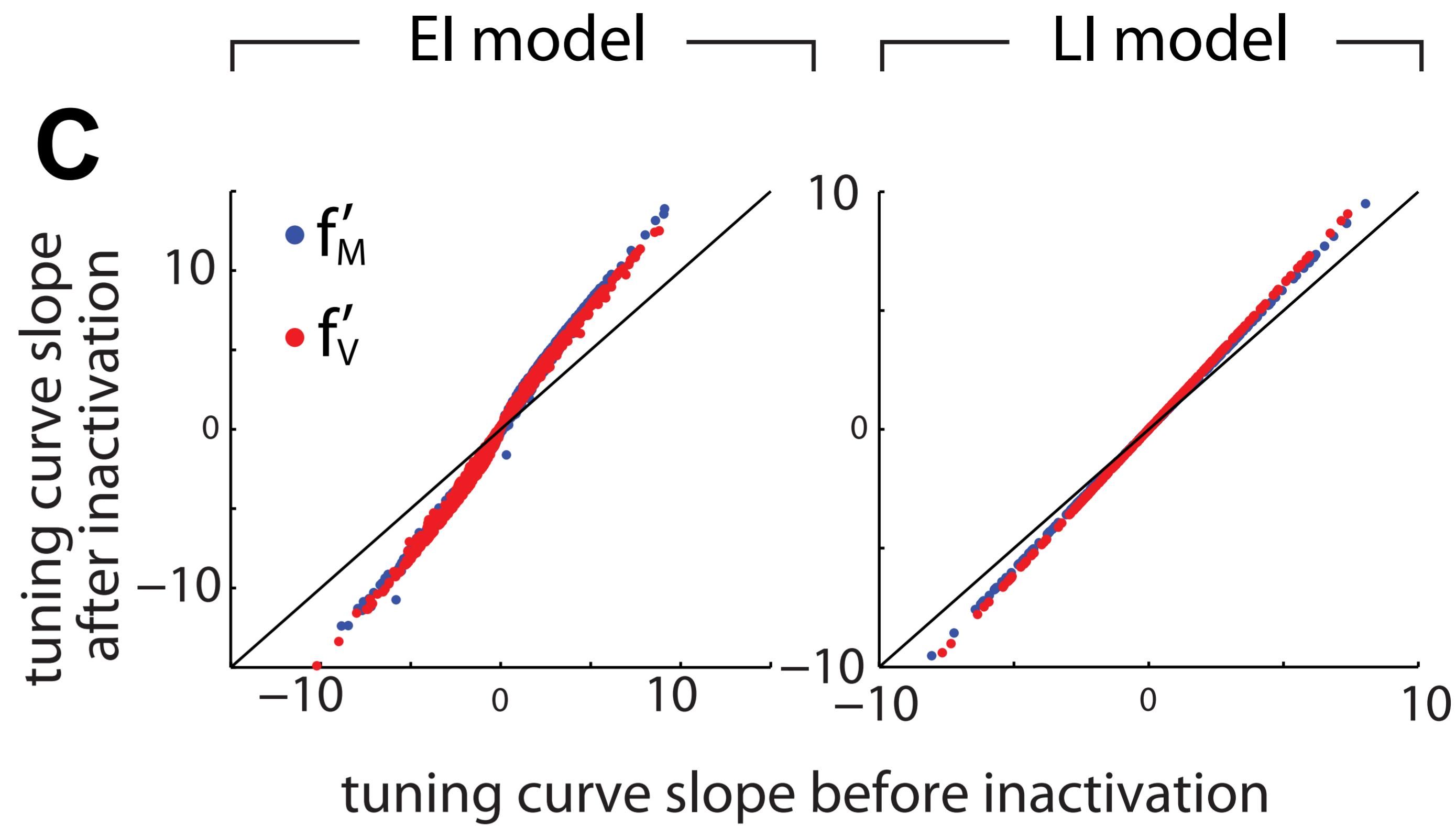**D**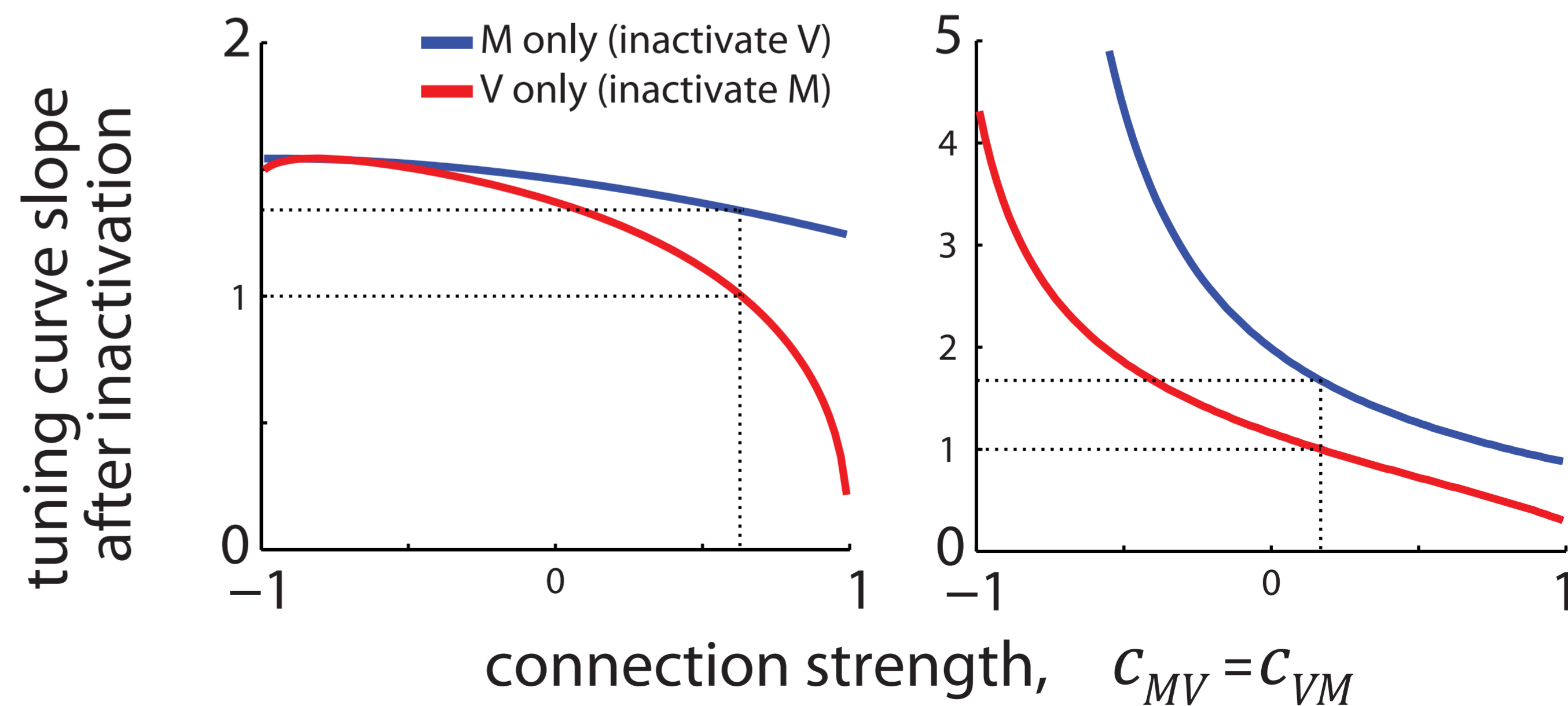

Supplement: S16 Fig — We extended our model to incorporate recurrent connections and derived theoretical results relating the connectivity matrix to the behavioural and neuronal effects of inactivation in steady-state (S8.1 Text). Recall that decoding weights were inferred in the subspace of the leading eigenmodes of the response covariance. Therefore, it is clear that our main results will not be affected by recurrent weights that do not significantly alter neural response along the principal components of covariance in MSTd (M) and VIP (V). Instead, we constructed a specific recurrent scheme that would couple responses along the leading modes (S8.2 Text), and used our theoretical results to test whether there exist connection strengths (c) that leave our main conclusions unaltered. (A) Schematic of a recurrent neural network comprising the two brain areas – MSTd (M) and VIP (V). (B) Recurrent connectivity matrices for the extensive (EI) and limited information (LI) models. (C) Unlike the purely feedforward model, slopes of the tuning curves of individual neurons in this recurrent network are altered when one of the two brain areas is inactivated. (D) Ratio of thresholds after inactivating one of the areas to the behavioural threshold observed in the intact brain, as a function of the overall connection strength (c) between the areas. For appropriate choice of connection strengths (dotted line), the behavioural effects of inactivation are consistent with the experimentally observed outcomes, and nearly identical to the feedforward network for both limited and extensive information models. (PDF) [file pcbi.1006371.s016.pdf]
