# Supplementary material for: Inferring decoding strategies for multiple correlated neural populations
Source: PLoS Comput Biol. 2018 Sep 24;14(9):e1006371. doi: 10.1371/journal.pcbi.1006371 (PMC6188888; doi:10.1371/journal.pcbi.1006371)
Supplement: S1 Table — Model parameters and predicted changes in CCs following inactivation for the two covariance models, shown as median ± central quartile range. (†Values correspond to when decoder is inferred using a rank-two approximation of the covariance.). See Table 1in main text for vestibular condition. (PDF) [file pcbi.1006371.s017.pdf]

**Model**		**Extensive information model**†	**Limited information model**
**Model parameters**	Noise magnitudes	$\varepsilon_{MM} = 6, \varepsilon_{VV} = 25, \varepsilon_{MV} = 0$	$\varepsilon_{MM} = 6, \varepsilon_{VV} = 36, \varepsilon_{MV} = 9$
	Multiplicative scaling of CCs relative to optimal	$\beta_M = 0.67, \beta_V = 1.6$	$\beta_M = 1.2, \beta_V = 2.0$
	Optimal weights	$\lvert a_M/a_V \rvert = 3.8 \pm 0.7$	$\lvert a_M/a_V \rvert = 18 \pm 6$
	Inferred weights	$\lvert a_M/a_V \rvert = 1.5 \pm 0.3$	$\lvert a_M/a_V \rvert = 24 \pm 13$
**Model predictions**	Multiplicative change in CCs following inactivation	$\zeta_M = 1.6 \pm 0.2$<br>$\zeta_V = 1.9 \pm 0.4$	$\zeta_M = 0.8 \pm 0.3$<br>$\zeta_V = 1.4 \pm 0.5$
