# Supplementary material for: Inferring decoding strategies for multiple correlated neural populations
Source: PLoS Comput Biol. 2018 Sep 24;14(9):e1006371. doi: 10.1371/journal.pcbi.1006371 (PMC6188888; doi:10.1371/journal.pcbi.1006371)
Supplement: S1 Text — (PDF) [file pcbi.1006371.s018.pdf]

S1 Choice Probability and Choice Correlation

Neuronal choice probability (CP_k) is, roughly, the probability of correctly guessing the behavioural response on a given trial based only on the response r_k of that particular neuron k. More precisely, it is the probability that a neural response drawn randomly from one choice-conditioned distribution $p(r_k|\text{choice} > 0)$ is greater than another neural response from the same neuron but drawn from the other choice-conditioned distribution $p(r_k|\text{choice} < 0)$. In our model, choice is taken to be the sign of the estimated stimulus $\hat{s}$.

Choice correlation (C_k) is simply the trial-by-trial correlation coefficient between neuronal responses and the animal's internal estimate of the stimulus $\hat{s}$. For a task with only two possible behavioural responses, like heading discrimination, and for Gaussian-distributed neural responses, these quantities are related according to [3]:

$$CP_k = \frac{1}{2} + \frac{2}{\pi}\arctan({2C_k}^{-2} - 1)^{-1/2}$$

The following equation provides an excellent approximation [3] and was used throughout the paper instead of the above equation.

$$CP_k \approx \frac{1}{2} + \frac{\sqrt{2}}{\pi} C_k \qquad \text{(S1.1)}$$

For convenience, we will express all relations in terms of choice correlations. Corresponding expressions for choice probabilities will follow from equations above.
