# Supplementary material for: Inferring decoding strategies for multiple correlated neural populations
Source: PLoS Comput Biol. 2018 Sep 24;14(9):e1006371. doi: 10.1371/journal.pcbi.1006371 (PMC6188888; doi:10.1371/journal.pcbi.1006371)
Supplement: S2 Text — (PDF) [file pcbi.1006371.s019.pdf]

S2 Optimal thresholds and coarse-grained covariance

S2.1 Limited information model: multiple populations

In the limited-information model, the noise covariance is $\Sigma_{\mathrm{IL}} = \Sigma + FEF^T$ **(Eqn (9))**. Elements of E are the covariances of optimal unbiased estimators derived from each population, $E_{xy} = \langle \delta\hat{s}_x \, \delta\hat{s}_y \rangle$. Together, these are related to the variance $\langle \delta\hat{s}^2 \rangle$ of the single global estimate $\hat{s}$ obtained by optimally decoding responses from multiple populations (**Eqn (2)**). The variance of an unbiased, locally optimal linear estimator is equal to the inverse of the linear Fisher information[4], so:

$$\langle \delta\hat{s}^2 \rangle = \left[\mathbf{f}'^{\mathrm{T}} \Sigma_{\mathrm{IL}}^{-1} \mathbf{f}'\right]^{-1} = [\mathbf{1}^{\mathrm{T}} F^{\mathrm{T}} \, \Sigma_{\mathrm{IL}}^{-1} \, F\mathbf{1}]^{-1}$$

where $\mathbf{1}$ is a vector of all ones and we have used the fact that $F\mathbf{1} = \mathbf{f}'$. Applying the Woodbury lemma to express $\Sigma_{\mathrm{IL}}^{-1}$ in terms of Σ^{-1} and E^{-1}, we get:

$$\langle \delta\hat{s}^2 \rangle = [\mathbf{1}^{\mathrm{T}}[(F^{\mathrm{T}} \, \Sigma^{-1} F)^{-1} + E]^{-1} \mathbf{1}]^{-1}$$

The term $(F^{\mathrm{T}} \, \Sigma^{-1} F)^{-1} = \mathrm{Cov}(\hat{\mathbf{s}}|s)$ is the covariance matrix of multiple locally optimal unbiased linear estimators, each from a distinct population, based on an overall noise covariance Σ. If this noise covariance Σ permits extensive information, then $(F^{\mathrm{T}} \, \Sigma^{-1} F)^{-1} \sim O(N^{-1})$ is dominated by the information-limiting covariance E, yielding:

$$\langle \delta\hat{s}^2 \rangle \approx [\mathbf{1}^{\mathrm{T}} E^{-1} \mathbf{1}]^{-1} \tag{S2.1}$$

Similarly, the variance of an estimate $\hat{s}_x$ from optimally decoding just one population, *x*, is:

$$\langle \delta\hat{s}_x^2 \rangle = (\mathbf{f}'^T_x \Sigma_{xx}^{-1} \mathbf{f}'_x)^{-1} + \varepsilon_{xx} \approx \varepsilon_{xx} \tag{S2.2}$$

which is analogous to **Eqn (10)** in the main text.

S2.2 Limited information model: two populations

For the two-population case, **Eqn (S2.2)** becomes

$$\langle \delta\hat{s}^2 \rangle \approx [(1,1)^{\mathrm{T}} E^{-1} (1,1)]^{-1} = \frac{\varepsilon_{xx}\varepsilon_{yy} - \varepsilon_{xy}^2}{\varepsilon_{xx} + \varepsilon_{yy} - 2\varepsilon_{xy}} \tag{S2.3}$$

Equations S2.2 – S2.3 explicitly relate parameters $\varepsilon_{xx}, \varepsilon_{yy}$, and ε_{xy} to the variance of optimal estimates $\hat{s}$, $\hat{s}_x$, and $\hat{s}_y$. Note that these variances are simply the squares of the optimal behavioural thresholds before and after inactivation: $\langle \delta\hat{s}^2 \rangle = \vartheta^2$, $\langle \delta\hat{s}_x^2 \rangle = \vartheta_{-y}^2$ and $\langle \delta\hat{s}_y^2 \rangle = \vartheta_{-x}^2$.

S2.3 Extensive information model

In the coarse-grained extensive information model, just like the case of information-limiting noise, elements $\varepsilon_{xx}, \varepsilon_{yy}$, and ε_{xy} again determine thresholds according to **Eqn S2.2 – S2.3**, but with one key distinction: whereas those thresholds correspond to the output of optimal decoding of each area in the case of information-limiting noise, in our extensive information model they correspond to outputs of highly *sub*optimal decoders that are restricted to the noisy subspace of the leading modes, but which are nonetheless optimal within that subspace.
