# Supplementary material for: Inferring decoding strategies for multiple correlated neural populations
Source: PLoS Comput Biol. 2018 Sep 24;14(9):e1006371. doi: 10.1371/journal.pcbi.1006371 (PMC6188888; doi:10.1371/journal.pcbi.1006371)
Supplement: S3 Text — (PDF) [file pcbi.1006371.s020.pdf]

S3 Effects of suboptimal decoding on behavioural threshold

In **S2 Text**, we showed how the optimal thresholds depend on the covariance $\varepsilon_{xx}, \varepsilon_{yy}$, and ε_{xy}. We will now investigate how behavioural thresholds are affected by suboptimal scaling of the different populations.

S3.1 Limited information model: multiple populations

It is convenient to write the combined readout weights of multiple areas as $\mathbf{w} = W\mathbf{a}$ where W is an $N \times Z$ block diagonal matrix $W = \begin{bmatrix} \mathbf{w}_x & \mathbf{0} \\ \mathbf{0} & \ddots \end{bmatrix}$ whose nonzero entries $\mathbf{w}_x$ correspond to the patterns of decoder weights within a single population, each yielding individually unbiased estimates, and elements of $\mathbf{a}$ are the scaling factors on these weights. For unbiased decoding of each population separately, as well as together, we require $W^{\mathrm{T}}F = I$ and $\mathbf{a}^{\mathrm{T}}\mathbf{1} = 1$. Behavioural threshold ϑ is the square root of the decoder variance, which depends on the noise covariance as:

$$\begin{aligned} \vartheta^2 &= \mathbf{w}^{\mathrm{T}}\Sigma_{IL}\mathbf{w} \\ &= \mathbf{w}^{\mathrm{T}}\Sigma\mathbf{w} + \mathbf{w}^{\mathrm{T}}FEF^{\mathrm{T}}\mathbf{w} \\ &= O(N^{-1}) + \mathbf{a}^{\mathrm{T}}W^{\mathrm{T}}FEF^{\mathrm{T}}W\mathbf{a} \\ &\approx \mathbf{a}^{\mathrm{T}}E\mathbf{a} \end{aligned} \qquad \text{(S3.1)}$$

where we have again assumed that the variance from the extensive information part is dominated by the limited information part.

S3.2 Limited information model: two populations

For two populations, **Eqn (S3.1)** yields $\vartheta^2 \approx \mathbf{a}^{\mathrm{T}}E\mathbf{a} = a_x^2\varepsilon_{xx} + a_y^2\varepsilon_{yy} + 2a_xa_y\varepsilon_{xy}$ to give **Eqn (22)** in **Methods**. When the population readout vector $\mathbf{a}$ is suboptimal, the threshold implied by **Eqn (S3.1)** will be smaller than the optimal threshold (**Eqn (22)**). The quadratic form of this equation underlies the U-shaped performance curve shown in **Fig 8B**.

In this formulation, selective inactivation of a neural population simply redefines the population readout vector $\mathbf{a}$. For example, completely inactivating one of two populations corresponds to setting $\mathbf{a} = (0,1)$. Behavioural thresholds following inactivation of either x or y is given by:

$$\vartheta_{-x}^2 \approx (0,1)^T W^{\mathrm{T}}FEF^{\mathrm{T}}W(0,1) \approx \varepsilon_{yy} \qquad \text{(S3.2)}$$

$$\vartheta_{-y}^2 \approx (1,0)^T W^{\mathrm{T}}FEF^{\mathrm{T}}W(1,0) \approx \varepsilon_{xx} \qquad \text{(S3.3)}$$

Therefore, the quality of the decoding is determined by the relative weighting $\mathbf{a}$ of the response in the two populations when both populations are active. However, when one of them is inactivated, the thresholds are near-optimal, limited by noise correlations within the active population.

S3.3 Extensive information model when decoding only dominant noise modes

If decoding is restricted to the single leading eigenmode within each population *x* and *y*, then this mode becomes information-limiting in the restricted decoded space. We can express decoding weights as:

$$\mathbf{w} = \widetilde{U}\mathbf{a}$$

where

$$\widetilde{U} = \begin{bmatrix} \mathbf{u}_x/\mathbf{u}_x^{\mathrm{T}}\boldsymbol{f}_x{}' & \mathbf{0} \\ \mathbf{0} & \ddots \end{bmatrix}$$

is a block diagonal $N \times Z$ matrix containing the leading eigenmode of each area separately, normalized so that $\widetilde{U}^{\mathrm{T}}F = I$ which ensures that the estimators from each population in isolation are unbiased. In this case, behavioural threshold ϑ is once again related to the population readout vector **a** according to:

$$\begin{aligned} \vartheta^2 &= \mathbf{w}^T\Sigma\mathbf{w} \\ &\approx \mathbf{a}^T\widetilde{U}^{\mathrm{T}}FEF^{\mathrm{T}}\widetilde{U}\mathbf{a} \\ &\approx \mathbf{a}^T E\mathbf{a} \end{aligned}$$

which is identical to the limited information case (**Eqns S3.1 – S3.3**) as a function of *E*, because within the decoded subspace the information *is* limited: the extensive information part lies outside. Of course, the values and structure of *E* may differ between the extensive and limited information models, as will the subspace of the full population Σ that is characterized by *E* and the resultant choice correlations.
