# Supplementary material for: Inferring decoding strategies for multiple correlated neural populations
Source: PLoS Comput Biol. 2018 Sep 24;14(9):e1006371. doi: 10.1371/journal.pcbi.1006371 (PMC6188888; doi:10.1371/journal.pcbi.1006371)
Supplement: S4 Text — (PDF) [file pcbi.1006371.s021.pdf]

S4 Effect of suboptimal decoding on choice correlations

In the main text, we derived suboptimal choice correlations for the limited information model (**Eqn (17)**). Here we perform a similar derivation for the extensive information model. If decoding is restricted to the leading eigenmodes within each population, then we can express unbiased decoding weights as $\mathbf{w} = \widetilde{U}\mathbf{a}$ with $\widetilde{U}$ defined in **S3.3 Text**. Substituting this in **Eqn (3)**, we have:

$$\mathbf{C} = \frac{S^{-1}\Sigma\mathbf{w}}{\sqrt{\mathbf{w}^{\mathrm{T}}\Sigma\mathbf{w}}}$$

$$\approx \frac{S^{-1}FEF^{\mathrm{T}}\mathbf{w}}{\sqrt{\mathbf{w}^{\mathrm{T}}\Sigma\mathbf{w}}} = \frac{S^{-1}FEF^{\mathrm{T}}\widetilde{U}\mathbf{a}}{\sqrt{\mathbf{a}^{\mathrm{T}}\widetilde{U}^{\mathrm{T}}FEF^{\mathrm{T}}\widetilde{U}\mathbf{a}}}$$

$$= \frac{S^{-1}FE\mathbf{a}}{\sqrt{\mathbf{a}^{\mathrm{T}}E\mathbf{a}}}$$

If C_{kx} denotes choice correlation of neuron *k* in population *x*, then:

$$C_{kx} = \frac{(S^{-1}FE\mathbf{a})_{kx}}{\sqrt{\mathbf{a}^{\mathrm{T}}E\mathbf{a}}} = \frac{(E\mathbf{a})_x}{\sqrt{\mathbf{a}^{\mathrm{T}}E\mathbf{a}}}(S^{-1}UU^{\mathrm{T}}F)_{kx}$$

$$= \frac{(E\mathbf{a})_x}{\mathbf{a}^{\mathrm{T}}E\mathbf{a}}\vartheta\left(\mathbf{f}_x'^{\mathrm{T}}\mathbf{u}_x\right)(S^{-1}\mathbf{u}_x)_k$$

$$= \beta_x\left[\vartheta\left(\mathbf{f}_x'^{\mathrm{T}}\mathbf{u}_x\right)(S^{-1}\mathbf{u}_x)_k\right] \quad \text{(S4.1)}$$

where the magnitude of choice correlations is given by the multiplier

$$\beta_x = (E\mathbf{a})_x/(\mathbf{a}^{\mathrm{T}}E\mathbf{a}) \quad \text{(S4.2)}$$

For the special case of information-limiting correlations, we substitute $\mathbf{u}_x = \mathbf{f}_x'/||\mathbf{f}_x'||$ in **Eqn (S4.1)** and get:

$$C_{kx} = \beta_x\left[\vartheta\frac{\left(\mathbf{f}_x'^{\mathrm{T}}\mathbf{f}_x'\right)}{||\mathbf{f}_x'||^2}(S^{-1}\mathbf{f}_x')_k\right] = \beta_x\vartheta S_k^{-1}f_k'$$

$$= \beta_x\vartheta\frac{f_k'}{\sigma_k} = \beta_x\frac{\vartheta}{\vartheta_k} \quad \text{(S4.3)}$$

Therefore, in the presence of information-limiting correlations, choice correlations of all neurons from a particular population *x* are a scalar multiple of those resulting from an equivalent optimal decoder with the same behavioural threshold. Different populations could have different multipliers β_x.
