# Supplementary material for: Inferring decoding strategies for multiple correlated neural populations
Source: PLoS Comput Biol. 2018 Sep 24;14(9):e1006371. doi: 10.1371/journal.pcbi.1006371 (PMC6188888; doi:10.1371/journal.pcbi.1006371)
Supplement: S5 Text — (PDF) [file pcbi.1006371.s022.pdf]

S5 Combining choice correlations and inactivation effects

In **S3 Text** and **S4 Text**, we showed how behavioural thresholds (ϑ, ϑ_{-x}, and ϑ_{-y}) and multipliers on choice correlations (β_x and β_y) depend on the relative scaling of weights (a_x and a_y). Now we will combine and invert those results to provide a way to infer the scaling of weights from measurements of thresholds and choice correlations. The ratio of the multipliers β_x/β_y can be written explicitly in terms of the elements of E in **Eqn (S4.2)** in **S4 Text** as:

$$\frac{\beta_x}{\beta_y} = \frac{(E\mathbf{a})_x}{(\mathbf{a}^{\mathrm{T}}E\mathbf{a})}\frac{(\mathbf{a}^{\mathrm{T}}E\mathbf{a})}{(E\mathbf{a})_y} = \frac{(E\mathbf{a})_x}{(E\mathbf{a})_y} = \frac{a_x\varepsilon_{xx} + a_y\varepsilon_{xy}}{a_y\varepsilon_{yy} + a_x\varepsilon_{xy}} \tag{S5.1}$$

S5.1 Uncorrelated populations

If populations *x* and *y* are uncorrelated, then $\varepsilon_{xy} = 0$. Substituting in **Eqn (S5.1)** gives

$$\frac{\beta_x}{\beta_y} = \frac{a_x\varepsilon_{xx}}{a_y\varepsilon_{yy}} \quad \Leftrightarrow \quad \frac{a_x}{a_y} = \frac{\beta_x}{\beta_y}\frac{\varepsilon_{yy}}{\varepsilon_{xx}}$$

If behaviour is indeed largely driven by responses along the leading modes of variance in *x* and *y*, then from **Eqn (S3.2 – S3.3)** in **S3 Text**, the post-inactivation thresholds are $\vartheta_{-x}^2 \approx \varepsilon_{yy}$ and $\vartheta_{-y}^2 \approx \varepsilon_{xx}$. This allows us to express the relative scalings of weights purely in terms of relative magnitudes of choice correlations and inactivation effects.

$$\frac{a_x}{a_y} = \frac{\beta_x}{\beta_y}\frac{\varepsilon_{yy}}{\varepsilon_{xx}} \approx \frac{\beta_x}{\beta_y}\frac{\vartheta_{-x}^2}{\vartheta_{-y}^2} \tag{S5.2}$$

This proves **Eqn (20)** in the main text.

S5.2 Correlated populations

Let populations *x* and *y* be correlated according to $\varepsilon_{xy} = \gamma\varepsilon_{xx}$ where γ denotes the strength of correlations between neurons across the populations relative to those within population *x*. We can re-write **Eqn (S5.1)** as

$$\frac{\beta_x}{\beta_y} = \frac{a_x\varepsilon_{xx} + a_y\gamma\varepsilon_{xx}}{a_y\varepsilon_{yy} + a_x\gamma\varepsilon_{xx}} = \frac{\frac{a_x}{a_y} + \gamma}{\frac{\varepsilon_{yy}}{\varepsilon_{xx}} + \gamma\frac{a_x}{a_y}} \quad \Leftrightarrow \quad \frac{a_x}{a_y} = \left(\frac{\beta_x}{\beta_y}\frac{\varepsilon_{yy}}{\varepsilon_{xx}} - \gamma\right)\left(1 - \frac{\beta_x}{\beta_y}\gamma\right)^{-1}$$

Once again, using $\vartheta_{-x}^2 \approx \varepsilon_{yy}$ and $\vartheta_{-y}^2 \approx \varepsilon_{xx}$, we get:

$$\frac{a_x}{a_y} = \left(\frac{\beta_x}{\beta_y}\frac{\vartheta_{-x}^2}{\vartheta_{-y}^2} - \gamma\right)\left(1 - \frac{\beta_x}{\beta_y}\gamma\right)^{-1} \tag{S5.3}$$

This proves **Eqn (21)** in the main text.
