# Supplementary material for: Inferring decoding strategies for multiple correlated neural populations
Source: PLoS Comput Biol. 2018 Sep 24;14(9):e1006371. doi: 10.1371/journal.pcbi.1006371 (PMC6188888; doi:10.1371/journal.pcbi.1006371)
Supplement: S6 Text — (PDF) [file pcbi.1006371.s023.pdf]

S6 Effect of measurement uncertainty

Neuronal weights $\mathbf{w}$ are related to choice correlations $\mathbf{C}$ and covariance Σ as[3,5]:

$$\mathbf{w} \propto \Sigma^{-1} S \mathbf{C}$$

Without loss of generality, we assume $(\Sigma^{-1} S \mathbf{C})^{\mathrm{T}} \mathbf{f}'=1$, so that decoding is unbiased. Any uncertainty in estimating Σ, S, or $\mathbf{C}$ will all manifest as uncertainty about decoded weights inferred from the above equation. Even under the assumption of a particular noise model (*i.e.* Σ and S are known exactly), uncertainties in measuring $\mathbf{C}$ alone can still give rise to uncertainties in $\mathbf{w}$. To show this, we denote the estimated choice correlation by $\hat{\mathbf{C}} = \mathbf{C} + \delta\mathbf{C}$, where $\mathbf{C}$ is the true choice correlation and $\delta\mathbf{C}$ is the measurement error. The estimated weights $\hat{\mathbf{w}}$ is then given by:

$$\begin{aligned} \hat{\mathbf{w}} = \Sigma^{-1} S \hat{\mathbf{C}} &= \Sigma^{-1} S \mathbf{C} + \Sigma^{-1} S\, \delta\mathbf{C} \\ &= \mathbf{w} + \delta\mathbf{w} \end{aligned}$$

where $\delta\mathbf{w}$ is the error in estimating true weights $\mathbf{w}$**.** Estimation error $\delta\mathbf{w}$ can be expressed in the eigenbasis of Σ as:

$$\delta\mathbf{w} = \Sigma^{-1} S \delta\mathbf{C} = U \Lambda^{-1} U^{\mathrm{T}} S\, \delta\mathbf{C} = U \delta\mathbf{v} = \sum_{i=1}^{N} \delta v_i \mathbf{u}_i$$

where the error in the estimated strength of readout along the direction of the ith eigenvector δv_i is inversely proportional to the corresponding eigenvalue λ_i:

$$\delta v_i = \frac{\mathbf{u}_i^{\mathrm{T}} S\, \delta\mathbf{C}}{\lambda_i} \qquad \text{(S6.1)}$$

Though errors in δv_i are relatively small along directions with large noise variance (large eigenvalues λ_i), they could be amplified enormously along directions with small noise variance (small λ_i). Due to these amplified measurement errors, one can realistically infer only those components of neuronal weights that lie along the first few leading eigenvectors of Σ (**S10 Fig**). If the true readout weights lie largely within the subspace spanned by these components, then the inferred readout will be nearly accurate, and the resultant choice correlations will have magnitudes comparable to the measured ones (see Supplementary modeling section S7 of ref. 3 for proof).
