# Supplementary material for: Inferring decoding strategies for multiple correlated neural populations
Source: PLoS Comput Biol. 2018 Sep 24;14(9):e1006371. doi: 10.1371/journal.pcbi.1006371 (PMC6188888; doi:10.1371/journal.pcbi.1006371)
Supplement: S7 Text — (PDF) [file pcbi.1006371.s024.pdf]

S7 Modeling partial inactivation

The results derived in **S3 Text** and **S5 Text** assumed that inactivation experiments silence all neurons in the target area. In this section, we re-derive the expressions for decoding weights by relaxing this assumption. To accomplish this, we introduce additional parameters ρ_x to model the fractions of neurons in area *x*, that remain following inactivation of those areas. **Eqns (18)** and **(19)** in the main text describe the general effects of partial inactivation in multiple populations. Here we describe the specific consequences for two populations, as applied to our experimental data.

Eqns (S3.2) and **(S3.3)** in **S3 Text** hold when inactivation is complete ($\rho_x = 0$ or $\rho_y = 0$) so that $\mathbf{a} = (0,1)$ or (1,0) depending on whether *x* or *y* was inactivated. If inactivation of *x* was incomplete, then neurons that remain in that area will continue to influence the animal's choice with scaling $\rho_x a_x$, while that of the intact area *y* would become 1–$\rho_x a_x$. Therefore using $\mathbf{a} = (\rho_x a_x, 1 - \rho_x a_x)$ modifies **Eqn (S3.2)** in **S3 Text** to give:

$$\vartheta_{-x}^2 \approx \mathbf{a}^T E\mathbf{a} \approx (\rho_x a_x)^2 \varepsilon_{xx} + (1 - \rho_x a_x)^2 \varepsilon_{yy} + 2\rho_x a_x (1 - \rho_x a_x)\varepsilon_{xy} \quad \text{(S7.1)}$$

Similarly if inactivation of *y* was incomplete, then $\mathbf{a} = (1 - \rho_y a_y,\ \rho_y a_y)$ which modifies **Eqn (S3.3)** in **S3 Text** as:

$$\vartheta_{-y}^2 \approx (1 - \rho_y a_y)^2 \varepsilon_{xx} + (\rho_y a_y)^2 \varepsilon_{yy} + 2\rho_y a_y (1 - \rho_y a_y)\varepsilon_{xy} \quad \text{(S7.2)}$$

The above equations, together with the one that defines choice correlations (**Eqn S5.1** in **S5 Text**), can be used to infer the joint distribution of fractions ρ_x and ρ_y and readout weights that are consistent with experimental data.

Note that **Eqns (S7.1)** and **(S7.2)** are uncoupled if $\varepsilon_{xy} = 0$. This is the case for the extensive information model, and therefore ρ_x and ρ_y are independent for that model (**S15A Fig**). For the limited information model on the other hand, the above equations provide a joint constraint on a_x, ρ_x, and ρ_y and therefore their solutions are correlated (**S15B Fig**).
