# Supplementary material for: Inferring decoding strategies for multiple correlated neural populations
Source: PLoS Comput Biol. 2018 Sep 24;14(9):e1006371. doi: 10.1371/journal.pcbi.1006371 (PMC6188888; doi:10.1371/journal.pcbi.1006371)
Supplement: S8 Text — (PDF) [file pcbi.1006371.s025.pdf]

S8 Recurrent network model

Although all theoretical results on choice correlations are agnostic about the choice of network architecture, the specific behavioural predictions of inactivating either brain area derived in **S5 Text** are not. There, we incorporated the assumption of a purely feedforward model by asserting that the slopes of the tuning curves of neurons in either area remain unchanged following inactivation of the other area. However, in recurrent networks, activity in one area can influence the responses in other areas. If there were recurrent connections between areas *x* and *y*, the lack of lateral inputs following inactivation could alter the responses of neurons in the non-inactivated area, possibly rendering the conclusions drawn from the feedforward model invalid. Here, we show that the main conclusions may nonetheless remain true for at least some recurrent networks. We first derive general results that show how neural response and information content are modified following inactivation in the presence of linear recurrent connections. (Note that this general architecture includes decision feedback as a special case, when the readout weight vector of a population is in the row space of the recurrent weight matrix.) We then focus our analyses on a particular structure of recurrent connections and examine the performance of the network by varying only the connection strength between the two areas to demonstrate our point.

S8.1 Effect of inactivation in recurrent networks

Consider the network shown in **S16A Fig** where responses of neurons in areas *x* and *y* are modulated by a constant stimulus s with gain $\mathbf{g}'_x$ and $\mathbf{g}'_y$ respectively, in addition to receiving inputs from other neurons as determined by the recurrent connectivity matrix *A*. The responses $\mathbf{r}$ are modeled by the following stochastic linear dynamical system:

$$\mathbf{r}_{t+1} = A\mathbf{r}_t + \mathbf{g}'s + \boldsymbol{\eta}_t \tag{S8.1}$$

where the connectivity matrix A is a block matrix given by $A = \begin{bmatrix} A_{xx} & A_{xy} \\ A_{yx} & A_{yy} \end{bmatrix}$, $\mathbf{g}' = (\mathbf{g}'_x, \mathbf{g}'_y)$, $\boldsymbol{\eta}_t \sim \mathcal{N}(0, H)$ is zero-mean noise with covariance *H*, and the subscripts denote discrete time. The steady-state covariance Σ of neural responses is given by the following discrete-time Lyapunov equation:

$$\Sigma = A\Sigma A^{\mathrm{T}} + H \tag{S8.2}$$

and the steady-state mean of the neural response **f**(s) is given by:

$$\mathbf{f}(I - A) = \mathbf{g}'s \tag{S8.3}$$

Note that in the absence of recurrent connections, the response covariance is equal to the covariance of the input noise, i.e. $\Sigma = H$ if $A = 0$. For a given connectivity structure *A*, knowledge of Σ can be used to solve for *H* from the above equation. Covariance in area *x* (or *y*) following inactivation of area *y* (or *x*) can then be obtained by solving:

$$\begin{aligned} \Sigma_{xx} &= A_{xx}\Sigma_{xx}A_{xx}^{\mathrm{T}} + H_{xx} \\ \Sigma_{yy} &= A_{yy}\Sigma_{yy}A_{yy}^{\mathrm{T}} + H_{yy} \end{aligned} \tag{S8.4}$$

Similarly, the slope of the tuning curve, $\mathbf{f}'$ is equal to the input sensitivity $\mathbf{g}'$ if $\boldsymbol{A} = \mathbf{0}$. Otherwise, for a given $\boldsymbol{A}$, sensitivity $\mathbf{g}'$ can be uniquely solved from the slope of the tuning curve as $\mathbf{g}' = \mathbf{f}'(\boldsymbol{I} - \boldsymbol{A})$. The slopes $\mathbf{f}'_x$ and $\mathbf{f}'_y$ following inactivation of area *x* and *y* respectively, can be determined by solving:

$$\mathbf{f}'_x = (I - A)^{-1}\mathbf{g}'_x$$
$$\mathbf{f}'_y = (I - A)^{-1}\mathbf{g}'_y \quad \text{(S8.5)}$$

The above four **Eqn S8.2 – S8.5** together allow us to determine the signals $\mathbf{f}'_x$ and $\mathbf{f}'_y$ and covariances $\boldsymbol{\Sigma}_{xx}$ and $\boldsymbol{\Sigma}_{yy}$ following inactivation, which in turn provide upper bounds on the behavioural thresholds following inactivation: $\boldsymbol{\vartheta}^2_{-x} = \mathbf{1}/\mathbf{f}'_y\boldsymbol{\Sigma}^{-1}_{yy}\mathbf{f}'_y$ and $\boldsymbol{\vartheta}^2_{-y} = \mathbf{1}/\mathbf{f}'_x\boldsymbol{\Sigma}^{-1}_{xx}\mathbf{f}'_x$.

S8.2 Example recurrent network model

Let $[\mathbf{u}_1 \ldots \mathbf{u}_N]$ and $[\mathbf{v}_1 \ldots \mathbf{v}_N]$ denote the set of eigenvectors of Σ_{xx} and Σ_{yy} respectively. We now consider a simple connectivity model in which the connectivity matrix is $A = B\Sigma B^{\mathrm{T}}$ where $B = [\mathbf{u}_1 + \mathbf{v}_1 \quad \mathbf{u}_1 - \mathbf{v}_1 \cdots \mathbf{u}_p + \mathbf{v}_p \quad \mathbf{u}_p - \mathbf{v}_p]$ spans the first p eigenmodes of Σ and $\lambda = [1 + c \quad 1 - c \cdots 1 + c \quad 1 - c]$ are the corresponding eigenvalues, and c denotes the connection strength between the areas. In this scheme, the sum and difference modes are amplified and attenuated respectively for c>0, and vice-versa for $c < 0$. The resulting connectivity structure for extensive and limited information models for $p = 4$ is shown in **S16B Fig**. Using this structure, we used **Eqns S8.2 – S8.5** to evaluate the effect of inactivation for a range of connection strengths for both models. The ratio of behavioural thresholds after inactivation to thresholds before inactivation is shown in **S16D Fig**. We found that inactivation of either area affected behaviour differently depending on the strength of connection between areas. Behaviour is predicted to get worse for both models when the connection was inhibitory, whereas behaviour following inactivation was improved if connections were excitatory and strong. This dependence of inactivation effects on connection allowed us to identify a range of intermediate-strength connections whose inactivation effects were similar to the purely feedforward model, and hence also consistent with our experimental results. For these connection strengths, inactivation of either area amplified the tuning curves slopes in both models (**S16C Fig**). It should be noted that regardless of the choice of connection strength, the recurrent network yields the same covariance in neural response Σ by construction. Consequently, the choice correlations and readout weights of neurons in the recurrent network are identical to those implied by the feedforward model.
