# Supplementary material for: Inferring decoding strategies for multiple correlated neural populations
Source: PLoS Comput Biol. 2018 Sep 24;14(9):e1006371. doi: 10.1371/journal.pcbi.1006371 (PMC6188888; doi:10.1371/journal.pcbi.1006371)
Supplement: S9 Text — (PDF) [file pcbi.1006371.s026.pdf]

S9 Effect of selective inactivation on choice correlations in the non-inactivated area

Since choice correlation depends both on the neuron's own readout weight as well as the weights of other neurons with which it is correlated [3], silencing those other neurons is bound to have an effect on its choice correlation. For this reason, when information is distributed across correlated populations, selectively inactivating one of them will naturally affect choice correlations in the non-inactivated area. Here we consider two populations *x* and *y* and show how choice correlations in each should change following inactivation of the other area. From **Eqn (S4.1)** in **S4 Text**, choice correlation of neuron *k* within population *x* (when both *x* and *y* are active) is given by

$$C_{kx} = \frac{(E\mathbf{a})_z}{\mathbf{a}^{\mathrm{T}}E\mathbf{a}}\sqrt{\mathbf{a}^{\mathrm{T}}E\mathbf{a}}(S^{-1}F)_{kx} = \beta_x\vartheta(S^{-1}F)_{kx} \tag{S9.1}$$

If population *y* is inactivated so that only *x* is active, then $a_x = 1$ and $a_y = 0$ so $\mathbf{a} = (1,0)$. Substituting this in **Eqn (S4.1)** in **S4 Text**, we obtain choice correlation $\tilde{C}_{kz}$ of neuron *k* in area *x* when *y* is inactivated:

$$\begin{aligned}\tilde{C}_{kx} &= \frac{(E(1,0))_x}{(1,0)^{\mathrm{T}}E(1,0)}\sqrt{(1,0)^{\mathrm{T}}E(1,0)}(S^{-1}F)_{kx} \\ &= \vartheta_{-y}(S^{-1}F)_{kx}\end{aligned} \tag{S9.2}$$

From **Eqns (S9.1)** and **(S9.2)**,

$$\tilde{C}_{kx} = \vartheta_{-y}(S^{-1}F)_{kx} = \zeta_x C_{kx} \tag{S9.3}$$

where $\zeta_x = \frac{1}{\beta_x}\frac{\vartheta_{-y}}{\vartheta}$. Similarly, we can show that

$$\tilde{C}_{ky} = \zeta_y C_{ky} \tag{S9.4}$$

where $\zeta_y = \frac{1}{\beta_y}\frac{\vartheta_{-x}}{\vartheta}$. **Eqns (S9.3)** and **(S9.4)** constitute quantitative predictions for how choice correlations should rescale upon inactivating each area. This proves **Eqn (25)** (**Methods**).
